# Human REV1 interacts with DHX36 to promote replication and tolerance of G-quadruplex DNA

**DOI:** 10.1093/nar/gkag562

**Published:** 2026-06-08

**Authors:** Amit Ketkar, Bethany C Paxton, Oscar E Zuniga, Reham S Sewilam, Martin Morales, John Schaller, Rowan J McCollum, Kathleen E Jackson, Kaitlin Lowran, Alyssa Paul, Meera Patel, Sreevatsav Seenivasan, Mason McCrury, Qudes Al-Anbaky, Leena Maddukuri, Samantha Kendrick, Colin G Wu, Julie E C Gunderson, Robert L Eoff

**Affiliations:** Department of Biochemistry and Molecular Biology, University of Arkansas for Medical Sciences, Little Rock, AR 72205,United States; Department of Biochemistry and Molecular Biology, University of Arkansas for Medical Sciences, Little Rock, AR 72205,United States; Department of Biochemistry and Molecular Biology, University of Arkansas for Medical Sciences, Little Rock, AR 72205,United States; Department of Biochemistry and Molecular Biology, University of Arkansas for Medical Sciences, Little Rock, AR 72205,United States; Department of Biochemistry and Molecular Biology, University of Arkansas for Medical Sciences, Little Rock, AR 72205,United States; Department of Biochemistry and Molecular Biology, University of Arkansas for Medical Sciences, Little Rock, AR 72205,United States; Department of Biochemistry and Molecular Biology, University of Arkansas for Medical Sciences, Little Rock, AR 72205,United States; Department of Biochemistry and Molecular Biology, University of Arkansas for Medical Sciences, Little Rock, AR 72205,United States; Department of Chemistry, Oakland University, Rochester, MI 48309,United States; Department of Chemistry, Oakland University, Rochester, MI 48309,United States; The Science Department, Arkansas School for Mathematics, Sciences, and the Arts, Hot Springs, AR 71901,United States; Department of Biochemistry and Molecular Biology, University of Arkansas for Medical Sciences, Little Rock, AR 72205,United States; Department of Biochemistry and Molecular Biology, University of Arkansas for Medical Sciences, Little Rock, AR 72205,United States; Department of Biochemistry and Molecular Biology, University of Arkansas for Medical Sciences, Little Rock, AR 72205,United States; Department of Biochemistry and Molecular Biology, University of Arkansas for Medical Sciences, Little Rock, AR 72205,United States; Department of Biochemistry and Molecular Biology, University of Arkansas for Medical Sciences, Little Rock, AR 72205,United States; Department of Chemistry, Oakland University, Rochester, MI 48309,United States; Department of Physics, Hendrix College, Conway, AR 72032, United States; Department of Biochemistry and Molecular Biology, University of Arkansas for Medical Sciences, Little Rock, AR 72205,United States

## Abstract

G-quadruplex DNA is a barrier to replication, but how the replisome couples G4 bypass with fork progression is not well-defined. Here, we establish that REV1 is central to coordination of G4 resolution, replication fidelity, and tolerance of G4 stabilization. REV1 loss switched fork elongation to a PrimPol-driven mechanism and resulted in defective ssDNA gap suppression in cells treated with pyridostatin (PDS). Mutagenic G4 replication on the leading strand was more impacted by REV1 loss than lagging strand bypass, but only lagging strand mutagenesis was sensitive to PDS. REV1 deficiency increased nuclear G4 signal, amplified ATM/ATR signaling, and sensitized cells to G4-stabilizing agents. We discovered that the REV1 C-terminal domain interacts with the G4 helicase DHX36 to restrain PrimPol activity. The REV1-DHX36 interaction is direct and requires a newly defined REV1-interacting region at the C-terminus of DHX36. Prolonged G4 stabilization uncoupled REV1 and DHX36, with the G4 helicase accumulating at a site distal from REV1 and the DNA synthesis machinery. Our findings establish a two-tiered mechanism for REV1 action that coordinates helicase-dependent G4 unwinding with suppression of ssDNA gaps in response to G4 stabilization.

## Graphical Abstract

**Graphical Abstract:**
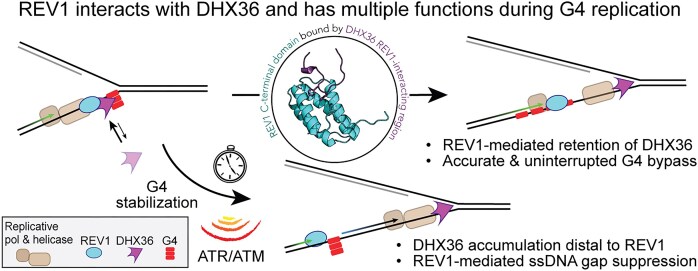

## Introduction

Replication forks inevitably encounter blocks to fork progression and must adapt in multiple ways to ensure faithful copying of the genome [1, 2]. Non-canonical DNA structures can take many forms that challenge uninterrupted and accurate DNA synthesis. Guanine-rich nucleic acid sequences that can adopt four-stranded G-quadruplex (G4) structures represent a significant barrier to fork progression in organisms across the spectrum of life [3–5]. The challenges of copying G4 DNA are many and include the repetitive nature of the motif, diverse folding patterns, and relative stability of these non-canonical structures, as well as the tendency for tetrad guanines to become oxidized [6–8]. Multiple pathways have been implicated in G4 replication and maintenance, including translesion synthesis (TLS) [5], but important mechanistic aspects of these events remain poorly defined.

TLS is an evolutionarily ancient means of tolerating DNA damage and bypassing natural fork barriers through utilization of specialized DNA polymerases (pols) [9, 10]. The Y-family DNA pols are counted amongst the cadre of TLS enzymes, and they represent an especially versatile means of bypassing fork barriers in the template strand and enduring changes in the nucleotide pool [11]. TLS pols are an integral part of acquired resistance mechanisms in both prokaryotes and eukaryotes. Bacteria call upon TLS enzymes to survive antibiotics [12], while tumor cells sometimes upregulate TLS in response to cues from the surrounding microenvironment and following exposure to genotoxic therapies [13–15]. Determining how and when TLS contributes to DNA replication programs continues to reveal new and surprising features of this genome maintenance pathway.

Reversionless 1 (REV1) DNA pol is a Y-family member that is conserved from fungi through mammals and has been implicated in mutagenic replication [16], including connections with cytosine deaminase-mediated mutagenesis [17] and bypass of DNA adducts [18]. REV1 is unusual, even amongst the eclectic Y-family enzymes, because it uses a protein-template-directed mechanism of action when catalyzing nucleotide insertion, effectively serving as a cytidyl transferase [19, 20]. A major non-catalytic role for REV1 involves acting as a scaffold for recruitment of other TLS factors (e.g. pols η, κ, and ζ) to sites of impeded fork progress [21–23]. The scaffolding function of REV1 primarily involves interactions at its C-terminal domain (CTD) with some contributions from the N-terminal region, including the BRCT domain [22, 24–26]. While much has been reported about bypass of DNA damage, it has been increasingly appreciated that REV1 is a central player in copying non-canonical nucleic acids.

Well over a decade ago, studies from the Sale laboratory using avian cells established REV1 as a mediator of fork progression and epigenetic stability near G4 sites [27]. Additional experiments discovered that REV1 coordinated with the FANCJ helicase to help accomplish G4 bypass [28]. Later, we reported that loss of human REV1 increased the mutation frequency at a G4 motif relative to a non-G4 control sequence, highlighting the fact that an “error-prone” TLS enzyme could in fact limit mutagenesis under certain conditions [29]. Additional work with purified enzyme identified G4-selective features of REV1 activity [29–31]. For example, REV1 binds more tightly to G4 than non-G4 DNA substrates (especially parallel-stranded G4s), and REV1 orthologs from human and zebrafish appear to have some capacity to disrupt tetrads without performing DNA synthesis, a feature that seems to rely on residues in the so-called “G-loop” formed in part by the insert-2 motif. Mutation of E466 and Y470 to alanine largely ablates the G4 selective attributes of human REV1 [29, 31]. Insert-2 mutants appear to be less capable of restoring the accuracy of G4 replication than WT REV1 [29], further supporting the idea that G4 selective interactions are key to REV1 function during G4 TLS. The exact nature of REV1 activity and the protein–protein interactions involved in effective G4 TLS remains unclear.

In the current study, we identify key outcomes of REV1 loss for the G4 replication machinery and downstream effects on G4 dynamics. The use of mass spectrometry to analyze replication-associated proteins then provided us with unbiased and global insights into protein–protein interactions involved in bypass of barriers to fork progression. From our results, we discovered new roles for REV1 in G4 maintenance and tolerance of G4 stress, including a previously unknown interaction with DHX36 that facilitates retention of the G4 helicase on chromatin near sites of nascent strand synthesis. Our results provide insight into the mechanism by which human REV1 protects against G4-induced mutation and genomic instability by interacting with one of the most potent G4 helicases in eukaryotes—DHX36.

## Materials and methods

### Reagents

All chemicals and reagents used were molecular biology grade or better. Oligonucleotides used for cloning and mutagenesis were purchased from Integrated DNA Technologies (Coralville, IA). Pyridostatin (PDS) was purchased from Chemietek (Chemietek, Indianapolis, IN). PDS was resuspended in dimethyl sulfoxide (DMSO) for clonogenic survival assays and 1× phosphate buffered saline (PBS) for all other experiments. 3,3′-[1,10-Phenanthroline-2,9-diylbis(carbonylimino)]bis[1-methylquinolium]1,1,1-trifluoromethanesulfonate (1:2) (PhenDC3) and N-methyl mesoporphyrin IX (NMM) were purchased from Sigma (Burlington, MA). PhenDC3 and NMM stock solutions were prepared in 100% DMSO. We purchased 5-ethynyl-2′-deoxyuridine (EdU) from Sigma–Aldrich (St. Louis, MO). EdU stock solutions were prepared in 100% DMSO (Sigma–Aldrich).

### Antibodies

Primary antibodies were used as follows: REV1 (Santa Cruz #sc-393022; 1:100 western blot and 1:25 immunofluorescence), DHX36 (Abcam #ab70269; 1:2000 western blot and 1:50 PLAs, 1:200 SIRF assays), anti-biotin (Sigma Millipore #B7652), GAPDH (Cell Signaling #2118; 1:10000 western blot), anti-FLAG M2 (Sigma–Aldrich, #F1804; 1:2000 western blot and 1:400 immunofluorescence), Bromodeoxyuridine [BrdU] (BD Biosciences #347580; mouse, 1:250 DNA fiber spreading), PCNA (Cell Signaling #2586; 1:2000 western blot), histone H3 (Cell Signaling #9715; 1:1000 western blot), Rev7/MAD2L2 (Abcam #180579; 1:1000 western blot), HLTF (Abcam #183042; 1:1000 western blot), METTL3 (Abcam #195352; 1:1000 western blot), ATM (Cell Signaling #2873; 1:1000 western blot), TopBP1 (Cell Signaling #14342; 1:1000 western blot), NOP53/GLTSCR2 (Cell Signaling #73225; 1:1000 western blot), Primpol/CCDC111 (Invitrogen #MA5-32899; 1:1000 western blot), phosho-CHK1 [S296] (Abcam #ab79758; 1:1000 western blot), CHK1 [total] (Santa Cruz Biotech #; 1:500 western blot), phospho-CHK2 [T68] (Cell Signaling #2661; 1:1000 western blot), and CHK2 [total] (Santa Cruz Biotech #; 1:500 western blot).

### Cell lines

HEK293 Flp-In™ T-Rex™ (293 FT) cells were purchased from ThermoFisher Scientific (#R78007). U2OS cells were purchased from ATCC (#HTB-96). HAP1 WT and *REV1*^KO^ cells were purchased from Horizon Discovery (#C631). 293 FT and U2OS cells were cultured in Dulbecco’s modified Eagle medium (DMEM) (Gibco, #11-965-092) supplemented with 10% (v/v) fetal bovine serum (Corning, #A4766801). HAP1 cells were cultured in Iscove’s modified Dulbecco’s medium (IMDM) (Gibco, #12440-053) supplemented with 10% (v/v) fetal bovine serum. Cells were incubated at 37°C with 5% CO_2_. All cell lines tested negative for mycoplasma approximately every 6 months (ATCC, #30-1012K).

### Generation of HEK293 FT *REV1*^KO^ cell line using CRISPR–Cas9

CRISPR–Cas9 was used to generate 293 FT *REV1*^KO^ cells as previously described [32]. Briefly, two single guide RNAs (sgRNAs) targeting *REV1* with minimal off-target effects were designed using the ATUM Bio gRNA Design Tool: https://www.atum.bio/catalog/vectors/grna-design (Supplementary Fig. S1):

sgRNA 1:

REV1_gRNA_276F: 5′ - CACCgTTATAGGTTCTGCCGTGGCT - 3′
REV1_ gRNA_276R: 5′ - AAACAGCCACGGCAGAACCTATAAc - 3′

sgRNA 2:

REV1_ gRNA_256F: 5′ - CACCgCTTTCCTTTTCAGTTCGAAC - 3′
REV1_ gRNA_256R: 5′ - AAACGTTCGAACTGAAAAGGAAAGc - 3′

The sgRNA oligos were cloned into a pSpCas9n(BB)-2A-Puro plasmid (Addgene plasmid ID: 48141). The plasmids were transformed in DH5$\alpha $  *Escherichia coli* (MCLab, #DA-196). Transformed colonies were selected for by ampicillin. The plasmids were purified using a QIAprep spin miniprep kit (Qiagen, #27104) and cloning of the sgRNA sequence was confirmed by Sanger sequencing. 293 FT parental cells were transfected with the plasmid using Lipofectamine-3000 (Thermo Fisher Scientific) for 24 h and selected with 2 µg/ml puromycin. Selection antibiotic was maintained for 48 h, following which media were aspirated out to remove dead cells. Survivors continued to be cultured in complete DMEM medium for another 24 h. At the end of the 24 h recovery, surviving cells were counted, diluted, and then seeded in 96-well culture dishes at a density of 1–2 cells/well. The dishes were monitored every 2–3 days for colony growth and medium was replaced. The wells with measurable colony growth were expanded further by culturing in larger wells and dishes. Finally, when the clones were grown to confluency on a 100 mm dish, they were cryo-preserved. An aliquot of each clone was taken for cell lysis, followed by confirmation of REV1 knockout using a PCR-based readout (Supplementary Fig. S1B) or immunoblotting (Supplementary Fig. S1C). Two validated *REV1*^KO^ clones (6D6 and 4H8) were used in this study.

### Engineering Dox-inducible expression of WT and mutant REV1 in 293 FT *REV1*^KO^ cells

The human REV1 gene was cloned into the pcDNA5/FRT/TO vector to express the full-length WT protein (aa 1–1251) with an S-protein/2×FLAG/streptavidin-binding peptide (SFB) affinity tag encoded at the N-terminus (Supplementary Fig. S2A). Site-directed mutagenesis was used to generate the REV1 E466A/Y470A (EY/AA) and REV1^1–1040^ (ΔCTD) expression plasmids. The primers used for mutagenic PCR were as follows: E466A-Forward primer 5′-CCCAGCTGGCGTGGCAGTATTAC-3′’; Reverse primer 5′*-*GTAATACTGCCACGCCAGCTGGG-3′. Y470A-Forward primer 5′-GGCAGTATGCCCAGAATAAAATC-3′; Reverse primer 5′-GATTTTATTCTGGGCATACTGCC-3′. ΔCTD-Forward primer 5′-GATCAAAGACAATAATAGGGCGAGAAC-3′; Reverse primer 5′-GTTCTCGCCCTATTATTGTCTTTGATC-3′. Mutations were confirmed by Sanger sequencing of the plasmids. Each of these plasmids was independently transfected into the 6D6 *REV1*^KO^ clone of the 293 FT cells. Transfectants were selected by growing in DMEM containing hygromycin (20–40 μg/ml). Surviving clones were picked and expanded separately to obtain high cell density. Small aliquots were set aside to confirm positive clones, and the rest of the cells were cryo-preserved. To induce expression, the aliquot of cells was treated with doxycycline at 0.2 µg/ml for 48 h, with parallel aliquots grown without doxycycline treatment as negative controls. Cells were harvested and pelleted 48 h after induction, and lysed. Proteins from Flp-In lysates were separated by sodium dodecyl sulfate–polyacrylamide gel electrophoresis (SDS–PAGE) and induction was confirmed by immunoblotting with anti-FLAG antibody to confirm expression (Supplementary Fig. S2B).

### siRNA knockdown

Target siRNA and Lipofectamine 3000 were used in accordance with manufacturer instructions. Briefly, 80 pmoles of the target siRNA was combined with 18 µl Lipofectamine 3000 (Thermo Fisher Scientific, #L3000015) in OptiMEM (Gibco, #31-985-070) in 60 mm dishes. The transfection reagent was incubated at room temperature for 20 min before adding to cells for 24 h before splitting for experiments. Immunoblotting was performed to confirm knockdown. The non-targeting control siRNA (SCR) was purchased from Dharmacon (#D-001810-10-20).

siRNA sequences purchased from Dharmacon:

DHX36 (#170506):

J-01367-09: CGGCAUGUGGUACGCGAAA
J-01367-10: GAUCAUAUAUUGACCGAGA
J-01367-11: GUAAGGGAACUGCGAAGAA
J-01367-12: AGAAGAGGAUGGUGCGAUA

PRIMPOL (#201973)

J-02146-05: GAAAAGGCUACAGAGGAAU
J-02146-06: AAAGAAACUGGAGAGGCUU
J-02146-07: AGGAAAGCUGGACAUCGAU
J-02146-08: AGAAUAACAUGGGAGAGAU

### DNA fiber spreading assays

The DNA fiber spreading (DFS) experiments followed the general protocol reported by others [33]. Briefly, six-well plates were coated with 0.01% (w/v) poly-L-lysine (Sigma Millipore, #A-005-C) for 24 h. Then 293 FT WT or *REV1*^KO^ were plated and allowed to grow for 24 h. Where indicated, the “flipped-in” 293 FT cell lines engineered to express REV1 (WT, EY, and ΔCTD) were treated with 0.2 μg/ml doxycycline for 48 h prior to labeling with thymidine analogues, with parallel aliquots grown without doxycycline treatment as negative controls. Cells were treated with 20 μM 5-chloro-2′-deoxyuridine (CldU; Sigma–Aldrich, #C6891) for 30 min at 37°C. Cells were washed with pre-equilibrated 1× PBS before adding 100 μM 5-iodo-2′-deoxyuridine (IdU) with either vehicle (1× PBS) or 50 μM PDS for 1 h at 37°C. Cells were washed with pre-equilibrated 1× PBS prior to permeabilization with CSK100 buffer consisting of 10 mM MOPS (pH 7), 100 mM NaCl, 3 mM MgCl_2_, 300 mM sucrose, and 0.5% (w/v) Triton-X 100 for 10 min at room temperature. Wells were carefully washed with 1× PBS prior to the addition of S1 nuclease buffer containing 20 U/ml S1 nuclease, 30 mM sodium acetate (pH 4.6), 10 mM zinc acetate, 5% (v/v) glycerol, and 50 mM NaCl for 30 min at 37°C. For control conditions, cells were treated with S1 nuclease buffer without S1 nuclease. After S1 nuclease incubation, cells were washed in 1× PBS containing 0.1% (w/v) BSA and harvested using a cell scraper. Cells were pelleted by centrifugation at 4000 × *g* for 5 min at 4°C. The supernatant was removed, and the pellet was resuspended in ice-cold 1× PBS. Cells were counted and diluted to 1 million cells/ml and mixed 1:1 with unlabeled cells. Cell suspension (2 μl) was pipetted onto microscope glass slides. DNA lysis buffer [10 μl; 0.5% (w/v) SDS, 200 mM Tris–HCl pH 7.4, 50 mM ethylenediaminetetraacetic acid (EDTA)] was added to the drop on the slide and incubated for 5 min at room temperature. Slides were then tilted to ∼45° to allow the DNA to stretch on the slide and dried for at least 40 min at room temperature. Dried slides were fixed in 3:1 dilution of methanol and acetic acid for 5 min before incubating in 2.5 M HCl for 70 min. Slides were washed in 1× PBS three times and transferred to humid boxes where they were incubated in blocking buffer (10%, v/v, goat serum in 1× PBS) for 60 min at room temperature. Primary antibody solution (1:100 rat anti-bromodeoxyuridine, Novus Biologicals, Centennial, CO) and 1:250 mouse anti-BrdU (BD Biosciences, San Jose, CA) in 1× PBS containing 10% (v/v) goat serum was pipetted onto the slides and incubated for 2 h. Slides were washed, and secondary antibody solution [1:250 goat anti-rat IgG AlexaFluor 594 (Invitrogen, Carlsbad, CA) and 1:250 goat anti-mouse 488 conjugate (Invitrogen, Carlsbad, CA) in 10% (w/v) BSA in 1× PBS] was added to slides and incubated for 60 min in the dark at room temperature. The length of CldU and IdU was measured for each fiber counted. Images were obtained via the Zeiss LMS 880 microscope, blinded, and quantified. Two independent biological replicates were performed for each condition. The number of fibers quantified per condition is noted in the figure legends. Results were plotted in Prism (GraphPad, San Diego, CA). A Kruskal–Wallis test followed by a Dunn’s multiple comparison test was used to evaluate differences between conditions.

### Native CldU immunofluorescence measurements

We followed the general protocol reported by others [34]. Briefly, coverslips were coated with 0.01% (w/v) poly-L-lysine for 24 h. Then 293 FT WT or *REV1*^KO^ (6D6) cells were plated on glass coverslips in six-well plates. The cells were incubated with 100 µM CldU 24 h after plating. After 24 h of incubation with CldU, the cells were incubated with 50 μM PDS for 4 h. Then the coverslips were incubated with media containing 20 μM EdU for 2 h. The coverslips were washed and then fixed with 3.7% (v/v) formaldehyde and permeabilized with 1× PBS containing 0.2% (w/v) Triton X-100. Click chemistry was performed by adding 10 μM Alexa Fluor488^TM^, 20 mg/ml sodium ascorbate, and 100 mM CuSO_4_ to a solution of 1× PBS and incubating on the coverslips for 30 min at room temperature. Coverslips were blocked in 10% (v/v) goat serum for 30 min at room temperature. To detect CldU signal, anti-BrdU rat monoclonal antibody (Abcam cat #6326) was added and incubated for 1 h at room temperature. Coverslips were washed with 1× PBS, and goat anti-rat IgG Alexa Fluor594^TM^ (Invitrogen cat #A11007) antibody was added for 1 h at room temperature. Coverslips were washed and mounted with ProLong Gold antifade mounting solution with DAPI (cat #P36931) before imaging on a Zeiss LSM 880 confocal microscope. Results were quantified using ImageJ and CellProfiler^TM^. Three technical replicates were performed and at least 1800 cells were quantified in total per condition across three coverslips. *P*-values were calculated using a one-way ANOVA followed by Tukey’s multiple-comparisons test.

### *supF* mutagenesis experiments

The *supF* mutagenesis experiments were performed largely as we described them in a previous publication [29]. Briefly, the 18-nucleotide G4-forming sequence derived from the *c-Myc* promoter was introduced immediately upstream of the *supF* tRNA coding region in the pSP189 plasmid. Since the G4 motif was placed in the lagging strand with respect to the replisome direction, the G4-containing plasmid was termed “Lag-G4.” Another modified pSP189 plasmid was generated, where the 18-mer Myc-G4 sequence was introduced in the leading strand, immediately 3′ to the *supF* coding region. The resulting plasmid was termed the “Lead-G4.” The WT pSP189 plasmid served as a non-G4 control.

Each plasmid was transfected separately into WT or *REV1*^KO^ HAP1 cells. The HAP1 cells were cultured in IMDM (Millipore-Sigma, St. Louis, MO, USA) supplemented with 10% (v/v) fetal bovine serum and 1% (v/v) antibiotics containing 100 units/ml penicillin, 100 mg/ml streptomycin, and 0.25 mg/ml amphotericin B (Sigma–Aldrich, St. Louis, MO, USA). Cells were cultured in an incubator at 37°C in an atmosphere containing 5% (v/v) CO_2_. Transfection was performed using Lipofectamine-3000 (Thermo Fisher, USA) according to the manufacturer’s instructions. The transfected cells were cultured either in the presence or absence of the G4-stabilizing chemical PDS (0.5 µM) for 24 h and then harvested. The pSP189 plasmids (control or G4) were extracted from cell pellets using the plasmid miniprep kit (Qiagen). Following treatment with the Dpn I endonuclease to digest parental (methylated) DNA, the plasmids were then used to transform the MBM7070 indicator strain of *E. coli*, and transformants were plated on LB-agar plates containing 100 mg/ml ampicillin, 0.12 mg/ml 5-bromo-4-chloro-3-indolyl-β-D-galactopyranoside (X-Gal) and 1 mM isopropyl-β-D-1-thiogalactopyranoside (IPTG). After incubation at 37°C for 15 h, blue and white colonies were observed on the plates. If necessary, the plates were incubated overnight at 4°C to allow the blue color to intensify. The number of blue (unmutated) and white (mutated) colonies was counted for each plate using the Fiji version of ImageJ software. Mutation frequency was determined for each experimental sample by plotting the ratio of number of white colonies for a sample to the total number of colonies (blue and white) for that sample. Each experimental condition was performed in biological triplicate. For every replicate, at least 10 000 colonies were counted.

### BG4 immunofluorescence

Cells were plated on coverslips coated with 0.01% (w/v) poly-L-lysine (Sigma Millipore, #A-005-C) in six-well plates and allowed to attach overnight. The next day, the cells were treated with PDS for 24 h. After PDS treatment, the cells were fixed with 3.7% (v/v) formaldehyde for 25 min at room temperature. The cells were permeabilized with 0.2% (w/v) Triton X-100 in 1× PBS for 40 min at 4°C. The coverslips were transferred to humid boxes and blocked with blocking buffer containing 3% (w/v) bovine serum albumin, 1.5% (w/v) goat serum, and 0.1% (w/v) Triton X-100 in 1× PBS. The coverslips were treated with 0.1 mg/ml RNase (Invitrogen, # 12091-021) in blocking buffer for 80 min at room temperature. Digested RNA was washed away with 1× PBS before incubating the coverslips with 45 nM BG4 in blocking buffer overnight at 4°C. The next day, the coverslips were washed with 1× PBS with 0.1% (w/v) Tween-20 (PBST), then anti-FLAG antibody (Sigma–Aldrich, #F1804) was added at a 1:400 dilution in blocking buffer for 80 min at room temperature. For experiments in HAP1 cells, the coverslips were washed with PBST and incubated with anti-mouse 650-conjugated antibody diluted 1:286 in blocking buffer for 1 h 20 min at room temperature (ThermoScientific, #84545). For experiments with the 293 FT cells, the coverslips were washed with PBST and incubated with goat anti-mouse Alexa Fluor^TM^ 594-conjugated antibody diluted 1:500 in blocking buffer for 1 h 20 min at room temperature (ThermoScientific, #A-11032). The coverslips were washed with PBST and washed once with deionized H_2_O before staining with DAPI (Invitrogen, #P36931). The slides were cured overnight in the dark at room temperature and were stored at −20°C. Images for HAP1 cells were captured using an Olympus IX81 confocal microscope with a 60× PLAPON60XO objective using FV10-ASW 4.02 software. Images for 293 FT cells were captured on a Nikon Eclipse Ti2 confocal with a Nikon CFI Plan Apo Lambda 60× objective and NIS-Elements AR 5.42.03 software. BG4 intensity was measured using Cell Profiler™ 4.2.1 (sites.broadinstitute.org/cellprofiler/).

### Clonogenic survival assay

293 FT and HAP1 cells were plated in six-well plates at an initial density of 1500 and 3000 cells per well, respectively. For the experiments with siRNA-knockdown of DHX36, 2 × 10^6^ cells were initially plated on a 10 cm dish and allowed to attach for 24 h. Cells were then subjected to the target siRNA treatment using Lipofectamine-3000 as per manufacturer’s instructions, followed by a further incubation for 24–48 h. Cells were then harvested, counted, and then plated in six-well plates as described above. For the Flp-In cells, the respective Flp-In 293FT cells (expressing WT, EY/AA, or Rev1-ΔCTD) were seeded in six-well plates at an initial density of 1500 cells per well in a medium containing doxycycline (0.2 μg/ml) and allowed to attach for 24 h. The cells were treated with a range of concentrations (0 to 100 µM) for each G4-stabilizer (PDS, NMM, or PhenDC3) for 4 h. For the clonogenic survival assays, all G4 stabilizers were prepared in DMSO so that the same vehicle control treatment could be used. After treatment, the drug was removed by washing the cells three times with 1× PBS. The cells were allowed to grow for 8–10 days before fixing and staining using Coomassie stain solution containing 0.2% (w/v) BB-G250, 30% (v/v) methanol, 20% (v/v) acetic acid for 20 min at room temperature. The dishes were rinsed in warm water to wash off excess stain and dried on paper towels overnight. Colonies were visualized using an Odyssey CLx LI-COR imager. Colonies were counted using the colony-counter tool in ImageJ. Percent survival was calculated by dividing the number of colonies counted by the number of cells plated multiplied by the plating efficiency. The resulting survival data were fit to a log[inhibitor] versus response four-parameter variable slope model using Prism (GraphPad). Statistical significance was assessed by performing unpaired Student’s t-tests for calculated IC_50_ values.

### Isolation of proteins on nascent DNA

Isolation of proteins on nascent DNA (iPOND) was performed according to the protocol described previously [35, 36]. Briefly, 293 FT cells (parental and *REV1*^KO^) were cultured and expanded to obtain 1 × 10^8^ cells in five 150 mm dishes per experimental condition. In addition to the EdU “pulse” and a thymidine “chase” sample, both cell lines were subjected to treatment with PDS for 15 min or 60 min. An additional EdU pulse sample was also included as a “no-click” negative control. The pulse was by treatment of the cells with 10 µM EdU for 10 min. The chase sample was where cells, after the EdU pulse, were rinsed with sterile PBS, followed by culturing in the presence of 10 µM thymidine for an additional 60 min. At the end of each treatment, the samples were treated with 1% (v/v) formaldehyde in PBS for 10 min for fixing and crosslinking the chromatin-bound proteins. Reactions were quenched by adding 625 mM glycine, and the cells were collected by scraping them off the culture dishes. After washing the cells three times with PBS to remove formaldehyde and glycine, the cells were permeabilized before being subjected to the “click” reaction using 10 µM biotinylated azide reagent. The “no-click” sample was treated with DMSO instead of biotin-azide reagent. After allowing the click reaction to proceed in the dark for 1 h at room temperature, the cells were washed with PBS and dried by inverting on paper towels. This was followed by cell lysis and sonication to shear the protein-bound chromatin DNA into fragments. After keeping aside an aliquot from each sample as “input” control, the rest of each sample was allowed to mix with streptavidin-agarose beads pre-equilibrated in the “capture” buffer and incubated on a rotary mixer overnight at 4°C to allow chromatin-bound proteins to be captured on the beads. The samples were then washed in a series of wash buffers before adding a 2× Laemmli buffer to each sample to reverse the crosslinks and release the chromatin-bound proteins. Samples were then submitted for analysis by mass spectrometry (MS) to the IDeA National Resource for Quantitative Proteomics at UAMS. Another set of samples was processed exactly as described above and used for immunoblotting to be probed for various protein targets. The iPOND-MS analysis was performed on four biological replicates for each cell line. Briefly, tryptic peptides were analyzed using liquid chromatography (LC) followed by electrospray ionization MS on an Orbitrap Exploris 480 mass spectrometer (Thermo). Data-independent acquisition (DIA) was used and quantitative analysis of spectra was performed. The full details of sample processing and data processing can be found in the ProteomeXchange deposition PXD061281 at proteomexchange.org. The mass spectrometry data can also be found in Supplementary Table S1.

### Immunoblotting

Cell pellets were lysed for 45 min at 4°C in 40 mM HEPES (pH 7.5) buffer containing 120 mM NaCl, 1 mM EDTA, 50 mM NaF, 1 mM Na_3_VO_4_, 10 mM sodium β-glycerophosphate, and 1% (v/v) Triton X-100 with 1× protease inhibitor cocktail (Sigma–Aldrich). The lysates were centrifuged for 15 min at 21 000 × *g* at 4°C. The protein concentration of each sample was estimated using a Pierce BCA protein assay kit (ThermoFisher, #23225). Samples were run on tris-glycine or tris-acetate gels (Invitrogen, #XP04205, #EA0375). Proteins were transferred onto PVDF membranes at 200 mA for 1.25 h at 4°C. The blots were blocked in a solution of 5% (w/v) milk in 1× Tris-buffered saline (TBS), and primary antibodies were added overnight at 4°C in a solution of 0.5% (w/v) milk in 1× TBS. The next day, blots were washed with 1× TBS with 0.1% (w/v) Tween-20 (TBST) before incubation with mouse or rabbit HRP-conjugated secondary antibody (Invitrogen, #32430, #32460) at room temperature for 1.5 h. The blots were washed again with TBST. The blots were developed using enhanced chemiluminescence (ECL) (BioRad, #1705060). Protein bands were visualized on a ChemiDoc digital imager (BioRad, #12003153). Quantification of band intensity was performed using Fiji/ImageJ [37]. The relative levels of target proteins in the immunoblots were expressed as “fold change” over the EdU pulse WT sample, as follows: the target (DHX36) band intensity for the “no click” condition for each cell line was subtracted as background from the intensity values for all other conditions. The background corrected intensity for DHX36 in each sample was divided by the histone H3 intensity in that sample as a control for protein loading. The value for the EdU pulse sample from parental cells was used to normalize all other samples. Values for each condition were expressed as fold change in DHX36 compared to the WT EdU sample. Experiments were performed in triplicate, and relative fold change values were expressed as mean (± SD; *n* = 3).

### Chromatin fractionation and immunoblotting of 293FT and *REV1*^KO^ cells

293 FT cells (parental and *REV1*^KO^) were cultured and expanded to obtain 1 × 10^8^ cells in 150 mm dishes similarly, as described earlier for iPOND. Both cell lines were subjected to treatment with PDS for 15 min or 60 min. One dish was left as untreated control. At the end of treatment, cells were harvested by scraping, and cell pellets were washed twice with 1× PBS. Washed pellets were then lysed in solution A [10 mM HEPES pH 7.5, 10 mM KCl, 1.5 mM MgCl_2_, 0.34 M sucrose, 10% (v/v) glycerol, 0.1% (w/v) Triton X-100, 1 mM DTT, and phosphatase and protease inhibitors]. The lysed samples were pelleted at 1300 × *g* at 4°C for 10 min, and the supernatant was collected as cytosolic fraction. The pellet, containing the nuclei was washed with solution A and then resuspended in solution B (3 mM EDTA, 0.2 mM EGTA, 1 mM DTT, phosphatase and protease inhibitors) to lyse the nuclei. The samples were then pelleted at 1700 × *g* at 4°C for 10 min, and the supernatant was collected as nuclear soluble fraction. The pellet, containing the chromatin fraction, was washed once with solution B and then resuspended in the same solution B, supplemented with 200 mM NaCl in order to solubilize the chromatin fraction. After protein estimation by BCA assay for all the fractions, equal amounts of total protein from each sample were loaded, separated on SDS–PAGE, and immunoblotted to detect DHX36, REV1, PCNA, and histone H3 proteins in the samples.

### *In situ* protein interaction with nascent DNA replication forks (SIRF)

The SIRF experiments were performed largely as described [38, 39]. The parental 293 FT and *REV1*^KO^ (6D6) were plated on coverslips coated with 0.01% (w/v) poly-L-lysine (Millipore Sigma, #A-005-C) in six-well plates and allowed to attach overnight. Two independent biological replicates were performed (three technical replicates each time). Cells were treated with the following four conditions: 0.1% (v/v) DMSO carrier (No EdU) as a negative control, 125 μM EdU 15 min, 125 μM EdU 15 min with 10 μM PDS 15 min, 125 μM EdU 15 min with 10 μM PDS 60 min. Cells were washed twice with cold 1× PBS and fixed with 2% (v/v) formaldehyde in 1× PBS for 15 min in the dark at room temperature. The coverslips were washed three times with 1× PBS and stored up to one week in 1× PBS at 4°C.

Cells were permeabilized with 0.25% Triton X-100 in 1× PBS for 15 min at room temperature in the dark and washed twice with 1× PBS before being incubated in 100 μl Click-iT reaction solution containing 9 μM biotin azide, 1 μM AlexaFluor™ 488 azide, 100 mM sodium ascorbate, and 2 mM copper sulfate for 1 h at room temperature in humidity chambers. Coverslips were protected from light for all subsequent steps. Cells were then washed three times with 1× PBS and blocked with Duolink Blocking Buffer (Sigma–Aldrich, #DUO82007) for 1 h at room temperature. Primary antibodies targeting biotin (Sigma Millipore, #B7653) at 1:100 dilution and DHX36 (Abcam, #ab70269) at 1:200 dilution were incubated overnight at 4°C. The remaining steps involved proximity ligation technology and were performed according to manufacturer instructions using the PLA probes Duolink anti-Rabbit MINUS (Sigma Millipore, #DUO92005), Duolink anti-Mouse PLUS (Sigma Millipore, # DUO92001), and the Duolink in Situ Detection Reagents FarRed (Sigma Millipore, #DUO92013). Images were captured using an Olympus IX81 confocal microscope with a 60× PLAPON60XO objective using FV10-ASW 4.02 software. DHX36 SIRF foci within the nuclei were quantified using CellProfiler software version 4.2.8 (www.Cellprofiler.org) with >187 nuclei scored for each condition in each biological replicate. Combined nuclei scored was greater than 500 for both biological replicates. To identify replicating cells in EdU (+) conditions, only nuclei containing EdU signal greater than or equal to the mean plus two standard deviations of the EdU signal in their respective no-EdU conditions were analyzed. These were plotted with Prism-GraphPad version 11.0.0 (GraphPad Software, San Diego, California, USA, www.graphpad.com). Statistical significance was assessed using a Kruskal–Wallis test followed by Dunn’s multiple comparison.

### Proximity ligation assay

Cells were plated on coverslips coated with 0.01% (w/v) poly-L-lysine (Sigma Millipore, #A-005-C) in six-well plates and allowed to attach overnight. Cells were treated with PDS (10 μM) for the indicated amount of time (15, 60, and 240 min). For 293 FT cells, EdU was added concurrent with or in advance of PDS treatment (10 μM, 2 h). Cells were pre-extracted with 0.5% (w/v) Triton-X-100 in 1× PBS for 5 min at 4°C. The cells were fixed with 3.7% (v/v) formaldehyde in 1× PBS for 25 min at room temperature. The coverslips were washed three times with 1× PBS and stored up to one week in 1× PBS at 4°C. The proximity ligation assay (PLA) was performed according to manufacturer instructions using the Duolink Proximity Ligation Assay Kit (Sigma Millipore, #DUO92008). Primary antibodies targeting REV1 (Santa Cruz, #sc393022) at 1:25 dilution and hDHX36 (Abcam, #ab70269) at 1:50 were incubated for 1 h at 37°C. Coverslips were incubated with each primary antibody alone and no primary antibodies as negative controls. Images were captured using an Olympus IX81 confocal microscope with a 60× PLAPON60XO objective using FV10-ASW 4.02 software. Slides were kept in a slide box on ice until imaging to preserve the fluorescence intensity, which faded if slides warmed to room temperature. PLA foci were quantified by manually counting foci in 50 cells per condition (25 EdU positive and 25 EdU negative) and plotted with Prism-GraphPad version 9.5.1 (GraphPad Software, San Diego, California, USA, www.graphpad.com). Statistical significance was assessed using an unpaired Student’s t-test. Experiments were performed in at least biological triplicates in both parental 293 FT cells and in U2OS cells. Statistical significance was assessed using a Kruskal–Wallis test followed by Dunn’s multiple comparison.

### Treatment of 293FT and *REV1*^KO^ cells with inhibitors of ATR and ATM kinases

293 FT cells (parental and *REV1*^KO^) were cultured on 10 cm dishes to 50% confluency. The cells were then treated with inhibitors of ATR kinase [ATRi] (Ceralasertib AZD6738; MedChemExpress) or ATM kinase [ATMi] (AZD1390; ApexBio). Concentrations of inhibitors used were ATRi at 1 μM and ATMi at 25 nM. Cells were treated either with each inhibitor alone, or concurrently with 10 μM PDS for 15 min or 60 min. Cells treated with DMSO in separate dishes were used as negative controls. At the end of treatment, cells were harvested by scraping and processed to obtain cytosolic, nuclear-soluble, and chromatin-bound fractions. After protein estimation by BCA assay for all the fractions, equal amounts of total protein of each sample (for each fraction) were loaded and separated on SDS–PAGE and immunoblotted to detect DHX36, phosphorylated CHK1 (S296), and phosphorylated CHK2 (T68) as well as total CHK1 and CHK2, and either histone H3 or GAPDH. Experiments were performed in biological duplicates.

### Streptavidin pull-down of REV1-interacting proteins

The 293 FT cells engineered to express SFB-tagged REV1 proteins (WT, EY/AA, or ΔCTD) were cultured to a density of 2 million cells in 10-cm dishes for 24 h in DMEM complete medium. REV1 protein expression was induced by the addition of 0.2 μg/ml (v/v) doxycycline to the cells for 24 h, and the cells were treated with 10 μM PDS for 1 h. A separate dish for each cell line was also cultured in the absence of PDS as control. The cells were harvested and lysed as described earlier. After protein estimation by the BCA assay, 200 μg of total protein of each lysate was mixed with 100 μl streptavidin-agarose beads. The beads were pre-washed with the lysis buffer before mixing with lysates. The mixture was kept tumbling overnight at 4°C to allow the SFB-tagged REV1 proteins to bind to the beads. The following day, the beads were pelleted (4000 × *g*, 15 min at 4°C). The beads were washed twice with 400 μl PBS buffer. The washed beads were then mixed with 40 μl of Laemmli buffer and these samples were heated at 95°C for 10 min. Subsequently, immunoblotting was performed as described earlier, where the blots were probed for DHX36, Rev7, and PCNA.

### Expression and purification of DHX36

The human DHX36 protein sequence was analyzed using PrDOS (https://prdos.hgc.jp/) for determining regions of internal disorder. Further, a protein sequence alignment was performed between the human and bovine DHX36. The bovine sequence used for the alignment was from the published crystal structure (PDB 5VHE). Based on these analyses, a human DHX36 construct was designed to exclude the N-terminal amino acid residues 1–52 (Supplementary Fig. S3). This truncated DHX36 construct (aa 53–1008) with the “wild-type” (WT) sequence was cloned as an N-terminal tandem 6×His-GST-tagged fusion product in the pET28a vector. A mutant DHX36 construct (F912A/F913A or FF/AA) was generated by performing PCR-based site-directed mutagenesis on the WT construct to mutate the putative REV1-interacting region (RIR) motif residues (F912 and F913) to alanine. Mutations were confirmed by Sanger sequencing of the plasmid DNA. The DHX36 constructs (WT and FF/AA) were transformed into *E. coli* BL21 (DE3) competent cells for overexpression. Protein expression was induced with 0.5 mM IPTG at 18°C for 16 h. Cells were pelleted and lysed in 50 mM Tris–Cl (pH 7.5) buffer containing 300 mM NaCl, 10% (v/v) glycerol, 5 mM 2-mercaptoethanol (2-ME), and protease inhibitors (ThermoFisher Pierce). Lysate was clarified by centrifugation at 4°C at high speed (30 000 × *g*, 1 h), followed by filtration through 0.2 μm membrane filter. Clarified lysate was then applied to a HisTrap Ni-NTA affinity column on an FPLC, and the bound DHX36 protein was eluted in buffer described above, containing 0.4 M imidazole. The affinity-enriched DHX36 protein was then dialyzed into a final storage buffer containing 25 mM HEPES (pH 7.5), 200 mM NaCl, 10% (v/v) glycerol, and 5 mM 2-ME. Aliquots of both the WT and FF/AA DHX36 proteins were run on a SDS–PAGE denaturing gel and were stained with Coomassie stain to visualize bands (Supplementary Fig. S3). Aliquots were also subjected to immunoblotting and the blot was probed with anti-DHX36 antibody (Abcam) to confirm protein identity. The enriched DHX36 protein was then concentrated using a centrifugal spin concentrator (Amicon, 10 kDa MWCO), and the total protein concentration was determined using the BCA assay as described above. DHX36 protein concentration was calculated by correcting for percent purity and then stored at −80°C till further use.

### Glutathione-Sepharose pull-down assay

The WT or FF/AA DHX36 proteins, enriched as described earlier, were immobilized by mixing with Sepharose beads that were covalently crosslinked with reduced glutathione (GSH-Sepharose; Cytiva). Binding of the DHX36 proteins to the GSH beads was confirmed by western blot probed with anti-DHX36 antibody. Simultaneously, whole cell lysates were prepared from 293 FT cells induced to express the three SFB-Rev1 proteins (WT, EY/AA, or ΔCTD). After protein estimation of the lysates using BCA assay, 200 μg of total protein of each 293 FT lysate was mixed with 25 μl GSH-Sepharose beads (with WT or FF/AA DHX36 immobilized) from the previous step. As a negative control, aliquots of the three 293 FT lysates were also mixed with unbound 25 μl GSH-Sepharose beads (no DHX36 protein). Separately, similar pulldown reactions were performed coupled with endonuclease treatment, where 200 μg of total protein lysate from the 293 FT Flp-In cells were first supplemented by addition of 50 units of Pierce universal nuclease (ThermoFisher #88700) and then mixed with GSH-Sepharose beads. The bead-containing samples were kept tumbling overnight at 4°C to allow the SFB-tagged Rev1 proteins to bind to the DHX36-immobilized GSH beads. The following day, the beads were pelleted (4000 × *g*, 15 min at 4°C) and washed twice with 400 μl PBS buffer. The washed beads were then mixed with 40 μl of Laemmli buffer and these samples were heated at 95°C for 10 min to elute bound proteins. The proteins from the pull-downs were separated by SDS–PAGE. Immunoblotting was performed by probing for the pulled down SFB-Rev1 proteins using the anti-FLAG primary antibody.

### Expression and purification of REV1 CTD

The REV1 CTD construct (aa 1157–1251) was cloned as an N-terminal 6×His-tagged fusion product in the pET28a vector and overexpressed in *E. coli* BL21 (DE3) cells. Protein expression was induced with 0.5 mM IPTG at 18°C for 16 h. Cells were pelleted, lysed in 50 mM Tris–Cl (pH 7.5) buffer containing 300 mM NaCl, 10% (v/v) glycerol, 5 mM 2-mercaptoethanol (2-ME), and protease inhibitors (ThermoFisher Pierce). Lysate was clarified by centrifugation at 4°C at high speed (30 000 × *g*, 1 h), followed by filtration through 0.2 μm membrane filter. Clarified lysate was then applied to a HisTrap Ni-NTA affinity column on an FPLC, and the bound hREV1-CTD protein was eluted in buffer described earlier, containing 0.4 M imidazole. After confirming purity and yield by running an aliquot on an SDS–PAGE gel, the affinity-purified sample was further purified using a Superdex-75 size exclusion column in 25 mM HEPES (pH 7.5) buffer containing 200 mM NaCl, 10% (v/v) glycerol, and 5 mM 2-ME. Purity was estimated to be >90% based on Coomassie staining of a polyacrylamide gel (Supplementary Fig. S4). Protein concentration was determined using a BCA colorimetric assay (Thermo-Fisher Pierce). The purified protein was aliquoted and stored in 25 mM HEPES (pH 7.5) buffer containing 200 mM NaCl, 30% (v/v) glycerol, and 5 mM 2-ME at −80°C till further use.

### Fluorescence anisotropy

The binding affinity of the purified REV1 CTD protein for synthetic peptides corresponding to the RIR motifs from various proteins was measured using a plate-reader-based fluorescence polarization assay. Peptides corresponding to and flanking the consensus RIR motif in pol η and pol κ, as well as the human FANCJ helicase and the putative RIR motif from human DHX36, were synthesized with an N-terminal 6-Fluorescein (FAM) label (Genscript). Equilibrium binding reactions were set up in a 96-well plate, in 40 mM HEPES (pH 7.5) buffer containing 50 mM NaCl, 0.1 mg/ml bovine serum albumin, 5% (v/v) PEG-8000, and 2 mM 2-ME. Peptide concentration was kept constant at 1 µM in each well, while the protein concentration was titrated from 0 to 300 µM. After mixing all the reactants with a multi-channel pipette, the 96-well plate was incubated in the dark at room temperature for 10 min before reading the fluorescence polarization. The measured polarization values were plotted against protein concentration and fit to a quadratic equation to calculate the equilibrium dissociation constant (*K*_d_) of the protein for each peptide. All experiments were performed in triplicate and results were reported as the mean (± std. dev.).

### Biolayer interferometry

DHX36-derived peptide (GSGSGSPYCLLFFGGDISIQKDND) was synthesized by Genscript (Piscataway, NJ, USA) with a biotin modification at the N-terminus. The lyophilized powder was dissolved in Buffer G [20 mM HEPES pH 7.5, 150 mM KCl, 5 mM TCEP, and 5% (v/v) glycerol], and the peptide concentration was determined on a NanoDrop One spectrophotometer using a molar extinction coefficient of 1490 M^−1^cm^−1^. Biolayer interferometry (BLI) assays were performed as described [40] in Buffer G on a BLItz instrument (Sartorius) that was maintained at 25°C. High-precision streptavidin-coated SAX biosensors (Sartorius) were hydrated in Buffer G for 10 min before use. The biotinylated DHX36-RIR (300 nM) was loaded onto the SAX sensors for 120 s to enable binding of the peptide. The association and dissociation of REV1 CTD were monitored as a function of protein concentration (0.06–64 µM). All BLI measurements were performed in triplicate with freshly prepared samples to assess reproducibility of the results.

BLI binding isotherms were generated by plotting the steady-state plateau values of the association phases as a function of REV1 CTD concentration. The curves were fit to a 1:1 interaction model by using the Scientist 3.0 software to solve the implicit equations below, where Δλ was the observed BLI signal change, A was the amplitude, and K was the equilibrium association constant. R_f_ and R_t_ described the free and total concentration of the REV1 CTD. D_f_ and D_t_ were the free and total concentration of DHX36-RIR. The equilibrium constant value reported was determined from the average and 95% confidence interval of three independent data sets.


\begin{eqnarray*}
\Delta \lambda = A\left( {K{{{\mathrm{R}}}_f}/1 + K{{{\mathrm{R}}}_f}} \right)
\end{eqnarray*}

\begin{eqnarray*}
{{{\mathrm{D}}}_t} = {{{\mathrm{D}}}_f}\left( {1 + K{{{\mathrm{R}}}_f}} \right)
\end{eqnarray*}

\begin{eqnarray*}
{{{\mathrm{R}}}_t} = {{{\mathrm{R}}}_f}\left( {1 + K{{D}_f}} \right).
\end{eqnarray*}

## Results

### Aberrant Fork elongation accompanied REV1 loss with ssDNA gaps persisting when G4 is stabilized

**Figure 1.**
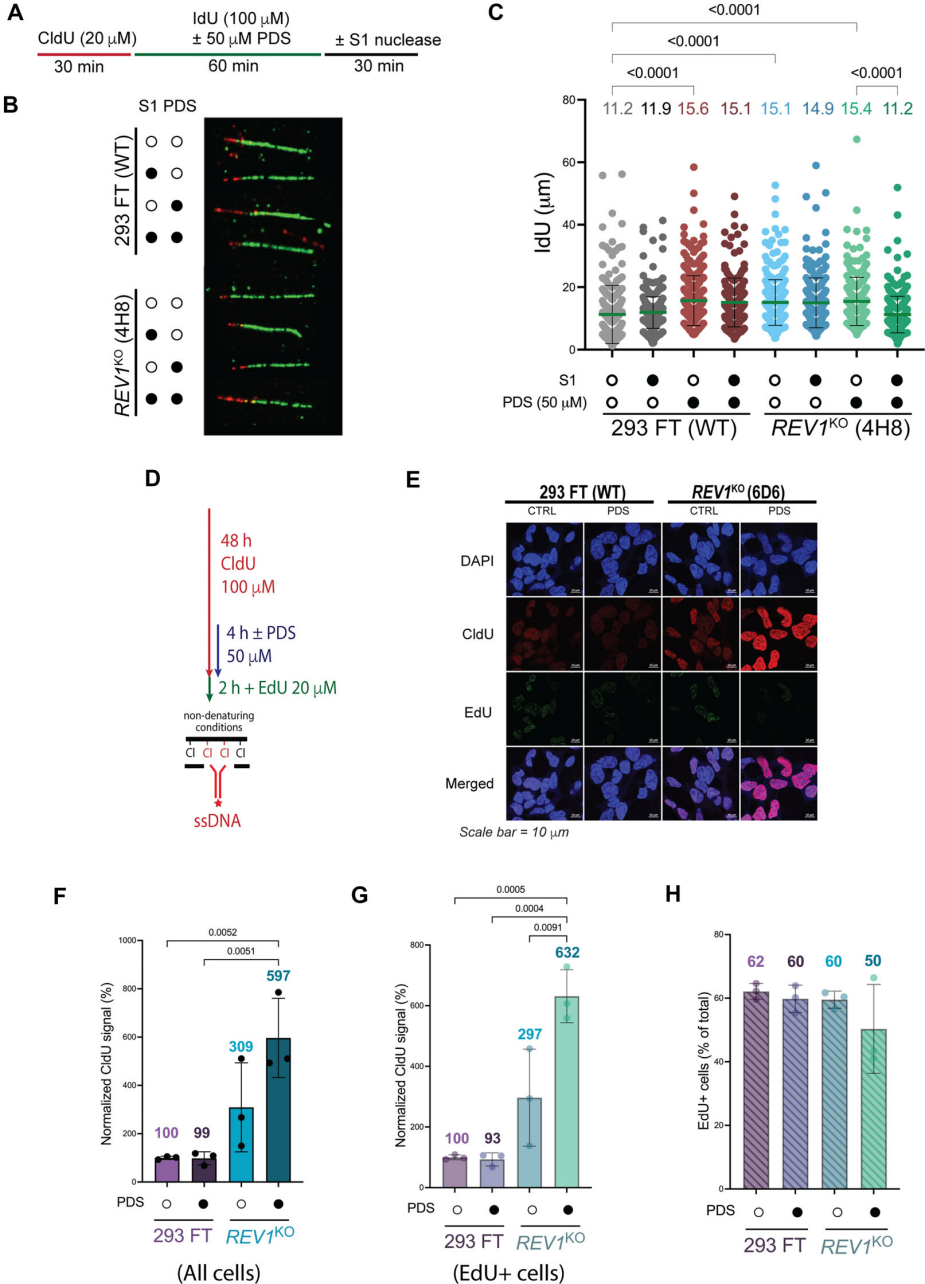
REV1 promotes continuous DNA replication and limits persistent ssDNA in response to G4 stabilization. (**A**) A schematic illustration of the experimental design is shown for the DNA fiber spreading experiments. Experiments were performed using both 293 FT parental and *REV1*^KO^ (4H8) cells (± S1 nuclease and ± PDS). PDS treatment was concurrent with the 60-min IdU pulse. (**B**) Representative images of DNA fibers from 293 FT parental and *REV1*^KO^ (4H8) cells (± S1-nuclease and ± PDS) are shown. Fiber track lengths for both the CldU and IdU labeled tracks were measured for all samples. (**C**) Quantification of IdU track lengths is shown for 293 FT parental and *REV1*^KO^ (4H8) cells (± S1 nuclease and ± PDS). Results from two independent biological replicates are shown. The mean fiber length for each condition is noted above the data, where >195 fibers were scored per condition. Statistical significance was evaluated by performing a Kruskal–Wallis test followed by a Dunn’s multiple comparisons post hoc test. Selected *P*-values most relevant to interpretation of the results are shown. (**D**) A schematic diagram of the treatments used in the CldU (ssDNA) assay is shown with a cartoon denoting that imaging is done under native or non-denaturing conditions. Signal from the anti-BrdU antibody should only arise from CldU in ssDNA. (**E**) Representative images for immunofluorescent detection of CldU (ssDNA), EdU, and DAPI nuclear staining are shown for parental WT and *REV1*^KO^ (6D6) cells treated with either vehicle or PDS (50 µM, 4 h). CldU signal was quantified using CellProfiler and CldU intensity normalized to the average intensity for untreated parental WT 293 FT cells. Three replicates were performed with >1800 cells scored per condition. The mean (± std. dev.) for each replicate is shown for (**F**) all cells and (**G**) EdU-positive cells. The percentage of cells with detectable EdU signal was also measured and reported as a percentage of the total number of cells imaged in panel (**H**). *P*-values were calculated using a one-way ANOVA with a Tukey multiple-comparisons test. Only *P*-values ≤.05 are noted. The mean CldU intensity is noted above the data for each condition.

We sought to improve our mechanistic understanding of how REV1 influences G4 replication dynamics in mammalian cells. To directly assess the influence of REV1 on fork progression and ssDNA formation in cells experiencing G4 stress, we performed DFS experiments (± S1 nuclease) in parental (WT) and *REV1*^KO^ 293 FT cells ± 50 μM PDS in the second (IdU) labeling step (Fig. 1A). The inclusion of S1 nuclease treatments allowed us to measure relative changes in ssDNA levels on regions of newly synthesized DNA. The *REV1*^KO^ cells were generated using CRISPR–Cas9 technology, as described in the “Materials and methods” section. Two “knock-out” clones were isolated (4H8 and 6D6; Supplementary Fig. S1). We used both the 4H8 and 6D6 cell lines throughout the study with similar results being obtained for each of the *REV1*^KO^ 293 FT clones.

The DFS results for vehicle-treated control 293 FT cells behaved as expected. The IdU track lengths in parental (WT, REV1-proficient) 293 FT cells were around 11 μm in length following a 1 h pulse with IdU, and they were not shortened by S1 nuclease treatment (Fig. 1B and C). Treating parental 293 FT cells with PDS produced longer IdU tracks (i.e. unrestrained or aberrant fork elongation) relative to vehicle-treated cells (Fig. 1B and C). The S1 nuclease treatment did not shorten the IdU tracks in PDS-treated cells (Fig. 1B and C), indicative of the fact that damage tolerance mechanisms in the parental cell line were able to limit the formation of persistent ssDNA in the wake of G4 stabilization.

Fiber experiments in the *REV1*^KO^ (4H8) cells revealed a different story. Loss of REV1 resulted in longer IdU tracks but no apparent ssDNA gap formation in vehicle-treated cells (Fig. 1B and C). *REV1*^KO^ (4H8) cells exhibited longer IdU tracks when PDS was added to the experiment, and those longer tracks were shortened by S1 nuclease treatment (Fig. 1B and C), which contrasted with results for PDS-treated parental 293 FT cells. Thus, loss of REV1 resulted in aberrant fork elongation and ssDNA gap formation in cells challenged by G4 stabilization.

The formation of ssDNA gaps near sites of newly synthesized DNA in PDS-treated *REV1*^KO^ (4H8) cells was further supported by immunofluorescence microscopy experiments measuring CldU incorporation using non-denaturing conditions (Fig. 1D). In those assays, we compared the baseline CldU/ssDNA signal for parental WT and *REV1*^KO^ (6D6) cells to that observed when cells were exposed to treatment with PDS (Fig. 1E). An EdU pulse was added to the experiments to label cells with active DNA synthesis (i.e. EdU + cells). The measured baseline CldU/ssDNA intensity was higher in *REV1*^KO^ (6D6) cells compared with the parental cells (Fig. 1F), but the difference did not reach the threshold for statistical significance (*P*-value = .2429). The trends were largely similar in EdU + cells (Fig. 1G), suggestive of an effect that extends outside of active cell division.

Treatment with PDS (50 µM, 4 h) did not change the CldU/ssDNA signal in REV1-proficient parental cells, regardless of EdU status (Fig. 1F and G). The CldU/ssDNA signal increased six-fold in PDS-treated *REV1*^KO^ (6D6) 293 FT cells (Fig. 1F and G), again consistent with G4 stabilization producing higher levels of ssDNA in REV1-deficient cells. The percentage of cells with detectable EdU signal was not significantly changed by the 4 h PDS treatment (Fig. 1H), suggesting that G4 stabilization did not have an immediate impact on proliferation. We did note a trend downward for the fraction of EdU + *REV1*^KO^ cells treated with PDS, suggestive of increased sensitivity to G4 stabilization compared to parental 293 FT cells (Fig. 1H). Taken together, the DFS and CldU/ssDNA results are consistent with the idea that loss of REV1 results in defective replication gap suppression (RGS) in cells treated with PDS.

### The accuracy of replication across a G4 motif in the leading strand was more sensitive to REV1 loss than lagging strand synthesis

Previous studies had implicated REV1 in leading strand G4 bypass [27, 28, 41]. We reported a dramatic increase in mutagenic replication when a G4 site was placed in the lagging strand [29]. This increase in mutation frequency was further exacerbated by treatment with the G4 stabilizing agent PDS. We wondered if deletion of REV1 would have the same impact on replication fidelity when the G4 motif was placed in the leading strand?

**Figure 2.**
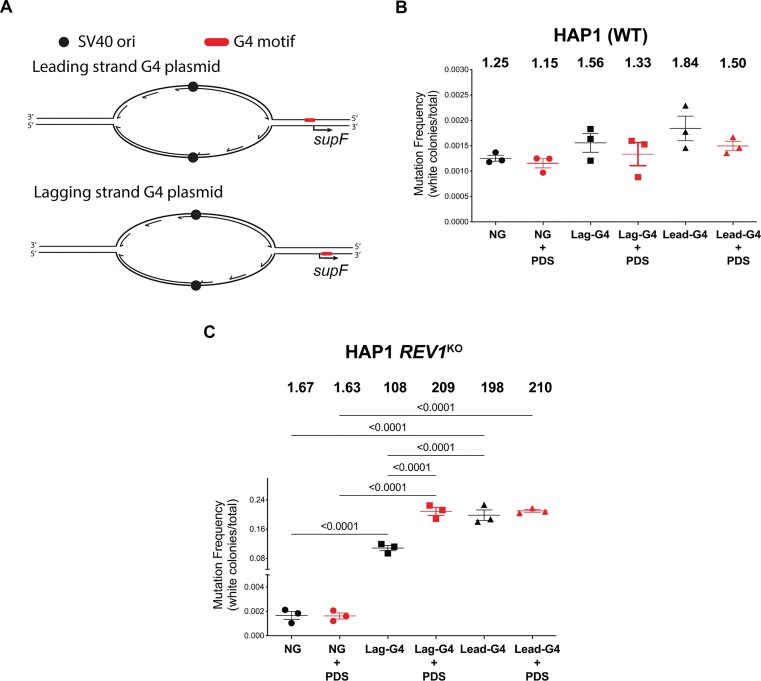
REV1 promotes accurate replication of G4 motifs in both leading and lagging strand DNA. (**A**) Schematic illustration of a G4 motif was positioned in the pSP189 plasmid to assess REV1-dependent effects on the accuracy of leading or lagging strand G4 bypass using the *supF* mutagenesis assay. The mutation frequency is shown for each of the plasmids replicated in the (**B**) parental (REV1-proficient) and (**C**) *REV1*^KO^ HAP1 cell lines. The HAP1 cells were transfected with one of three forms of the pSP189 plasmid: NG—non-G4 unmodified/control; Lag-G4—plasmid with Myc-G4 sequence inserted upstream of the *supF* gene on the lagging strand; Lead-G4—plasmid with Myc-G4 sequence inserted downstream of the *supF* gene on the leading strand. Experiments were performed either in the absence or presence of 10 μM PDS. The results for control (vehicle-treated) experiments are shown in black symbols. Those performed in the presence of PDS are shown as red symbols. The mean mutation frequency values (×10^3^) for each condition from three biological replicates are indicated above the data. Statistical significance was assessed using an ordinary one-way ANOVA with a multiple comparisons test. Selected *P*-values are shown for comparisons most relevant to interpretation of the results.

To answer this question, we conducted experiments using the *supF* shuttle-vector mutagenesis assay [42]. We measured mutation frequencies for the non-G4 (NG) control and two G4-containing plasmids—one with the *c-Myc*-derived G4 motif in the leading strand (Lead-G4) and the other *c-Myc*-derived G4 motif in the lagging strand (Lag-G4; Fig. 2A)—using both WT and *REV1*^KO^ HAP1 cells (±10 µM PDS). HAP1 cells were used because, in our hands, plasmids recovered from the 293 FT cell line did not produce an evenly spread field of colonies in the MBM7070 reporter strain. As a point of clarity, the *supF* experiments we reported previously with the NG control and G4 in the lagging strand were repeated for the current work to ensure reproducibility.

As expected, there was no meaningful change in mutation frequency for any of the three plasmids in the WT parental HAP1 cell line, and the addition of PDS did nothing to change that result (Fig. 2B). Moving into *REV1*^KO^ cells, the mutation frequency for the NG control plasmid was similar to that observed in the WT HAP1 cells—compare the mean values of 1.25 × 10^−3^ for WT parental cells with 1.67 × 10^−3^ for *REV1*^KO^ cells (Fig. 2C). Treatment with PDS had no effect on NG replication fidelity in *REV1*^KO^ HAP1 cells (Fig. 2C). By way of comparison, the mutagenesis results for G4 plasmids in *REV1*^KO^ HAP1 cells revealed an intriguing difference between leading and lagging strands.

The mutation frequency for the G4 motif in the lagging strand increased 65-fold over that observed for the NG control, and this increase was almost doubled when cells were exposed to PDS (Fig. 2C). These findings were comparable to our previous results monitoring REV1-dependent changes in mutagenesis of G4 in the lagging strand [29]. We were surprised to find that bypass of a leading strand G4 was almost twice as mutagenic as lagging strand G4 bypass in *REV1*^KO^ cells without any exogenous treatment, being elevated ∼120-fold over the NG control plasmid (Fig. 2C). Perhaps just as surprisingly, the addition of PDS did not further increase mutagenic replication of leading strand G4 motif (Fig. 2C). From these results, we concluded that loss of REV1 has a more direct impact on the fidelity of leading strand G4 bypass than lagging strand synthesis and that the effect on leading strand accuracy was insensitive to G4 stabilization.

### REV1-deficient cells have a more abundant nuclear G4 signal and have impaired proliferation in response to G4 stabilization

G4 formation is most abundant when ssDNA is formed during S-phase or in regions of chromatin that are less compact, such as actively transcribed regions of the genome [43, 44]. Moreover, REV1 interacts with G4 unwinders and is itself capable of disrupting G4 tetrads [28, 30]. Loss of REV1 leaves epigenetic scars where failed G4 bypass impairs maintenance of repressive histone marks [27]. Given the PDS-dependent increase in ssDNA signal and the apparent defect in RGS observed in REV1-deficient cells, we speculated that loss of REV1 could have an impact on the overall genomic G4 signal in mammalian cells. We, therefore, measured the nuclear G4 content by using immunofluorescence microscopy to monitor signal from the BG4 monoclonal antibody.

**Figure 3.**
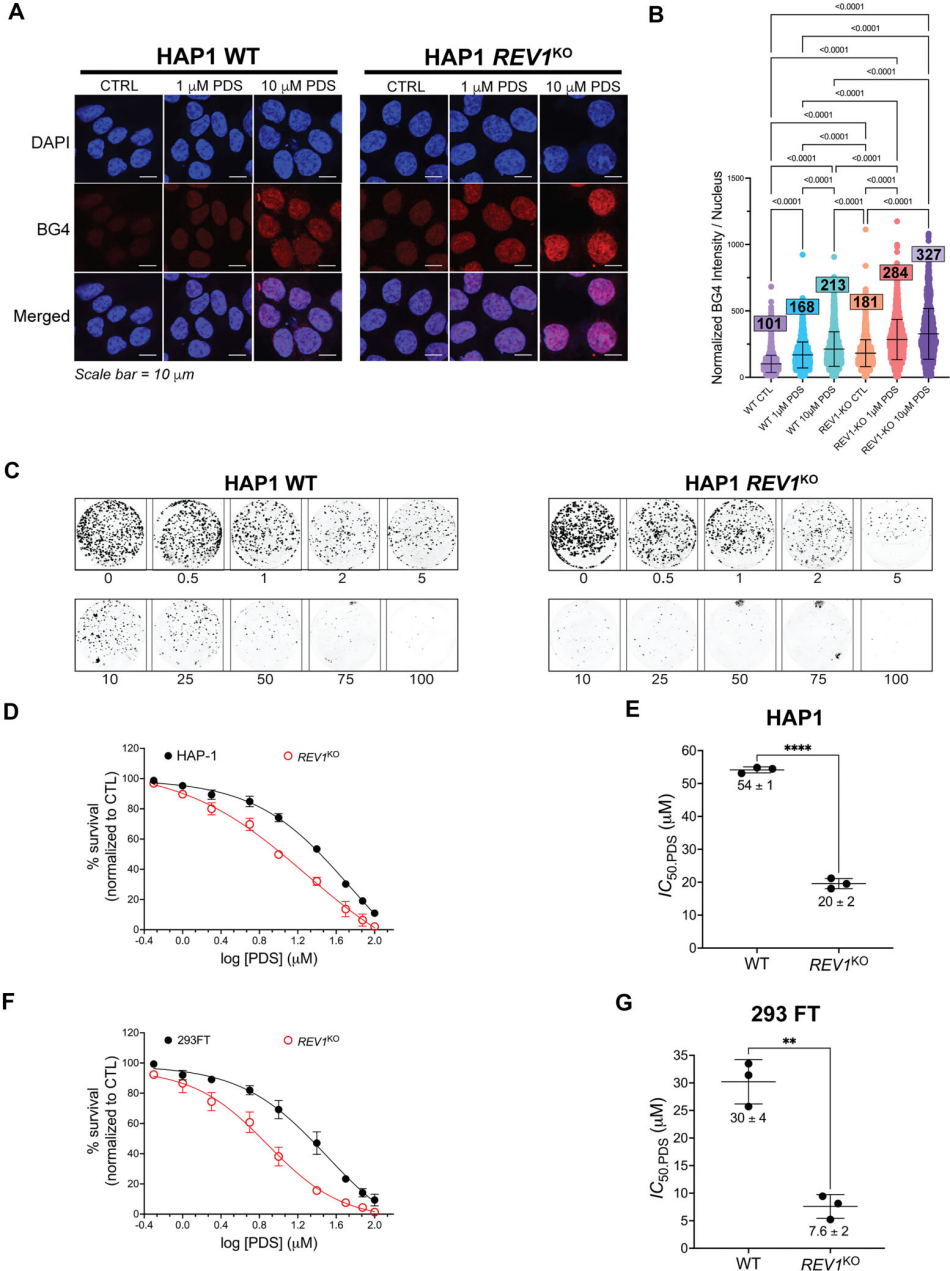
REV1 is a determinant of genomic G4 content and cell viability in response to G4 stabilization. (**A**) Immunofluorescence microscopy of BG4 signal was used to monitor REV1-dependent changes in genomic G4 content. Nuclear BG4 signal intensity was measured for untreated WT and *REV1*^KO^ HAP1 cells and for cells treated overnight with PDS (1 and 10 μM). BG4 signal intensity was normalized against that observed for untreated HAP1 WT cells. Representative images are shown for the DAPI-stained (blue), BG4-stained (red), and the “merged” for both cell types under each of the three conditions. (**B**) Nuclear BG4 signal intensity was quantified for experiments described in panel (A). Three independent biological replicates were performed (>1000 cells quantified per condition). Statistical significance was evaluated by performing a Kruskal–Wallis test followed by a Dunn’s multiple comparisons post hoc test. *P*-values <.05 are shown. (**C**) Clonogenic survival assay was performed on the parental and *REV1*^KO^ HAP1 cells to study the effect of G4 stabilizing agents. Representative images of colonies for both cell types treated with the G4 stabilizer pyridostatin (PDS) are shown, with the PDS concentration (0 to 100 μM) indicated below each condition. Resulting colonies were stained with Coomassie Brilliant Blue, and images of the colonies were recorded on an Odyssey CLx imager using the Image Studio 5.2 software. Colonies were counted using the colony-counting tool in the Fiji software. Assays were performed in three biological replicates. (**D**) Results from the clonogenic assays with parental and *REV1*^KO^ HAP1 cells described above with PDS for three biological replicates are shown. Percent survival was calculated for each condition, by normalizing to the control (untreated), taken to be 100%. Data were fit to a four-parameter log [PDS] versus response equation to obtain an estimate of the *IC*_50_ values. (**E**) The mean (± std. dev.) IC_50_ values are shown for three independent biological replicates for parental WT and *REV1*^KO^ HAP1 cells treated with PDS. Statistical significance was assessed by performing an unpaired Student’s t-test (**P < .01, ****P < .0001). (**F**) Results from the clonogenic assays with parental 293 FT and *REV1*^KO^ (6D6) cells treated with PDS for three biological replicates are shown. Percent survival was calculated for each condition, by normalizing to the control (untreated), taken to be 100%. Data were fit to a four-parameter log [PDS] versus response equation to obtain an estimate of the *IC*_50_ values. (**G**) The mean (± std. dev.) IC_50_ values are shown for three independent biological replicates for parental 293 FT and *REV1*^KO^ (6D6) cells treated with PDS. Statistical significance was assessed by performing an unpaired Student’s t-test (**P < .01, ****P < .0001).

There was an increase in nuclear BG4 signal for *REV1*^KO^ HAP1 cells compared with the parental (WT) HAP1 cells (Fig. 3A and B). We repeated these experiments in WT and *REV1*^KO^ 293 FT cells. The trends in BG4 signal for the 293 FT cell line were similar to those observed for HAP1 cells (Supplementary Fig. S5), although the baseline difference in BG4 signal between WT and *REV1*^KO^ 293 FT cells was not as large as that observed for HAP1 cells (compare BG4 signal for vehicle-treated *REV1*^KO^ cells in Fig. 3B with Supplementary Fig. S5B).

Exposing cells to PDS resulted in a dose-dependent increase in G4 signal for both parental (WT) and *REV1*^KO^ cells in both cell lines (Fig. 3A and B and Supplementary Fig. S5). For the parental HAP1 cells, treatment with 1 µM PDS for 24 h increased BG4 signal ∼70 percentage points above the untreated control cells and treatment with 10 µM PDS for 24 h increased BG4 signal >2-fold (Fig. 3B). Treating with 1 µM and 10 µM PDS increased BG4 signal another ∼1.5- and ∼1.8-fold, respectively, over that measured for untreated *REV1*^KO^ cells (Fig. 3B). Similar trends were observed in 293 FT cells (Supplementary Fig. S5). Overall, the general increase in BG4 signal for untreated REV1-deficient cells is consistent with dysregulated control of G4 formation in the genome and a sign that cells lacking this TLS enzyme face an elevated G4 burden. Proportionally, PDS increased BG4 signal in both parental and *REV1*^KO^ cells about the same, but in all cases there was more BG4 signal when REV1 was absent.

We reasoned that defects in DNA replication combined with the increased abundance of G4 signal in REV1-depleted cells could produce sensitivity to G4 stabilizing agents. This expectation was borne out in colony formation assays. Deletion of REV1 decreased the proliferative capacity of both HAP1 and 293 FT cells treated with PDS (Fig. 3C–G), indicative of a general effect that is not limited to one cell line. Furthermore, treatment with two other G4 ligands—PhenDC3 and NMM—resulted in reduced proliferative capacity of *REV1*^KO^ 293 FT cells compared to the parental counterparts (Supplementary Fig. S6), which is also consistent with a general role for REV1 in sustaining cell proliferation when G4s are stabilized.

### Loss of REV1 reduced the accumulation of DHX36 on chromatin surrounding nascent DNA

**Figure 4.**
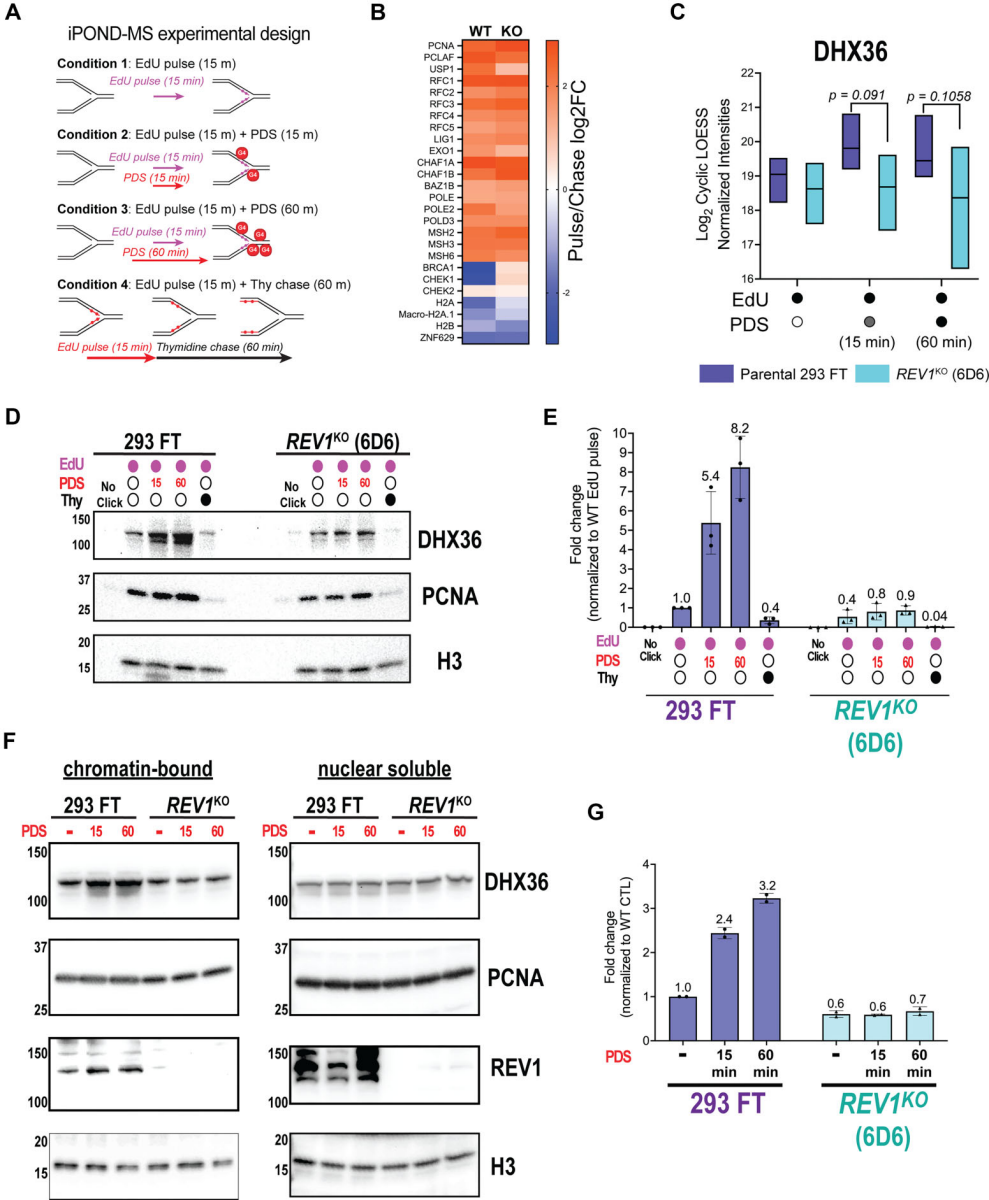
Proteomic analysis of replication proteins reveals DHX36 depletion at the fork in REV1-deficient cells treated with PDS. (**A**) A schematic illustration of the experimental design for the isolation of proteins on nascent DNA (iPOND-MS) experiments is shown. All four experimental conditions—EdU pulse (CTL), EdU pulse (+ PDS, 15 min), EdU pulse (+ PDS, 60 min), and thymidine chase—were performed in both WT and *REV1*^KO^ (6D6) 293 FT cells (*n* = 4 independent experiments). (**B**) A heat map replotting the log_2_ fold change (FC) in protein abundance is shown to illustrate the enrichment of DNA replication factors and depletion of histones observed in the EdU pulse samples relative to the thymidine chase samples for both parental (WT) and *REV1*^KO^ (6D6) cells. Data independent acquisition (DIA) was used to quantify the amount of individual proteins in each sample. The heat map range is from log_2_FC values of 2 (red) to −2 (blue). (**C**) Histogram showing the normalized (LOESS) intensities of DHX36 in the iPOND-MS for the parental 293 FT and *REV1*^KO^ (6D6) cells in the EdU pulse (± PDS) samples. Data are shown as mean (± std. dev.) from four independent replicates. Statistical significance (*P*-value) for the indicated pairs is shown. (**D**) DHX36 accumulation near sites of DNA synthesis was monitored in WT and *REV1*^KO^ cells by repeating the iPOND experiments followed by immunoblotting. Proteins captured on streptavidin beads for each condition were separated by SDS–PAGE and immunoblotting was performed for the target protein. Band positions of molecular weight markers are indicated to the left of each blot. Experiments were performed in biological triplicate, with representative images for capture samples shown for both cell lines (images for input samples shown in Supplementary Fig. S8). The experimental condition is indicated above each panel. The blots were also probed for PCNA and histone H3 as controls for fork enrichment and equal loading, respectively. Band intensity in each sample was measured using Fiji software. (**E**) Quantification of DHX36 enrichment by immunoblotting for iPOND samples in WT and *REV1*^KO^ cells was performed as described in the “Materials and methods” section. The mean (± std. dev.) is shown for three independent biological replicates. Mean values for fold change are indicated above each condition. (**F**) Chromatin fractionation was performed on 293FT and *REV1*^KO^ cells, as described in the “Materials and methods” section. Briefly, both cells were cultured and subjected to PDS treatment (10 μM for 15 and 60 min) identical to the iPOND experiment. Untreated cells were used as negative control. After harvesting cell pellets, lysis and chromatin fractionation were performed to separate the nuclear-soluble and chromatin-bound fractions. Equal total protein (50 μg each) for both the chromatin-bound (left panels), and the soluble nuclear fraction (right panels) for each sample was loaded on separate gels and immunoblotted for DHX36, PCNA, and endogenous REV1 as indicated. (**G**) Quantification of chromatin-bound DHX36 signal in 293FT and *REV1*^KO^ cells was performed. Briefly, the signal intensity of DHX36 and histone H3 was measured using ImageJ, and fold-change was calculated as follows: the intensity for DHX36 in each sample was divided by the histone H3 intensity in that sample. These values were normalized to the DHX36 signal from the untreated 293 FT DHX36 sample to calculate relative fold change. Results are shown for two independent replicates (mean ± std. dev.).

The changes in fork elongation and ssDNA gap formation led us to determine if loss of REV1 impacts the composition of the replisome when it encounters G4 stress. To pursue this line of thought, we used iPOND-MS to identify differences in replication proteins in WT and *REV1*^KO^ (6D6) cells (Fig. 4A). To evaluate differences related to G4-induced replication stress, cells were treated with PDS (10 μM) for 15 and 60 min. We chose these time points because a previous iPOND-MS study found that replication stress response factors began accumulating near sites of nascent DNA synthesis within a few minutes of treatment with hydroxyurea [45].

Mass spectrometry was performed by the IDeA National Resource for Quantitative Proteomics. The false discovery rate (FDR) adjusted *P*-values and fold-change in protein abundance were used to evaluate differences between samples (Supplementary Fig. S7 and Supplementary Table S1). A brief note on the data analysis workflow for iPOND-MS; we used label-free quantitative proteomics to measure differences in protein abundance for the two cell lines. When we sorted proteins identified by iPOND as being differentially abundant based on the adjusted *P*-value, there were not any obvious TLS or G4 proteins at the top of any of the comparisons. It was further down the list where we discovered changes like those described for DHX36 below. This reflects a limitation of using an unbiased approach like mass spectrometry—sometimes the most “significant” changes in protein abundance are difficult to understand, and manual curation of the resulting list of proteins is sometimes necessary.

We first assessed the enrichment of replication proteins by dividing protein abundance in the EdU pulse samples by that observed in the thymidine chase samples. As expected, samples from both parental and *REV1*^KO^ (6D6) cells were enriched for PCNA, the clamp loader (RFC 1–5), pol epsilon, and other components of the replisome (Fig. 4B). In this way, we gained confidence that the iPOND method was enriching for proteins at the fork. There were, by and large, few differences in the enrichment for core replisome factors between the two cell lines. However, there were some notable exceptions. For example, BRCA1 and Chk1 seemed to be slightly enriched near sites of EdU incorporation in the *REV1*^KO^ (6D6) cells, which was quite distinct from the depletion of these two stress/damage response factors in the WT cell line (Fig. 4B), indicative of ongoing replication stress in the REV1-deficient background.

We then compared protein abundance in EdU pulse samples for parental 293 FT and *REV1*^KO^ (6D6) cells. Analysis of the proteomics data revealed that loss of REV1 led to significant changes in 121 proteins without any kind of exogenous replication stressor (Supplementary Fig. S7A). To determine which changes in fork composition were related to G4 bypass, we ascertained whether treatment with the G4 stabilizer PDS had a differential impact on known DNA replication and repair proteins in parental 293 FT and *REV1*^KO^ (6D6) cells. Using two PDS-treatment timepoints (15 and 60 min) allowed us to look for time-dependent changes in specific proteins identified by iPOND-MS. The most significant changes induced by PDS did not reveal obvious changes in known G4-related proteins (Supplementary Fig. S7B and C). Indeed, some known REV1 interactors (e.g. pol ζ, FANCJ, and other TLS pols) were not identified at all in the mass spectrometry results and likely reflect a limitation in the iPOND technique. However, our attention was drawn to the fact that the DHX36 helicase was enriched at the fork in both PDS-treated samples for parental 293 FT cells but not in the *REV1*^KO^ cells (Fig. 4C). This result was especially interesting given that DHX36, a well-studied G4 unwinder, can directly influence progression of the replisome past G4 structures [46].

We validated the mass spectrometry results by repeating the iPOND experiments and blotting for DHX36 accumulation in WT and *REV1*^KO^ cells (Fig. 4D and E). We used PCNA as a measure of equal enrichment of fork components for each condition and histone H3 was used as a loading control. The “click” chemistry reaction was clearly required for capture of proteins near EdU-labeled DNA (compare No Click lanes in Fig. 4D to those from inputs shown in Supplementary Fig. S8). The immunoblotting results were consistent with the quantitative proteomics results in that DHX36 signal increased as a function of PDS treatment and time in parental 293 FT cells (Fig. 4D and E). The PDS-induced increase in DHX36 signal observed for WT cells was much more pronounced than what was observed for the *REV1*^KO^ cells, where DHX36 signal barely increased at all after PDS treatment (Fig. 4D and E).

To ensure that the iPOND results with DHX36 were not limited to the 293 FT cell line, we repeated the iPOND experiments in parental (WT) and *REV1*^KO^ HAP1 cells and performed immunoblotting to measure DHX36 enrichment near the fork. Consistent with results for the 293 FT cell line, deletion of REV1 in HAP1 cells strongly reduced the amount of DHX36 enriched by the iPOND technique (Supplementary Fig. S9). In both cell lines, the amount of DHX36 retained near the fork in *REV1*^KO^ cells is less than half that observed for REV1-proficient cells (Supplementary Figs S4D and E and S9), suggestive of diminished loading of DHX36 onto DNA prior to G4 stabilization.

To examine REV1-dependent changes in DHX36 localization further, we performed immunoblotting on chromatin fractions from parental and *REV1*^KO^ (6D6) cells with and without PDS treatment (Fig. 4F and G). In alignment with the iPOND results, there was more chromatin-bound DHX36 in parental 293FT cells compared to *REV1*^KO^ (6D6) cells, and this was especially obvious in response to PDS treatment (Fig. 4F and G). There was, however, no difference in the nuclear soluble fraction of DHX36 when comparing between parental and *REV1*^KO^ (6D6) 293 FT cells (Fig. 4F, blot on the right). We also probed the chromatin fraction and nuclear soluble fractions for REV1. The REV1 signal largely mirrored DHX36, increasing on chromatin but changing little in the nuclear soluble fraction following PDS treatment in parental 293 FT cells (Fig. 4F). These results lend support to the conclusion that loss of REV1 produced a defect in accumulation of DHX36 on chromatin near nascent-strand synthesis in human cells, one that is especially pronounced in cells treated with PDS.

We performed immunoblotting for other proteins identified by iPOND-MS as being altered by deletion of REV1 and PDS treatment (Supplementary Fig. S10). The immunoblotting results largely mirrored the trends observed in the mass spectrometry data. As an example, ATM signal was strongly elevated in the *REV1*^KO^ cells compared to the parental cell line regardless of whether PDS was added or not (Supplementary Fig. S10A). Likewise, the replication stress response (RSR) scaffold TOPBP1 was more abundant in all the samples from *REV1*^KO^ cells compared to the parental cell line (Supplementary Fig. S10D). We also probed iPOND input samples for markers of DNA damage and replication stress and discovered that two markers of DNA damage and replication stress—pChk1(S296) and γH2AX—were elevated in PDS-treated *REV1*^KO^ cells (Supplementary Fig. S10F). As is typical of proteomics results, many of the findings reported here will need to be further validated and explored in the future, but several, at least, are consistent with elevated DNA damage and replication stress response activity in REV1-deficient cells. For that reason, we decided to test whether inhibition of either ATM or ATR impacted DHX36 localization to chromatin.

**Figure 5.**
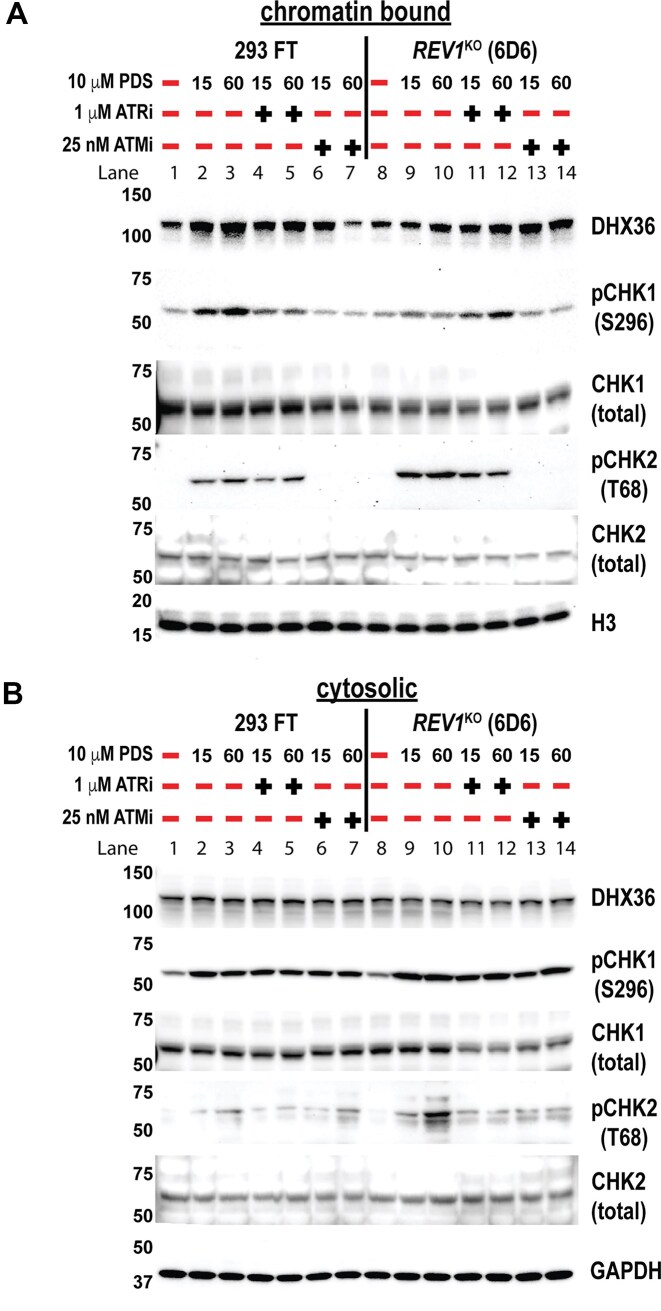
DNA damage signaling is elevated in PDS-treated REV1-deficient cells. Cells were treated with PDS alone or in combination with either the ATR inhibitor Ceralasertib (1 μM) or the ATM inhibitor AZD1390 (25 nM) for the indicated times. DMSO was used as a vehicle control. Immunoblotting for the indicated target proteins was performed on (**A**) chromatin and (**B**) cytosolic fractions. Lane numbers are noted for clarity.

We collected samples from parental and *REV1*^KO^ (6D6) cells (± 10 μM PDS for 15 and 60 min), separating chromatin-bound samples from soluble fractions. We then used immunoblotting to probe for DHX36 and two markers of ATM/ATR activation—pChk1 (S296) and pChk2 (T68). As before, DHX36 was more abundant on chromatin in the parental 293 FT cells compared to *REV1*^KO^ (6D6), and this was especially noticeable in samples from PDS-treated cells (Fig. 5A, compare lanes 1–3 with 8–10). Soluble DHX36 remained largely the same across treatment conditions in both cell lines (Fig. 5B, compare lanes 1–3 with 8–10). While there was more chromatin-bound pChk1 in the parental cell line after PDS treatment (Fig. 5A, lanes 1–3), it was apparent that PDS caused more sensing of DNA damage in the *REV1*^KO^ (6D6) cells; chromatin-bound pChk2 was elevated in the *REV1*^KO^ (6D6) cells (Fig. 5A, compare pChk2 signal in lanes 1–3 with 8–10), and there was more soluble pChk1 and pChk2 when compared to PDS-treated parental 293 FT cells (Fig. 5B, compare pChk1 and pChk2 signal for lanes 1–3 with 8–10), both of which indicate more severe genotoxic stress emanating from PDS-treated *REV1*^KO^ (6D6) cells.

Inhibition of ATR muted the accumulation of DHX36 in PDS-treated parental 293 FT cells (Fig. 5A, lanes 4–5), but ATM inhibition seemed to cause an even more pronounced defect in DHX36 binding to chromatin, especially in parental cells treated with PDS for 60 min (Fig. 5A, lane 7). Conversely, inhibiting either of the master kinases in *REV1*^KO^ (6D6) cells slightly increased the chromatin-bound DHX36 (lanes 11–14 on right side of Fig. 5A). We concluded from these experiments that ATM (and to a lesser extent ATR) was involved in retaining DHX36 on chromatin after prolonged G4 stress even in the presence of REV1.

### DHX36 accumulated distal from REV1 and the DNA synthesis machinery after prolonged PDS treatment

To further analyze REV1-mediated recruitment of DHX36, we performed SIRF experiments to monitor changes in DHX36 localization relative to sites of EdU incorporation. As with the iPOND and chromatin fractionation experiments, we treated parental and *REV1*^KO^ (6D6) cells with PDS (10 μM) for two time points (15 and 60 min). In this way, we could assess REV1- and PDS-related changes in DHX36 localization that occur within ∼40 nm of active DNA synthesis over time.

**Figure 6.**
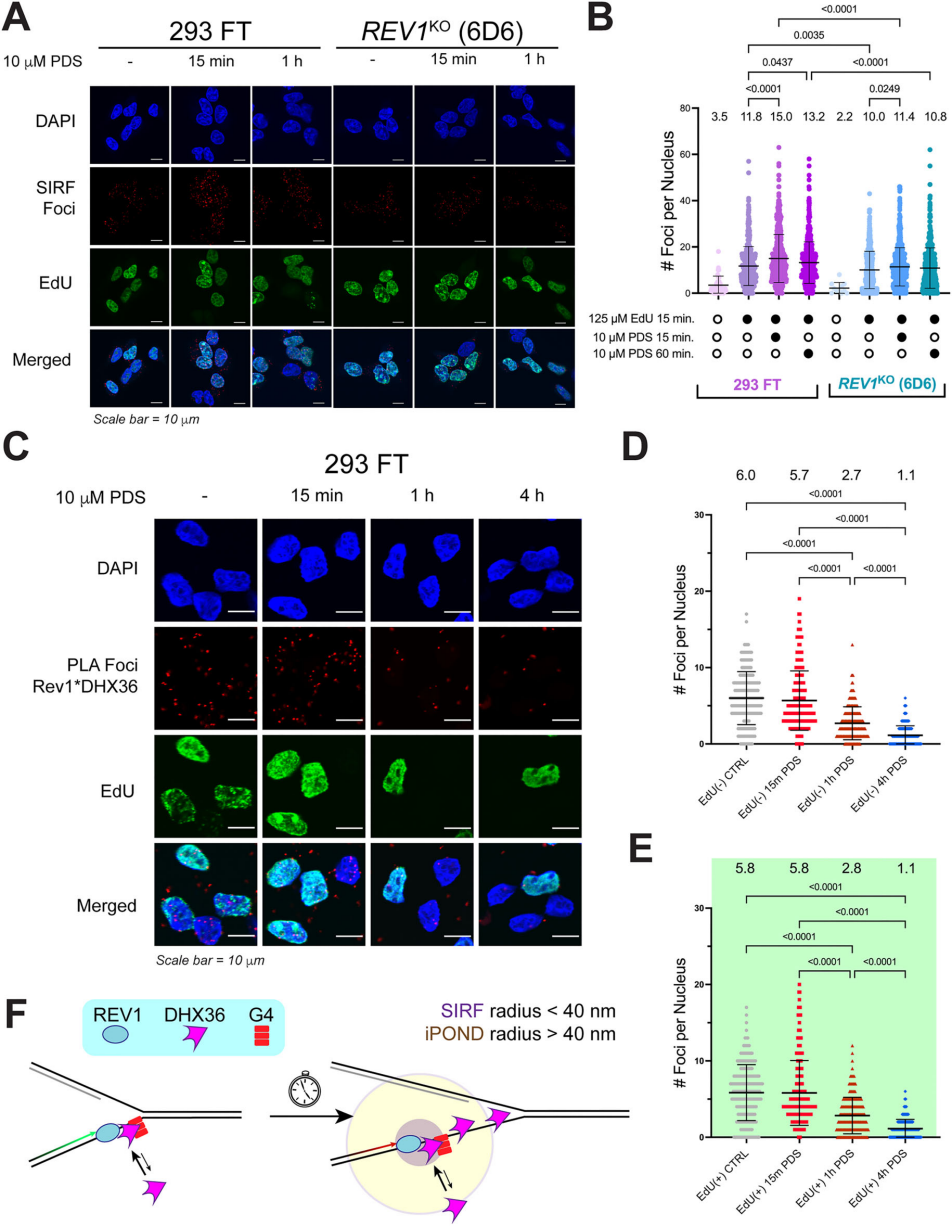
DHX36 accumulates at sites distal to REV1 and DNA synthesis machinery following prolonged treatment with PDS. (**A**) Representative images are shown for SIRF foci from parental and *REV1*^KO^ (6D6) 293 FT cells (± PDS, 10 μM, for the indicated time). Signals from DAPI stain (blue), DHX36-EdU (SIRF, red), AlexaFluor488 EdU (green), and the merged signal are shown. (**B**) Quantification of the number of DHX36-EdU SIRF foci per nucleus is shown for parental and *REV1*^KO^ (6D6) 293 FT cells. Results are from two independent biological replicates where >500 nuclei were scored per condition. The mean number of foci for each condition is noted above the data. Statistical significance was evaluated by performing a Kruskal–Wallis test followed by Dunn’s multiple comparison. *P*-values <.05 are shown. (**C**) Representative images are shown from experiments measuring REV1-DHX36 PLA in parental 293 FT cells. Signal from DAPI stain (blue), REV1-DHX36 (PLA, red), AlexaFluor488 EdU (green), and the merged signal are shown. Quantification of the number of REV1-DHX36 foci per nucleus is shown for (**D**) EdU(−) and (**E**) EdU + cells. Results are shown for three independent biological replicates with 50 nuclei scored per condition per replicate. The mean number of foci is shown above the data points. Statistical significance was evaluated by performing a Kruskal–Wallis test followed by Dunn’s multiple comparison. *P*-values <.05 are shown. (**F**) A cartoon model is shown depicting how changes in DHX36 signal relative to REV1 and EdU may change as a function of time and G4 stabilization, noting the different volume of space reported on by SIRF/PLA and the iPOND methods.

Consistent with iPOND and chromatin fractionation results, there were more DHX36-EdU SIRF foci in the parental cells than the *REV1*^KO^ (6D6) 293 FT cells (Fig. 6A and B). Treatment with PDS for 15 min increased DHX36-EdU SIRF foci in both parental and *REV1*^KO^ (6D6) 293 FT cells, with a slightly greater increase in the parental cell line (Fig. 6B). Contrary to our initial expectation, longer treatment time (60 min) did not increase DHX36-EdU SIRF foci in either cell line (Fig. 6B). This finding contrasted with the robust and time-dependent increase in DHX36 signal observed in the iPOND experiments (Supplementary Figs S4D and E and S9). A reasonable explanation of the differences between DHX36-related SIRF and iPOND results can be made by considering resolution (i.e. the volume of chromatin “space”) reported on by each technique. The iPOND technique can label several kilobases of DNA depending on EdU incubation time and replication rates inherent to the conditions [35, 47]. Interactions monitored by SIRF are limited to distances of no greater than 40 nm [38, 39]. Thus, DHX36 located beyond more than a few hundred base pairs from EdU may not be seen by SIRF. Also, SIRF relies on antibody recognition of proteins where the epitope may not be as accessible as in a denaturing gel. In any case, there were overall fewer DHX36-EdU SIRF foci in REV1-deficient cells, and PDS did not alter the number of SIRF foci much in either parental or *REV1*^KO^ (6D6) cells. We interpret these results to mean that DHX36 accumulates at a location distal (but not entirely remote) from sites of DNA synthesis after prolonged exposure to PDS.

We next performed a PLA to monitor how REV1 and DHX36 are oriented on chromatin in cells with and without PDS treatment. We pulsed the cells with EdU to distinguish between actively replicating cells (EdU+) and cells that were not proliferating (EdU−). Without any treatment, we observed good PLA signal in all 293 FT cells, regardless of whether they were EdU+ or EdU− (Fig. 6C–E). The number of REV1-DHX36 PLA foci per nucleus did not change after 15 min treatment with PDS (Fig. 6C–E). Longer treatment with PDS (1 and 4 h) resulted in diminished REV1-DHX36 foci in all cells (Fig. 6C–E). A similar reduction in the number of REV1-DHX36 foci was observed in U2OS cells treated with PDS for 1–4 h (Supplementary Fig. S11). Like the SIRF experiments, the REV1-DHX36 PLA results were suggestive to us of a scenario where prolonged G4 stabilization increased the amount of DHX36 accumulating at sites distal from REV1 and the DNA synthesis machinery (Fig. 6F).

### The CTD and the G4 selective properties of REV1 were necessary for optimal DHX36 recruitment to the fork

**Figure 7.**
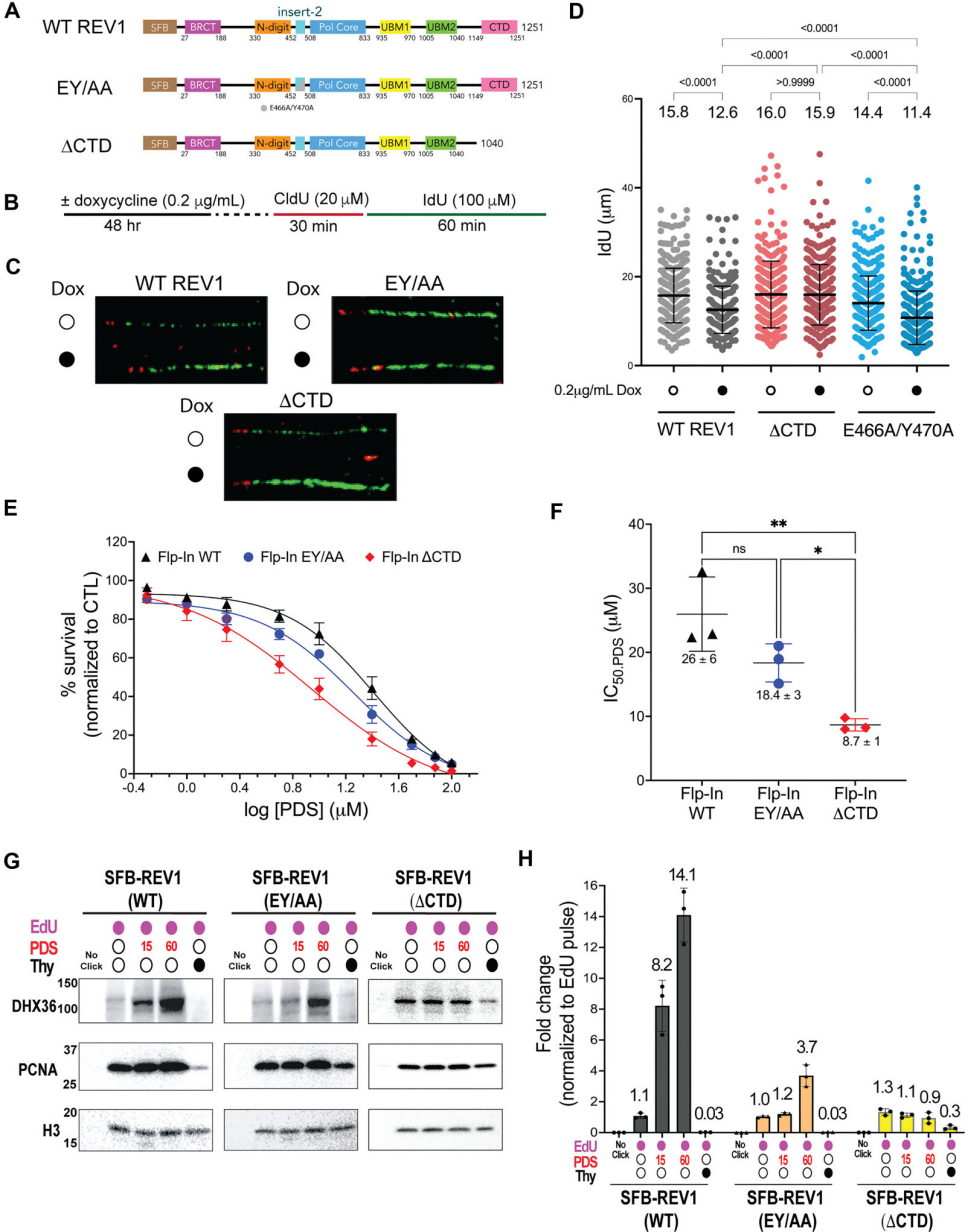
The REV1 CTD is essential for rescue of aberrant fork elongation, DHX36 recruitment to the fork, and tolerance of the anti-proliferative effects of PDS. (**A**) Schematic illustration of REV1 proteins “flipped” back into the 293 FT *REV1*^KO^ (6D6) for complementation assays: WT (aa 1–1251), E466A/Y470A (EY/AA), and the ΔCTD mutant (aa 1–1040). All three versions of REV1 were N-terminally tagged with SFB. (**B**) Schematic illustration of the DFS experimental scheme for Flp-In cell lines. (**C**) Representative fibers from Flp-In cell lines (± 0.2 μg/ml doxycycline). (**D**) Quantification of IdU track lengths is shown for each of the Flp-In cell lines (± 0.2 μg/ml doxycycline). Results from two independent biological replicates are shown. The mean fiber length for each condition is noted above the date, where >300 fibers were scored per condition. Statistical significance was evaluated by performing a Kruskal–Wallis test followed by a Dunn’s multiple comparison post hoc test. *P*-values most relevant to interpretation of the results are shown. (**E**) Clonogenic survival assay was performed on the three Flp-in cells, inducibly expressing the SFB-tagged REV1 proteins (WT, EY/AA, or ΔCTD), to study the effect of G4-stabilizing agent PDS, as described earlier. Percent survival was calculated for each condition, by normalizing to the control (untreated), taken to be 100%. Data were fit to a four-parameter log [PDS] versus response equation to obtain an estimate of the *IC*_50_ values. Experiments were performed in three biological replicates. (**F**) The mean *IC*_50_ values from three biological replicates for the clonogenic assay are shown along with the mean (± std. dev.). Percent survival was calculated for each condition, by normalizing to the control (untreated), taken to be 100%. Statistical significance was calculated by performing non-parametric t-tests assuming Gaussian distribution. (n.s. = non-significant; *P = .01, **P = .001). (**G**) iPOND experiments were performed in 293 FT cells expressing either the WT, (E466A/Y470A) EY/AA, or ΔCTD mutant versions of REV1. Expression of the REV1 protein was induced by the addition of doxycycline (0.2 μg/ml) for 48 h prior to iPOND. Three independent biological replicates were performed with representative images for the capture samples shown for both cell lines. PCNA and histone H3 were used as controls for fork enrichment and equal loading, respectively. (**H**) Quantification of DHX36 enrichment by immunoblotting is shown for iPOND samples from each of the 293 FT cell lines induced to express WT, (E466A/Y470A) EY/AA, or ΔCTD mutant versions of REV1. Results are expressed as fold change in DHX36 signal normalized to the “no click” lane. The mean (± std. dev.) is shown for three independent biological replicates. Mean values for fold change are indicated above each condition.

To characterize the features of REV1 required for accumulation of DHX36 at the fork in response to G4 stress, we re-introduced doxycycline-inducible WT and two mutant forms of REV1 into the *REV1*^KO^ 293 FT (6D6) cell line (Fig. 7A and Supplementary Fig. S2). We chose to use the EY mutant because we had previously shown that mutating residues E466 and Y470 to alanine diminished the ability of REV1 to selectively bind G4 DNA, while also abrogating the tetrad-disruptive capacity of REV1 [29, 31]. We also deleted the CTD of REV1 by introducing a stop codon after amino acid residue 1040 (Fig. 7A and Supplementary Fig. S2). In this way, we created separation-of-function REV1 mutants with defects in G4 selective properties (EY) and protein–protein interactions (ΔCTD), respectively.

DFS experiments were performed with the Flp-In cell lines, inducing expression of REV1 48 h prior to pulsing with thymidine analogues (Fig. 7B). Both WT REV1 and the EY mutant were able to suppress the aberrant fork elongation observed in the *REV1*^KO^ 293 FT (6D6) cell line (Fig. 7C and D). Only the REV1 ΔCTD was unable to restore wild-type fiber lengths (Fig. 7C and D). Similarly, clonogenic survival assays revealed that both WT and EY mutant REV1 were able to rescue sensitivity to PDS observed for the *REV1*^KO^ 293 FT (6D6) cell line, but the REV1 ΔCTD remained as sensitive to PDS as *REV1*^KO^ 293 FT (6D6) cells (Fig. 7E and F). We then went on to see if DHX36 localization to nascent DNA could be rescued in the Flp-In cell lines.

We performed iPOND in Flp-In 293 FT cell lines (± 10 μM PDS for 15 or 60 min). In line with our results to this point, complementation with WT REV1 fully restored accumulation of DHX36 to the fork in PDS-treated iPOND samples (Fig. 7G and H), recapitulating the results with the parental 293 FT cell line and strengthening the conclusion that WT REV1 is a determinant of DHX36 retention when the fork encounters G4-related blockage. We then determined if mutating part of the G4 interface (i.e. the EY mutant) or the C-terminal protein–protein interaction domain of REV1 (ΔCTD) impacted the accumulation of DHX36 near sites of nascent strand synthesis following exposure to PDS.

In contrast to WT enzyme, there was noticeably less robust retention of DHX36 at the fork when we complemented it with the EY mutant of REV1 (Fig. 7G and H). However, there was some accumulation of DHX36 in cells expressing the EY mutant after 60 min of PDS treatment. More dramatically, complementation with REV1 ΔCTD completely abrogated any build-up of DHX36 in response to PDS treatment (Fig. 7G and H). The summation of these findings supports the conclusion that the REV1 CTD is required to effectively retain DHX36 near sites of DNA synthesis in cells treated with a G4 stabilizer. They also imply that optimal recruitment of DHX36 occurs when REV1 can bind more tightly to G4 structures than unstructured DNA.

### DHX36 interacts directly with the REV1 C-terminal domain

**Figure 8.**
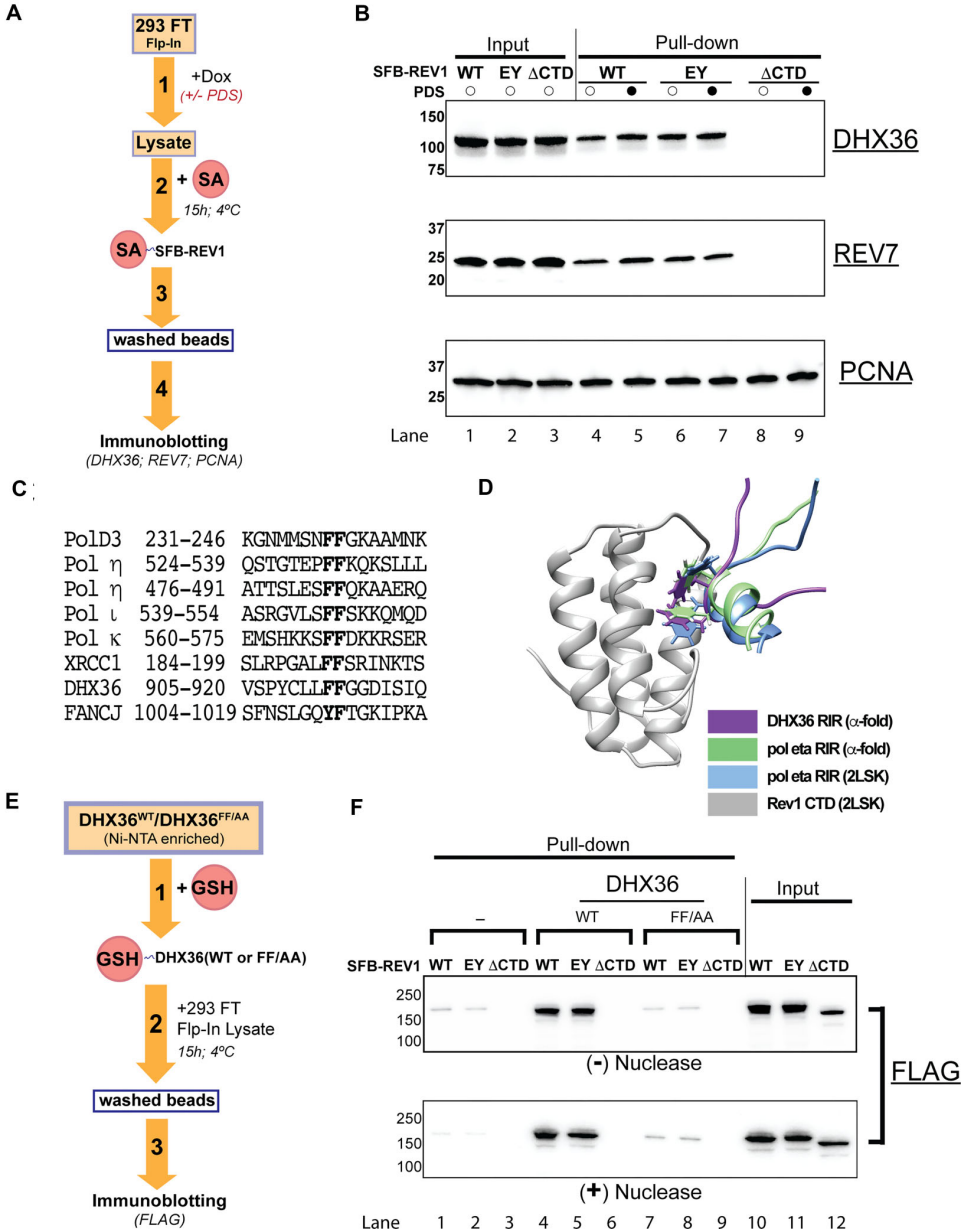
DHX36 interacts with the REV1 CTD through a RIR motif at its C-terminus. (**A**) A schematic diagram outlining the experimental scheme for pull-down assays with SFB-tagged REV1 is shown. (**B**) Immunoblots from streptavidin (SA) pull-down experiments are shown for samples from Flp-In 293 FT *REV1*^KO^ (6D6) cells re-engineered to express either WT or mutant REV1 proteins (EY and ΔCTD) upon treatment with doxycycline. Lanes marked WCL were loaded with 50 μg of whole cell lysates for the indicated SFB-REV1 Flp-In cells. The “IP” lanes were loaded with protein that eluted from streptavidin-coated agarose beads following incubation of lysates from cells expressing each of the three SFB-REV1 proteins. Lanes labeled with open circles indicate control (no PDS) samples, while black circles indicate the PDS-treated samples. The blot was probed with primary antibody against DHX36 to detect the amount of the helicase that co-immunoprecipitated with each of the three SFB-REV1 proteins under untreated or treated conditions. Similarly, the blots were also probed for pulldown for the pol ζ subunit Rev7, which is known to interact with the REV1 CTD, along with PCNA as a positive control that was expected to bind to WT and mutant forms of REV1. (**C**) The putative RIR for DHX36 was aligned with known RIRs. The conserved double-aromatic side chain residues in the RIRs are shown in bold. (**D**) An AlphaFold model of the DHX36 RIR (purple; aa 905–920) bound to the REV1 CTD (gray; aa 1158–1251) is shown superimposed on an AlphaFold model of the pol η RIR (green; aa 524–539). Both peptides are shown superimposed on the solution NMR structure of the pol η RIR in complex with the REV1 CTD (blue; PDB 2LSK). The phenylalanine side chains are shown in stick form for all three peptides. The predicted template modeling (pTM) scores for the AlphaFold-predicted DHX36 and pol η RIRs bound to the REV1 CTD were 0.78 and 0.81, respectively. (**E**) A schematic diagram of the experimental scheme for reciprocal pull-down assays with recombinant DHX36. Experiments were performed with both WT DHX36 and an RIR mutant (F912A/F913A, FF/AA). (**F**) Results are shown for pull-down experiments with WT and FF/AA DHX36. The 6×His-GST-tagged DHX36 proteins were immobilized on GSH-sepharose beads and incubated with lysates (± Pierce universal nuclease) from Flp-In 293 FT cells expressing SFB-tagged versions of either WT, EY, or ΔCTD REV1. Anti-FLAG immunoblots are shown, reporting on the amount of SFB-tagged REV1 retained on the DHX36-bound beads. 293 FT lysates were incubated with empty GSH-sepharose (i.e. no DHX36) to control for non-specific binding to the beads (Supplementary Fig. S12A).

We next determined if there was a physical interaction with DHX36 comparable to other known REV1-interactors, including other TLS pols [24, 48]. To examine this possibility, we performed pull-down experiments from Flp-In 293 FT *REV1*^KO^ (6D6) cells complemented with an inducible form of either SFB-tagged WT, EY, or ΔCTD REV1 (Fig. 8A). We confirmed that there was very little non-specific binding of DHX36 to the “empty” streptavidin beads (Supplementary Fig. S12A). Then we performed immunoblotting to determine if DHX36 bound to the three different versions of REV1. Input samples from each cell line were included to ensure equal expression of target proteins and equal loading of total protein was confirmed by Ponceau S staining of the blot (Supplementary Fig. S12B–D). We probed for the Rev7 subunit of pol ζ as a positive control for an interactor that is known to require the REV1 CTD. We also probed for PCNA as an interactor that did not require the REV1 CTD. Treatment with PDS (10 µM, 1 h) was performed to examine whether G4 stabilization impacted the relative amount of protein pulled down with the SFB-tagged REV1.

DHX36 was enriched in samples captured from cells expressing either the WT or EY mutant REV1 (Fig. 8B). Conversely, there was no DHX36 signal in samples captured from cells expressing the REV1 ΔCTD mutant (Fig. 8B). By way of comparison, PCNA was found to interact with all three versions of REV1, while Rev7 only bound to WT and the EY mutant (Fig. 8B), clearly demonstrating that the three different versions of REV1 behaved as expected. Treatment with PDS produced some modest increases in DHX36 and REV7 but no overt changes in the amount of target protein pulled down with REV1.

### An RIR near the DHX36 C-terminus is responsible for binding to REV1

Validation of a direct physical interaction between DHX36 and the REV1 CTD led us to look for a potential RIR motif in the human DHX36 sequence. Prior work reported that the consensus RIR motif is x-F-F-y-y-y-y, where y is any residue but proline [48]. The invariant RIR phenylalanine residues have been reported for pol η and pol κ, as well as other repair proteins like XRCC1 [48–50]. A motif from human FANCJ does not retain the consensus double phenylalanine and is able to bind both PCNA and the REV1 CTD [40]. Indeed, there is not much to distinguish RIRs from PCNA-interacting peptides (PIPs). Inspection of DHX36 sequence revealed a single, putative RIR motif in the C-terminal region that resembled canonical RIRs (Fig. 8C). The short RIR motif in the DHX36 sequence included two phenylalanine residues in tandem (F912 and F913) and was conserved across multiple metazoan species with some variation found in orthologs from fruit flies and goatgrass (Supplementary Fig. S13).

We used AlphaFold [51, 52] to model binding of the putative DHX36 RIR to the REV1 CTD. AlphaFold correctly reproduced the binding orientation of the pol η RIR (Fig. 8D, compare green and blue peptides), lending some confidence to the modeling exercise. An AlphaFold-generated model placed the DHX36 RIR motif in a conformation similar to the pol η RIR (Fig. 8D, purple peptide) [24]. To further confirm the functionality of the putative RIR, we performed pull-down experiments with DHX36.

WT DHX36 (aa 53–1008) and an RIR mutant (F912A/F913A or FF/AA) were expressed and partially purified (Supplementary Fig. S3A and B). Both versions of DHX36 possessed an N-terminal 6×His-GST tag, and both forms of DHX36 bound equally well to GSH sepharose beads (Supplementary Fig. S3C). We then incubated DHX36-bound GSH-beads with lysates from each of the three Flp-In cell lines induced to express WT, EY, and ΔCTD REV1, respectively (Fig. 8E). Pull-down experiments were performed with or without nuclease treatment to assess the nucleic acid dependence of the interaction. Empty GSH-beads were used to control for non-specific binding and input samples were loaded onto the gel to ensure comparable expression levels for REV1 proteins in the lysates. The resulting blots provide striking and clear evidence that DHX36 interacts with the REV1 CTD through the RIR at its C-terminus.

Both forms of REV1 possessing the CTD (WT and EY) were enriched on beads bound with WT DHX36, but the ΔCTD REV1 mutant was not pulled down (Fig. 8F). WT DHX36 was able to enrich WT and EY REV1 whether nuclease treatment was included or not, indicative of a direct interaction between REV1 and DHX36 that does not require DNA. Conversely, the FF/AA DHX36 mutant was unable to pull down any of the REV1 proteins. These results provided compelling evidence for a direct interaction between the REV1 CTD and the RIR at the C-terminus of DHX36.

### The affinity of a putative DHX36 RIR for the REV1 CTD was comparable to that observed for other interactors

**Figure 9.**
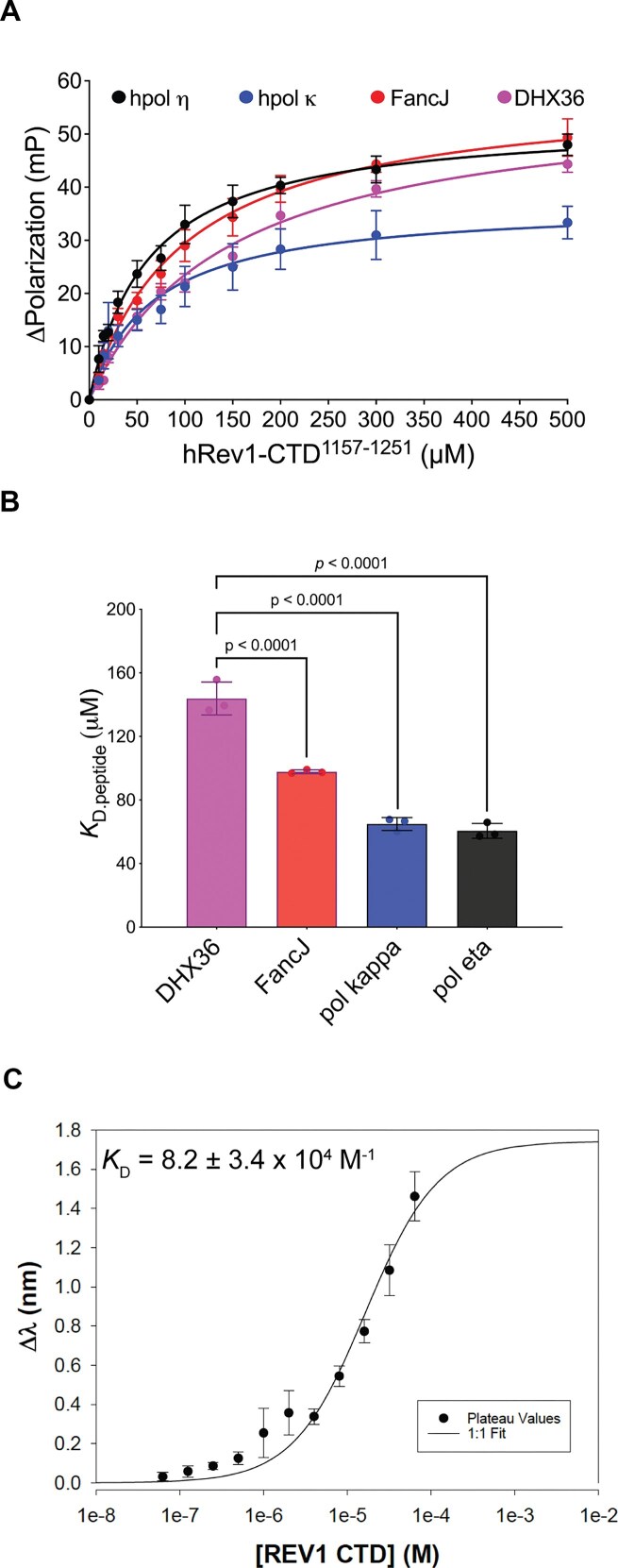
The RIR peptide from DHX36 can bind to the REV1 CTD with affinity comparable to other RIR motifs. (**A**) Fluorescence anisotropy was used to measure *K*_D_ values for binding of FAM-labeled DHX36, FANCJ, pol η, and pol κ peptides to the REV1-ΔCTD (aa 1149–1251). The sequences of the synthetic peptides used in the binding assay correspond to those shown in panel (A). Results were plotted as the change in fluorescence polarization signal occurring as a function of peptide concentration (*n* = 3). (**B**) The *K*_D_ values measured for each peptide RIR-REV1 CTD were re-plotted. The mean (± std. dev.) is shown alongside the individual measurements. *P*-values were calculated using a one-way ANOVA with Dunnett’s multiple comparisons test. (**C**) Biolayer interferometry experiments were performed with a biotinylated synthetic peptide derived from the DHX36 sequence (aa 906–924). The REV1-ΔCTD protein was titrated in and association/dissociation was monitored. The binding isotherm was fit to a 1:1 interaction model and the equilibrium dissociation constant (± 95% confidence interval) is shown from the average of three independent replicates.

We then set out to determine whether the affinity of the DHX36 RIR motif for the REV1 CTD was comparable to known RIRs. Fluorescence anisotropy experiments were performed with the purified REV1 CTD (aa 1149–1251) and FAM-labeled synthetic peptides (Fig. 9A). The resulting titration curves were fit to a quadratic equation to estimate the equilibrium dissociation constant (*K*_D_). The affinity of the DHX36 peptide for the REV1 CTD was comparable to that of the FANCJ RIR but less than that of the TLS pol peptides (Fig. 9B). The affinity of the DHX36 RIR for the REV1 CTD was also confirmed using BLI as an orthogonal approach (Fig. 9C). In summary, the biophysical experiments supported the feasibility of an interaction occurring through the putative DHX36 RIR and the REV1 CTD, but based on the absolute values measured here, such an interaction might be less stable than that occurring with pol η or pol κ.

### REV1 and DHX36 coordinate to prevent PrimPol fork elongation but REV1 operates independently of DHX36 to suppress PDS-induced ssDNA gaps

**Figure 10.**
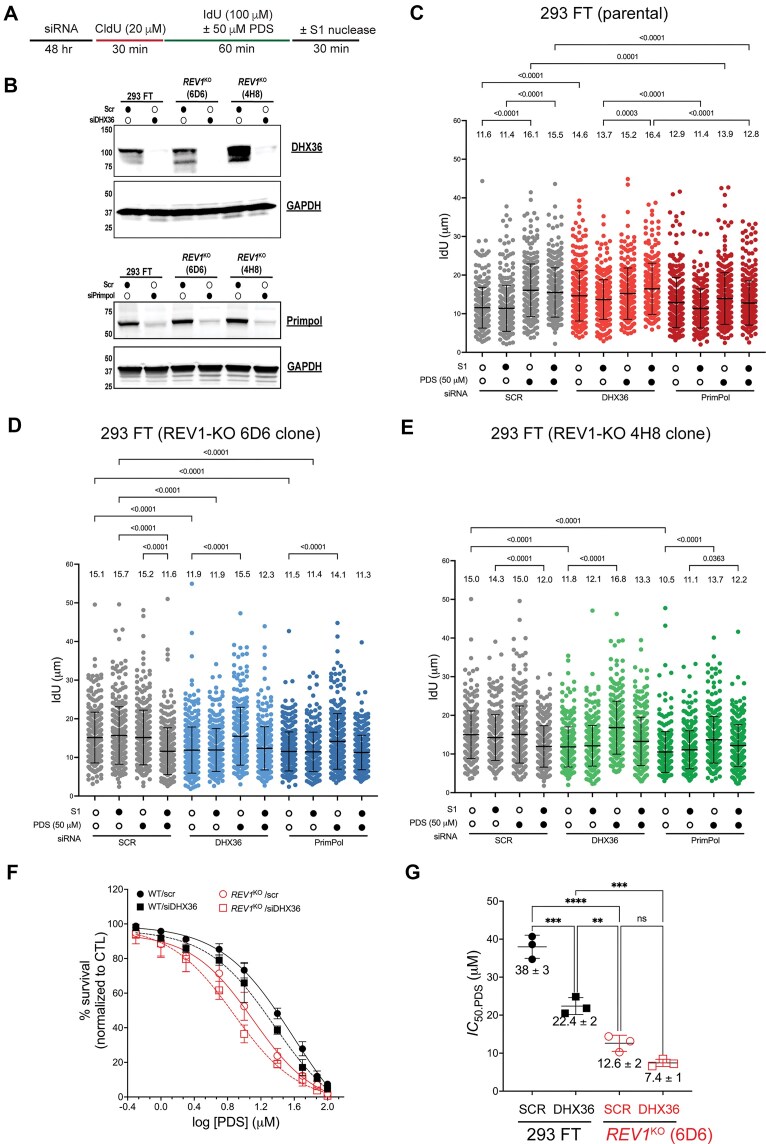
REV1 functions with DHX36 in on-the-fly bypass of endogenous fork barriers and functions independent of DHX36 to suppress PDS-induced ssDNA gaps. (**A**) A schematic illustration of the experimental design is shown for the DNA fiber spreading experiments. Experiments were performed using both 293 FT parental and both 4H8 and 6D6 *REV1*^KO^ cell lines (± S1 nuclease and ± PDS). PDS treatment was concurrent with the 60-min IdU pulse. (**B**) Immunoblotting results are shown confirming depletion of DHX36 and PrimPol by siRNA in parental 293 FT, as well as the two *REV1*^KO^ clones. The IdU track length is shown for (**C**) parental, (**D**) 6D6, and (**E**) 4H8 293 FT cell lines. The S1 nuclease, PDS, and RNAi statuses are noted for each experiment. Results shown are from two independent biological replicates where >225 fibers were scored per condition. The mean fiber length is noted above the data. Statistical significance was evaluated by performing a Kruskal–Wallis test followed by a Dunn’s multiple comparisons post hoc test. *P*-values <.05 are shown. (**F**) Clonogenic survival assay was performed with HEK293 FT parental and *REV1*^KO^ cells treated with scrambled siRNA (control) or DHX36-targeting siRNA, followed by exposure to varying concentrations of PDS. The results shown represent the mean (± std. dev.) of three independent biological replicates (black—parental; red—*REV1*^KO^). (**G**) The mean *IC*_50_ value (± std. dev.) for each condition is shown along with the individual means from three independent biological replicates. *P*-values were calculated by performing one-way ANOVA with a Tukey multiple comparisons test (ns—non-significant; **P = .0028; ***P = .0002; ****P < .0001).

To determine the functional effects of the REV1-DHX36 interaction on fork dynamics, we performed DFS (± S1 nuclease; ± PDS) in parental 293 FT and *REV1*^KO^ cells depleted of DHX36 by siRNA (Fig. 10A). Both 4H8 and 6D6 *REV1*^KO^ cell lines were used to ensure that the effects were not confined to a single knock-out clone. Immunoblotting was used to confirm depletion of DHX36 (Fig. 10B). Non-targeting (scrambled, SCR) siRNA was used as a control for any non-specific RNAi-related effects on fiber length.

Depletion of DHX36 produces aberrant fork elongation (i.e. longer fibers) in both vehicle- and PDS-treated 293 FT cells (Fig. 10C). The main difference between REV1 loss and DHX36 depletion is the lack of any ssDNA accumulation from PDS treatment, as evidenced by the fact that S1 nuclease treatment did not shorten fibers from DHX36-depleted parental 293 FT cells treated with PDS (Fig. 10C). Thus, parental 293 FT cells depleted of DHX36 are still able to suppress ssDNA gaps in response to G4 stabilization. We then considered the impact of losing both REV1 and DHX36 on fork elongation and ssDNA formation.

Both the 4H8 and 6D6 *REV1*^KO^ clones treated with SCR siRNA exhibited similar responses to vehicle and PDS treatment, including aberrant fork elongation and PDS-induced ssDNA gap formation (Fig. 10D and E, data on left side of each panel). Depletion of DHX36 eliminated the aberrant fork elongation phenotype in both *REV1*^KO^ clones (Fig. 10D and E, data sets in middle of each panel), supporting a functional connection between REV1 and DHX36 at endogenous fork barriers. PDS-treated *REV1*^KO^ 293 FT cells depleted of DHX36 with siRNA still have aberrantly long DNA fibers with persistent ssDNA, indicative of a role for REV1 in PDS-induced replication gap suppression that does not require DHX36.

Given the importance of PrimPol for aberrant fork elongation phenotypes like those we observed in *REV1*^KO^ and DHX36-depleted cells, we also performed experiments with siRNA targeting PrimPol (Fig. 10B). Knockdown of PrimPol had a minimal impact on basal fork speed and ssDNA gaps in untreated, parental 293 FT cells (Fig. 10C, data sets on right side of panel). In the REV1-proficient 293 FT parental cell line, loss of PrimPol reduced the effect of PDS on IdU track length from a 4.5 μm increase in scrambled control siRNA to 1.0 μm, suggestive of the fact that PrimPol causes much of the track-lengthening when REV1 is present and cells are exposed to PDS.

Depletion of PrimPol eliminated virtually all of the aberrant fork elongation observed in untreated *REV1*^KO^ 293 FT cells (Fig. 10D and E), indicative of a functional connection between REV1 and PrimPol at endogenous fork barriers. In *REV1*^KO^ PDS-treated cells, S1 nuclease shortened fibers by 3 μm or greater. By way of comparison, S1 nuclease shortened fibers by 2.8 μm and 1.5 μm in the 6D6 and 4H8 clones, respectively (Fig. 10D and E). Thus, loss of PrimPol caused a partial reduction in the ssDNA formation for the PDS-treated *REV1*^KO^ clones.

### REV1 and DHX36 exhibit partial overlap as determinants of sensitivity to G4 stabilization

Finally, the effect of DHX36 depletion on sensitivity of parental and *REV1*^KO^ cells to PDS was measured by the clonogenic survival assay (Fig. 10F and G). In parental 293 FT cells, depletion of DHX36 reduced the IC_50_ value from 38 μM (for scrambled siRNA) to 22 μM (Fig. 10F and G). The IC_50_ value for *REV1*^KO^ cells treated with scrambled control siRNA was 13 μM (Fig. 10F and G). Depletion of DHX36 in the *REV1*^KO^ cells reduced the IC_50_ a little more to around 7 μM (Fig. 10F and G). The difference between scrambled and DHX36 siRNA in *REV1*^KO^ cells was not statistically significant. However, the trend was toward an additive effect upon loss of both REV1 and DHX36. We interpret these results as evidence for a partial epistatic relationship between REV1 and DHX36 in determining the proliferative capacity of cells exposed to PDS, which is consistent with the dual roles for REV1 in DHX36-mediated bypass of endogenous lesions and DHX36-independent functions for REV1 in suppression of PDS-induced gaps. It is also consistent with the multi-faceted roles for DHX36 in G4 biology.

## Discussion

G4 motifs are found in functional regions of prokaryotic and eukaryotic genomes, indicative of a conservation of broad regulatory functions across species over long periods of evolutionary history [53, 54]. Dysregulation of quadruplex folding can arise through multiple mechanisms, including slow or stalled replication fork progression near G4 sites, which can produce vulnerable ssDNA gaps and interrupt epigenetic marks that regulate whether or not the G4 motif will fold within the context of chromatin [27, 55]. Maintenance of these motifs is important because an altered G4 landscape can have a significant impact on physiological processes. A prime example of this is dysregulation of oncogene expression and genomic instability derived from aberrant G4 folding in cancer [56, 57].

Although they clearly serve complex and important cellular functions, G4 DNA can be a source of replication stress and genomic instability [3, 4, 58–60]. Cells use a combination of helicase action and DNA repair/damage tolerance proteins to overcome challenges presented by G4 structures, including TLS, PrimPol repriming, and post-replicative gap filling. RNA-mediated maintenance of genomic G4 DNA has also emerged as key to proper regulation of these structured nucleic acids [61]. While these pathways are known to act in a coordinated fashion, the molecular mechanisms that couple G4 unwinding with DNA synthesis are not completely understood. In this study, we identify new features of REV1-mediated G4 replication that facilitate increased helicase engagement and suppression of ssDNA gaps.

### A multi-tiered model for REV1 function during bypass of G4 DNA

Our results support a model where REV1 has two partially separable roles in G4 replication: (i) facilitating efficient G4 unwinding to allow accurate and uninterrupted DNA synthesis and (ii) suppression of ssDNA gaps in response to G4 stabilization. As part of the first function, REV1 partners with DHX36. REV1 contributes to gap suppression independently from DHX36, suggestive of the fact that it contributes at least two mechanistically distinct functions during G4 bypass.

### Coordination of helicase activity and DNA synthesis by REV1

REV1 is part of a DNA damage tolerance pathway that can facilitate immediate and direct bypass of lesions and blocks to fork progression (i.e. “on-the-fly”). Using multiple experimental approaches, we demonstrate here that REV1 is a key determinant of DHX36 retention on chromatin and accumulation following G4 stabilization. This action depends on a direct interaction between the REV1 CTD and an RIR-motif near the C-terminus of DHX36. Loss of REV1 or deletion of the REV1 CTD reduces DHX36 accumulation within a few kb of nascent strand synthesis. The REV1-DHX36 interaction is key to preventing fork elongation by PrimPol, which in itself requires formation of ssDNA on the leading strand. Though not directly addressed by our study, others have demonstrated the importance of FANCJ in G4 unwinding during replication [46], including interactions with REV1 [28]. Thus, REV1 may help couple multiple G4 helicase activities with DNA synthesis.

The finding that loss of REV1 led to depletion of the DHX36 helicase on chromatin following treatment with PDS supported the idea that REV1 facilitated coordination of multiple G4 disruptive enzymes. A previous report found that FANCJ and DHX36 function together as part of the G4 unwinding and replication machinery in *Xenopus laevis* [46]. In that study, a major role for DHX36 was to support continued progression of the CMG helicase on the leading strand, but the mechanism by which the replisome activated DHX36 helicase activity was unknown. This is a key gap in our understanding of replisome mechanics because the presence of a single G4 in the leading strand stalls the yeast replisome [62].

One function of the REV1 might be to retain DHX36 (and other G4 helicases) long enough to prevent stalling of the replicative CMG helicase. Our results support a model where initial loading of DHX36 is facilitated by REV1 but over time, DHX36 moves away from the DNA synthesis machinery, which is consistent with a model where DHX36 moves ahead to create space for the CMG helicase to move past the G4 barrier even if DNA synthesis is slowed. Structures of yeast and human CMG helicases bound to G4 DNA revealed that a folded G4 structure was “lodged” in the central channel of a translocating CMG helicase [62]. Failing to maintain close coupling between DHX36 and the DNA synthesis machinery could increase the likelihood of a CMG-G4 structure that stalls fork progress and becomes even more challenging to resolve than the structured DNA alone. While our data do not directly report on pol-helicase coupling, they are consistent with a model where REV1 helps maintain coordination of these processes at endogenous fork barriers like G4 DNA.

### REV1-dependent suppression of ssDNA gaps in response to G4 stabilization

Another major conclusion of the current work is that REV1 is necessary for suppression of ssDNA gaps following G4 stabilization. While PrimPol was responsible for aberrant fork elongation in *REV1*^KO^ cells, loss of REV1 on its own did not seem to impede gap filling, as S1 nuclease treatment did not affect the track length in untreated *REV1*^KO^ cells. It was only after the addition of PDS that ssDNA gaps were left unfilled in cells devoid of REV1 activity. We interpret these results to mean that, when G4 structures were stabilized and hard to unwind, post-replicative G4 bypass was unable to efficiently copy quadruplexes without REV1. In a congruent line of thought, it is also possible that the gaps may become so large from uncoupling the TLS-PrimPol axis that post-replicative mechanisms simply cannot complete the gap-filling step. A defect in gap suppression was further supported by the persistence of CldU/ssDNA signal measured in REV1-deficient cells treated with PDS.

Recent work has demonstrated the interconnected nature of three RSR pathways involved in preventing persistent ssDNA on the leading strand: on-the-fly TLS, post-replicative lesion bypass, and PrimPol repriming [63]. In the proposed model, the effective hand-off between on-the-fly TLS and post-replicative lesion bypass depends on PrimPol-mediated repriming to keep the length of ssDNA gaps from becoming so large that they cannot be filled effectively [63]. Our findings support a similar conclusion for how REV1 and G4 helicase partnerships could work together to regulate the size of the zone acted on by PrimPol and keep gap-filling manageable.

The resulting effects on ssDNA gap formation may be due in part to the fact that *REV1*^KO^ cells have a generally more nuclear G4 signal than WT cells (Fig. 3A and B and Supplementary Fig. S5). As such, the fork in REV1-deficient cells may experience a more rugged genomic terrain to copy than in parental cells, which would necessitate increased G4 resolving capacity. Diminished DHX36 retention on chromatin would compound the problem by leaving *REV1*^KO^ cells at a disadvantage in terms of G4 unwinding capacity.

Whether REV1 itself facilitates mechanical disruption of the quadruplex structure in cells is not entirely clear. Biochemically, REV1 certainly seems capable of disrupting G4 structures, and we have observed that WT REV1 (but not the EY mutant) can stimulate DNA synthesis across a G4 motif by pol κ [31]. It is possible that G4 disruption by REV1 may be relatively passive in nature, more akin to what has been reported for RPA than the ATP hydrolysis-driven unwinding action of helicases [64–66]. In this regard, the scaffolding function of the REV1 CTD likely contributes more to tolerance of G4 stabilization than any tetrad-disruptive properties of REV1. Such a conclusion was supported by complementation experiments where the EY mutant was largely able to restore the PDS IC_50_ value for 293 FT cells to WT levels, but the PDS IC_50_ value for cells expressing the ΔCTD mutant resembled that of *REV1*^KO^ cells (Fig. 7E and F).

### Temporal features of G4 resolution and possible connections to G-loops

Cellular responses to G4 stabilization depend on the duration of the stress and genomic context, with resolution of G4-mediated fork barriers differing between early/mid and late S phase [67]. Processing of G4s outside of S-phase has also been observed for the fork-remodeling enzyme HLTF [68], in alignment with our finding that REV1-DHX36 PLA foci exist in both dividing (EdU+) and non-dividing (EdU−) cells (Fig. 6C–E). Other evidence points to the TLS enzyme pol η in the resolution of G-loop fork stalling at transcription-replication conflicts/collisions (TRCs) in early to mid S-phase, while late S-phase G4 barriers were overcome by PrimPol, leaving gaps to be filled by post-replicative repair [67].

**Figure 11.**
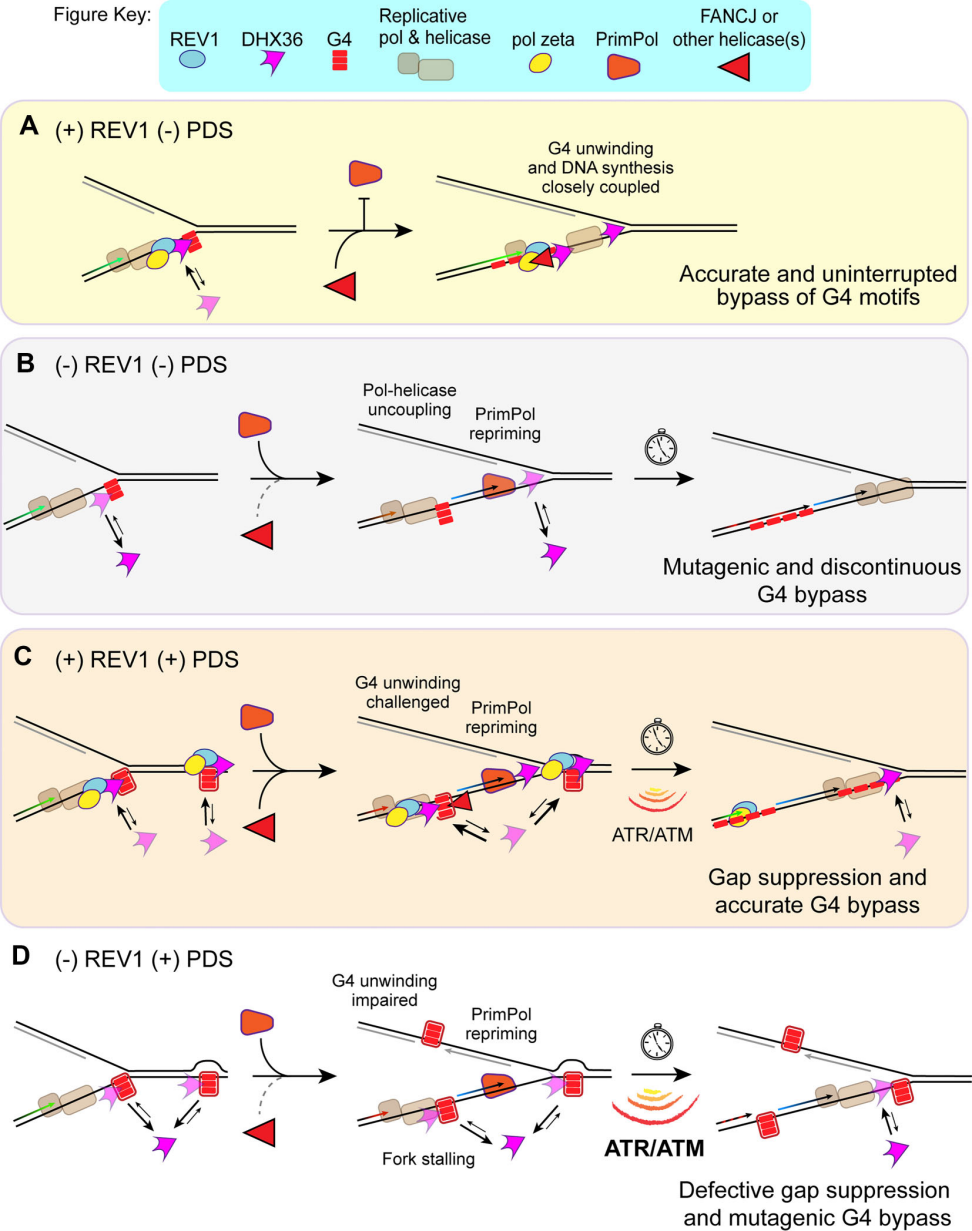
REV1 performs multiple functions during bypass of G4 DNA. A cartoon schematic is shown summarizing the potential interplay between REV1 and other G4 bypass factors, including DHX36. (**A**) When the fork encounters endogenous barriers like G4 DNA, REV1 partners with DHX36 and likely other helicases, such as FANCJ, to maintain close coupling between the DNA synthesis machinery and helicase action, limiting the formation of a PrimPol loading zone. This results in accurate and uninterrupted bypass of endogenous fork barriers. (**B**) A REV1-deficient replisome is less able to keep polymerase and helicase action coupled when barriers like G4 DNA are encountered, which results in PrimPol-mediated fork elongation and less accurate replication of G4 motifs. Mutagenic replication is heightened if the G4 motif is located on the leading strand in REV1-deficient cells. (**C**) G4 stabilization presents a challenge to fork progression, even when REV1 is present. DHX36 localization to chromatin depends on REV1. After an hour of G4 stabilization, DHX36 accumulates distal to REV1 and the DNA synthesis machinery. The presence of REV1 ultimately facilitates accurate G4 copying and suppression of ssDNA gaps. (**D**) When REV1 is absent, G4 stabilization causes major problems for the fork, as evidenced by elevated DNA damage and replication stress response signaling. DHX36 fails to accumulate on chromatin where and when it is needed if REV1 is not there to guide it. Without REV1, PDS treatment strains the accuracy of DNA synthesis and fork progression on both leading and lagging strand synthesis, resulting in persistent ssDNA gaps and mutagenic G4 replication occurring. These outcomes could reflect a defect in REV1-mediated dissolution of G4 structures or how effectively PrimPol can couple on-the-fly and post-replicative TLS mechanisms. The general layout of our schematic was informed and inspired by previously reported models [46, 63].

Based on our findings, REV1 may help facilitate both early and late resolution of G4 barriers. We propose that REV1 coordinates with DHX36 to facilitate close coupling of pol and helicase action, limiting the need for PrimPol repriming or limiting gap size to maintain uninterrupted fork progression (Fig. 11A). A related outcome of this REV1 function is accurate replication of the G4 motif, even when cells are exposed to PDS. We envision that loss of either REV1 (or DHX36) would allow PrimPol to gain access to the template DNA and advance the replication machinery (Fig. 11B). Given that DHX36 is a key G4 resolvase, the aberrant fork elongation observed in REV1 and DHX36-deficient cells could be attributed to uncoupling of pol-helicase action near G4 DNA. However, it is also possible that other endogenous fork barriers require REV1 and DHX36 activity.

Stabilization of G4s seems to uncouple pol-helicase action even when REV1 is present, as evidenced by PrimPol-dependent fork elongation in PDS-treated parental 293 FT cells (Fig. 11C). Access to the template strand by PrimPol may be due to slower forward progress by the DNA synthesis machinery, owing to increased stability and abundance of G4 DNA structures, which would present a greater challenge to G4 unwinders (Fig. 11C). Prolonged G4 stabilization seems to physically and functionally separate the REV1-DHX36 partnership, transitioning REV1 to an essential role in ssDNA gap suppression that does not require DHX36.

In the absence of REV1, PDS treatment likely causes defects in both leading and lagging strand G4 bypass that leads to aberrant fork elongation and persistent ssDNA (Fig. 11D). This conclusion is based, in part, on our observation that depletion of PrimPol reduces but does not eliminate persistent ssDNA in REV1-deficient cells treated with PDS, indicative of a defect in ssDNA gap suppression not solely tied to repriming on the leading strand. It is also based on the fact that the mutation frequency for leading strand G4 bypass in REV1-deficient cells is greatly elevated but insensitive to PDS treatment, while lagging strand G4 mutations increase in response to PDS treatment when REV1 is absent.

There are similarities between the relationships we observe and those reported for RNA-containing G-loop assembly/disassembly, including temporal resolution of DHX36/FANCJ G4 unwinding and DNA synthesis [61]. Though not pursued in detail here, we note that the methyltransferase METTL3, an enzyme responsible for methylation of RNA at N6-adenosine and a target of ATM [69], is enriched (alongside ATM) in iPOND samples from *REV1*^KO^ cells (Supplementary Fig. S10A and C). Thus, loss of REV1 and DHX36 may cause an accumulation of RNA-related structures near nascent strand synthesis. A role for REV1 and DHX36 in processing RNA-containing G-loops that precedes or is physically separate from REV1 gap filling at G4s would seem to fit with data reported here and elsewhere [61, 67].

### Coordination of multiple interactions involved in G4 bypass

Understanding how REV1 engages with and exchanges its binding partners is important for deciphering how its multi-faceted functions during G4 bypass could be partitioned. Early studies made it clear that pol ζ engages with REV1 in a manner that is very different from interactors like pol η and pol κ [22, 25, 70]. More recently, a cryo-EM structure of the REV1-pol ζ-DNA-dNTP holocomplex revealed that interactions between REV1 and pol ζ involve multiple subunits and molecular interfaces [26]. The pol ζ holoenzyme includes Rev3 (the catalytic subunit of pol ζ), Rev7 (the subunit bridging the REV1-3 interaction), and two subunits of the pol delta holoenzyme (PolD2 and PolD3). A reported structure of the REV1-pol ζ ternary complex revealed that, in addition to the CTD, the BRCT domain of REV1 also plays a key role in the REV1-pol ζ interaction [26], further illustrating the larger binding surface and more stable nature of the REV1-pol ζ complex relative to RIR-mediated interactions.

The models in Fig. 11 present a single REV1 engaged at the fork coordinating many replication factors, some with overlapping binding sites. Such a model would require dynamic exchange of different partners (i.e. DHX36 is replaced by FANCJ, which is then replaced by a TLS pol, etc.). However, we cannot rule out the possibility that multiple REV1 molecules are engaged at or near the fork, each with a different set of binding partners. REV1 is capable of binding both ssDNA and primer-template DNA, in some instances exhibiting tighter binding to G4 in the ssDNA context compared to G4 adjacent to a primer-template junction [29, 30, 31]. It may be that the dual roles for REV1 identified here involve more than one binding mode: binding near G4 in the ssDNA ahead of the fork (DHX36-dependent) and binding to G4 formed near stalled primer-template junctions (DHX36-independent gap filling).

Our results support the idea that the DHX36 RIR can bind to the REV1 CTD in a manner that is similar to RIRs from Y-family pol η and pol κ (Fig. 8D). It is reasonable to suppose that REV1 enhances localization of G4 helicases to stalled forks where the translocation and unwinding action of these enzymes gives way to REV1-coordinated primer extension by TLS polymerases. Still, the exact molecular nature of the REV1-DHX36 interaction remains an open question. It is not obvious from existing DHX36 structures how the RIR would engage with the REV1 CTD, as it is not surface exposed (Supplementary Fig. S14). A conformational change would be required to facilitate binding of the DHX36 RIR with the REV1 CTD. Returning to the speculation that the replisome “plays a direct role” in DHX36 activation posed by the Knipscheer group in their 2021 study [46], it is possible that the interaction with REV1 helps to activate DHX36 when the replisome encounters a stable G4 structure, perhaps by inducing a conformational change that exposes the RIR. Alternatively, the sequestration of the RIR away from REV1 in response to some other stimulus could release DHX36 ahead of the replisome to allow CMG progression and binding of the next REV1 interactor.

Whatever the structural effects might be, the results reported here support a model where REV1 coordinates replication of G4 structures by assisting with retention of DHX36, pol ζ, and likely other factors like FANCJ and other Y-family pols to (i) maintain close-coupled pol-helicase action and fork progression, (ii) accurate G4 replication, and (iii) suppress formation of persistent ssDNA gaps following G4 stabilization. REV1 maintains basal levels of DHX36 on chromatin, and this influences accumulation of DHX36 when cells were treated with PDS. When PDS treatment is prolonged, DHX36 accumulates distal to REV1 and the DNA synthesis machinery. REV1 assumes an essential role in preventing ssDNA gaps formed by G4 stabilization that is independent of DHX36. Biochemical and structural studies combining the replication factors involved in G4 bypass are still needed. Answers to other questions related to how REV1-interactors are positioned spatially and function temporally in the cell will be important to resolve if we are to gain ever more precise insights into fork dynamics during G4 replication.

## Supplementary Material

gkag562_Supplemental_Files

## Data Availability

Unaltered images, novel molecular models, and data that pertain to figures and graphs in the manuscript are available through the Open Science Foundation at https://osf.io/kadrs/ (DOI: 10.17605/OSF.IO.KADRS). The mass spec proteomics data underlying this article are available in PRIDE at (https://www.ebi.ac.uk/pride/), and can be accessed with project accession number PXD061281.

## References

[1] Berti M, Cortez D, Lopes M. The plasticity of DNA replication forks in response to clinically relevant genotoxic stress. Nat Rev Mol Cell Biol. 2020;21:633–51. 10.1038/s41580-020-0257-5 32612242

[2] Chang DJ, Cimprich KA. DNA damage tolerance: when it’s OK to make mistakes. Nat Chem Biol. 2009;5:82–90. 10.1038/nchembio.139 19148176 PMC2663399

[3] Lopes J, Piazza A, Bermejo R et al. G-quadruplex-induced instability during leading-strand replication. EMBO J. 2011;30:4033–46. 10.1038/emboj.2011.316 21873979 PMC3209785

[4] Paeschke K, Bochman ML, Garcia PD et al. Pif1 family helicases suppress genome instability at G-quadruplex motifs. Nature. 2013;497:458–62. 10.1038/nature12149 23657261 PMC3680789

[5] Lerner LK, Sale JE. Replication of G Quadruplex DNA. Genes. 2019;10:95. 10.3390/genes10020095 30700033 PMC6409989

[6] Fleming AM, Zhou J, Wallace SS et al. A role for the fifth G-track in G-quadruplex forming oncogene promoter sequences during oxidative stress: do these ‘spare tires’ have an evolved function?. ACS Cent. Sci. 2015;1:226–33. 10.1021/acscentsci.5b00202 26405692 PMC4571166

[7] Fleming AM, Burrows CJ. Oxidative stress-mediated epigenetic regulation by G-quadruplexes. NAR Cancer. 2021;3:zcab038. 10.1093/narcan/zcab038 34541539 PMC8445369

[8] Murat P, Balasubramanian S. Existence and consequences of G-quadruplex structures in DNA. Curr Opin Genet Dev. 2014;25:22–9. 10.1016/j.gde.2013.10.012 24584093

[9] Fujii S, Fuchs RP. A comprehensive view of translesion synthesis in *Escherichia coli*. Microbiol Mol Biol Rev. 2020;84:e00002–20. 10.1128/MMBR.00002-20 32554755 PMC7307797

[10] Goodman MF, Woodgate R. Translesion DNA polymerases. Cold Spring Harb Perspect Biol. 2013;5:a010363. 10.1101/cshperspect.a010363 23838442 PMC3783050

[11] Sale JE, Lehmann AR, Woodgate R. Y-family DNA polymerases and their role in tolerance of cellular DNA damage. Nat Rev Mol Cell Biol. 2012;13:141–52. 10.1038/nrm3289 22358330 PMC3630503

[12] Foti JJ, Devadoss B, Winkler JA et al. Oxidation of the guanine nucleotide pool underlies cell death by bactericidal antibiotics. Science. 2012;336:315–9. 10.1126/science.1219192 22517853 PMC3357493

[13] Xie K, Doles J, Hemann MT et al. Error-prone translesion synthesis mediates acquired chemoresistance. Proc Natl Acad Sci USA. 2010;107:20792–7. 10.1073/pnas.1011412107 21068378 PMC2996453

[14] Peng C, Chen Z, Wang S et al. The error-prone DNA polymerase κ promotes temozolomide resistance in glioblastoma through Rad17-dependent activation of ATR–Chk1 signaling. Cancer Res. 2016;76:2340–53. 10.1158/0008-5472.CAN-15-1884 26960975

[15] Somyajit K, Spies J, Coscia F et al. Homology-directed repair protects the replicating genome from metabolic assaults. Dev Cell. 2021;56:461–77. 10.1016/j.devcel.2021.01.011 33621493

[16] Waters LS, Walker GC. The critical mutagenic translesion DNA polymerase Rev1 is highly expressed during G(2)/M phase rather than S phase. Proc Natl Acad Sci USA. 2006;103:8971–6. 10.1073/pnas.0510167103 16751278 PMC1482550

[17] Petljak M, Dananberg A, Chu K et al. Mechanisms of APOBEC3 mutagenesis in human cancer cells. Nature. 2022;607:799–807. 10.1038/s41586-022-04972-y 35859169 PMC9329121

[18] Sharma S, Canman CE. REV1 and DNA polymerase zeta in DNA interstrand crosslink repair. Environ and Mol Mutagen. 2012;53:725–40. 10.1002/em.21736 PMC5543726 23065650

[19] Nair DT, Johnson RE, Prakash L et al. Rev1 employs a novel mechanism of DNA synthesis using a protein template. Science. 2005;309:2219–22. 10.1126/science.1116336 16195463

[20] Swan MK, Johnson RE, Prakash L et al. Structure of the human Rev1–DNA–dNTP ternary complex. J Mol Biol. 2009;390:699–709. 10.1016/j.jmb.2009.05.026 19464298 PMC2739620

[21] D’Souza S, Walker GC. Novel role for the C terminus of *Saccharomyces cerevisiae* Rev1 in mediating protein–protein interactions. Mol Cell Biol. 2006;26:8173–82. 16923957 10.1128/MCB.00202-06 PMC1636727

[22] Pustovalova Y, Bezsonova I, Korzhnev DM. The C-terminal domain of human Rev1 contains independent binding sites for DNA polymerase η and Rev7 subunit of polymerase ζ. FEBS Lett. 2012;586:3051–6. 10.1016/j.febslet.2012.07.021 22828282 PMC3572780

[23] Ross A-L, Simpson LJ, Sale JE. Vertebrate DNA damage tolerance requires the C-terminus but not BRCT or transferase domains of REV1. Nucleic Acids Res. 2005;33:1280–9. 10.1093/nar/gki279 15741181 PMC552965

[24] Pozhidaeva A, Pustovalova Y, D’Souza S et al. NMR structure and dynamics of the C-terminal domain from human Rev1 and its complex with Rev1 interacting region of DNA polymerase η. Biochemistry. 2012;51:5506–20. 10.1021/bi300566z 22691049 PMC3732116

[25] Pustovalova Y, Magalhães MTQ, D’Souza S et al. Interaction between the Rev1 C-terminal domain and the PolD3 subunit of Polζ suggests a mechanism of polymerase exchange upon Rev1/Polζ-dependent translesion synthesis. Biochemistry. 2016;55:2043–53. 10.1021/acs.biochem.5b01282 26982350 PMC4898654

[26] Malik R, Johnson RE, Ubarretxena-Belandia I et al. Cryo-EM structure of the Rev1–Polζ holocomplex reveals the mechanism of their cooperativity in translesion DNA synthesis. Nat Struct Mol Biol. 2024;31:1394–403. 10.1038/s41594-024-01302-w 38720088 PMC12068682

[27] Sarkies P, Reams C, Simpson LJ et al. Epigenetic instability due to defective replication of structured DNA. Mol Cell. 2010;40:703–13. 10.1016/j.molcel.2010.11.009 21145480 PMC3145961

[28] Sarkies P, Murat P, Phillips LG et al. FANCJ coordinates two pathways that maintain epigenetic stability at G-quadruplex DNA. Nucleic Acids Res. 2012;40:1485–98. 10.1093/nar/gkr868 22021381 PMC3287192

[29] Ketkar A, Smith L, Johnson C et al. Human Rev1 relies on insert-2 to promote selective binding and accurate replication of stabilized G-quadruplex motifs. Nucleic Acids Res. 2021;49:2065–84. 10.1093/nar/gkab041 33555350 PMC7913688

[30] Eddy S, Ketkar A, Zafar MK et al. Human Rev1 polymerase disrupts G-quadruplex DNA. Nucleic Acids Res. 2014;42:3272–85. 10.1093/nar/gkt1314 24366879 PMC3950705

[31] Ketkar A, Sewilam RS, McCrury MJ et al. Conservation of the insert-2 motif confers Rev1 from different species with an ability to disrupt G-quadruplexes and stimulate translesion DNA synthesis. RSC Chem. Biol. 2023;4:466–85. 10.1039/D3CB00027C 37415867 PMC10320842

[32] Ran FA, Hsu PD, Wright J et al. Genome engineering using the CRISPR–Cas9 system. Nat Protoc. 2013;8:2281–308. 10.1038/nprot.2013.143 24157548 PMC3969860

[33] Quinet A, Carvajal-Maldonado D, Lemacon D et al. DNA fiber analysis: mind the Gap!. Methods Enzymol. 2017;591:55–82. 28645379 10.1016/bs.mie.2017.03.019

[34] Nayak S, Calvo JA, Cong K et al. Inhibition of the translesion synthesis polymerase REV1 exploits replication gaps as a cancer vulnerability. Sci. Adv. 2020;6:eaaz7808. 10.1126/sciadv.aaz7808 32577513 PMC7286678

[35] Sirbu BM, Couch FB, Feigerle JT et al. Analysis of protein dynamics at active, stalled, and collapsed replication forks. Genes Dev. 2011;25:1320–7. 10.1101/gad.2053211 21685366 PMC3127432

[36] Dungrawala H, Cortez D. Purification of proteins on newly synthesized DNA using iPOND. Methods Mol Biol. 2015;1228:123–31. 25311126 10.1007/978-1-4939-1680-1_10 PMC4384176

[37] Schindelin J, Arganda-Carreras I, Frise E et al. Fiji: an open-source platform for biological-image analysis. Nat Methods. 2012;9:676–82. 10.1038/nmeth.2019 22743772 PMC3855844

[38] Roy S, Schlacher K. SIRF: a single-cell assay for *in situ* protein interaction with nascent DNA replication forks. Bio Protoc. 2019;9:e3377. 10.21769/BioProtoc.3377 PMC7854004 33654873

[39] Roy S, Luzwick JW, Schlacher K. SIRF: quantitative *in situ* analysis of protein interactions at DNA replication forks. J Cell Biol. 2018;217:1521–36. 10.1083/jcb.201709121 29475976 PMC5881507

[40] Lowran K, Campbell L, Popp P et al. Assembly of a G-quadruplex repair complex by the FANCJ DNA helicase and the REV1 polymerase. Genes. 2019;11:5. 10.3390/genes11010005 31861576 PMC7017153

[41] Schiavone D, Guilbaud G, Murat P et al. Determinants of G quadruplex-induced epigenetic instability in REV1-deficient cells. EMBO J. 2014;33:2507–20. 10.15252/embj.201488398 25190518 PMC4282387

[42] Seidman MM, Dixon K, Razzaque A et al. A shuttle vector plasmid for studying carcinogen-induced point mutations in mammalian cells. Gene. 1985;38:233–7. 10.1016/0378-1119(85)90222-7 2998945

[43] Biffi G, Tannahill D, McCafferty J et al. Quantitative visualization of DNA G-quadruplex structures in human cells. Nature Chem. 2013;5:182–6. 10.1038/nchem.1548 23422559 PMC3622242

[44] Hänsel-Hertsch R, Beraldi D, Lensing SV et al. G-quadruplex structures mark human regulatory chromatin. Nat Genet. 2016;48:1267–72. 10.1038/ng.3662 27618450

[45] Dungrawala H, Rose KL, Bhat KP et al. The replication checkpoint prevents two types of fork collapse without regulating replisome stability. Mol Cell. 2015;59:998–1010. 10.1016/j.molcel.2015.07.030 26365379 PMC4575883

[46] Sato K, Martin-Pintado N, Post H et al. Multistep mechanism of G-quadruplex resolution during DNA replication. Sci Adv. 2021;7:eabf8653. 10.1126/sciadv.abf8653 34559566 PMC8462899

[47] Sirbu BM, Couch FB, Cortez D. Monitoring the spatiotemporal dynamics of proteins at replication forks and in assembled chromatin using isolation of proteins on nascent DNA. Nat Protoc. 2012;7:594–605. 10.1038/nprot.2012.010 22383038 PMC3671908

[48] Ohashi E, Hanafusa T, Kamei K et al. Identification of a novel REV1-interacting motif necessary for DNA polymerase kappa function. Genes Cells. 2009;14:101–11. 10.1111/j.1365-2443.2008.01255.x 19170759 PMC3103050

[49] Gabel SA, DeRose EF, London RE. XRCC1 interaction with the REV1 C-terminal domain suggests a role in post replication repair. DNA Repair. 2013;12:1105–13. 10.1016/j.dnarep.2013.08.015 24409475 PMC5572806

[50] Bi T, Niu X, Qin C et al. Genetic and physical interactions between Polη and Rev1 in response to UV-induced DNA damage in mammalian cells. Sci Rep. 2021;11:21364. 10.1038/s41598-021-00878-3 34725419 PMC8560953

[51] Jumper J, Evans R, Pritzel A et al. Highly accurate protein structure prediction with AlphaFold. Nature. 2021;596:583–9. 10.1038/s41586-021-03819-2 34265844 PMC8371605

[52] Abramson J, Adler J, Dunger J et al. Accurate structure prediction of biomolecular interactions with AlphaFold 3. Nature. 2024;630:493–500. 10.1038/s41586-024-07487-w 38718835 PMC11168924

[53] Capra JA, Paeschke K, Singh M et al. G-quadruplex DNA sequences are evolutionarily conserved and associated with distinct genomic features in *Saccharomyces cerevisiae*. PLoS Comput Biol. 2010;6:e1000861. 10.1371/journal.pcbi.1000861 20676380 PMC2908698

[54] Makova KD, Weissensteiner MH. Noncanonical DNA structures are drivers of genome evolution. Trends Genet. 2023;39:109–24. 10.1016/j.tig.2022.11.005 36604282 PMC9877202

[55] Schiavone D, Jozwiakowski SK, Romanello M et al. PrimPol is required for replicative tolerance of G Quadruplexes in vertebrate cells. Mol Cell. 2016;61:161–9. 10.1016/j.molcel.2015.10.038 26626482 PMC4712188

[56] Biffi G, Tannahill D, Miller J et al. Elevated levels of G-quadruplex formation in human stomach and liver cancer tissues. PLoS One. 2014;9:e102711. 10.1371/journal.pone.0102711 25033211 PMC4102534

[57] Hänsel-Hertsch R, Simeone A, Shea A et al. Landscape of G-quadruplex DNA structural regions in breast cancer. Nat Genet. 2020;52:878–83. 10.1038/s41588-020-0672-8 32747825

[58] Rider SD, Gadgil RY, Hitch DC et al. Stable G-quadruplex DNA structures promote replication-dependent genome instability. J Biol Chem. 2022;298:101947. 10.1016/j.jbc.2022.101947 35447109 PMC9142560

[59] Stein M, Eckert KA. Impact of G-quadruplexes and chronic inflammation on genome instability: additive effects during carcinogenesis. Genes. 2021;12:1779. 10.3390/genes12111779 34828385 PMC8619830

[60] Linke R, Limmer M, Juranek SA et al. The relevance of G-quadruplexes for DNA repair. Int J Mol Sci. 2021;22:12599. 10.3390/ijms222212599 34830478 PMC8620898

[61] Sato K, Lyu J, van den Berg J et al. RNA transcripts regulate G-quadruplex landscapes through G-loop formation. Science. 2025;388:1225–31. 10.1126/science.adr0493 40504899

[62] Batra S, Allwein B, Kumar C et al. G-quadruplex-stalled eukaryotic replisome structure reveals helical inchworm DNA translocation. Science. 2025;387:eadt1978. 10.1126/science.adt1978 40048517 PMC12338045

[63] Mellor C, Nassar J, Šviković S et al. PRIMPOL ensures robust handoff between on-the-fly and post-replicative DNA lesion bypass. Nucleic Acids Res. 2024;52:243–58. 10.1093/nar/gkad1054 37971291 PMC10783524

[64] Safa L, Gueddouda NM, Thiébaut F et al. 5′ to 3′ unfolding directionality of DNA secondary structures by replication protein A: G-QUADRUPLEXES AND DUPLEXES. J Biol Chem. 2016;291:21246–56. 10.1074/jbc.M115.709667 27440048 PMC5076531

[65] Ray S, Qureshi MH, Malcolm DW et al. RPA-mediated unfolding of systematically varying G-quadruplex structures. Biophys J. 2013;104:2235–45. 10.1016/j.bpj.2013.04.004 23708363 PMC3660638

[66] Prakash A, Kieken F, Marky LA et al. Stabilization of a G-quadruplex from unfolding by replication protein A using potassium and the porphyrin TMPyP4. J Nucleic Acids. 2011;2011:1. 10.4061/2011/529828 PMC3136172 21772995

[67] Pepe S, Guerra F, Russo M et al. Genomic context influences translesion synthesis DNA polymerase-dependent mechanisms of micronuclei induction by G-quadruplexes. Cell Rep. 2025;44:115706. 10.1016/j.celrep.2025.115706 40349342

[68] Bai G, Endres T, Kühbacher U et al. HLTF resolves G4s and promotes G4-induced replication fork slowing to maintain genome stability. Mol Cell. 2024;84:3044–60. 10.1016/j.molcel.2024.07.018 39142279 PMC11366124

[69] Zhang C, Chen L, Peng D et al. METTL3 and *N*^6^-methyladenosine promote homologous recombination-mediated repair of DSBs by modulating DNA–RNA hybrid accumulation. Mol Cell. 2020;79:425–42. 10.1016/j.molcel.2020.06.017 32615088

[70] Kikuchi S, Hara K, Shimizu T et al. Structural basis of recruitment of DNA polymerase ζ by interaction between REV1 and REV7 proteins. J Biol Chem. 2012;287:33847–52. 10.1074/jbc.M112.396838 22859296 PMC3460479

