# Supplementary material for: Human REV1 interacts with DHX36 to promote replication and tolerance of G-quadruplex DNA
Source: Nucleic Acids Res. 2026 Jun 8;54(11):gkag562. doi: 10.1093/nar/gkag562 (PMC13244153; doi:10.1093/nar/gkag562)
Supplement: gkag562_Supplemental_Files [file gkag562_supplemental_files.zip › 260430 Rev1 interacts with DHX36 SI v2.pdf]

Supplementary materials for

Human Rev1 interacts with DHX36 to promote replication and tolerance of G-quadruplex DNA

Amit Ketkar[1], Bethany C. Paxton[1], Oscar E. Zuniga[1], Reham S. Sewilam[1], Martin Morales[1], John Schaller[1], Rowan J. McCollum[1], Kathleen E. Jackson[1], Kaitlin Lowran[2], Alyssa Paul[2], Meera Patel[3], Sreevatsav Seenivasan[1], Mason McCrury[1], Qudes Al-Anbaky[1], Leena Maddukuri[1], Samantha Kendrick[1], Colin G. Wu[2], Julie E.C. Gunderson[4], and Robert L. Eoff[1,*]

[1] Department of Biochemistry and Molecular Biology, University of Arkansas for Medical Sciences, Little Rock, AR 72205 USA

[2] Department of Chemistry, Oakland University, Rochester, MI 48309 USA

[3] Arkansas School for Mathematics, Sciences, and the Arts, Hot Springs, AR 71901 USA

[4] Department of Physics, Hendrix College, Conway, AR 72032, USA

* To whom correspondence should be addressed. Tel: +1 501 686 8343; Fax: +1 501 686 8169; Email: RLEoff@uams.edu

Running title: Rev1-dependent G4 replication interactions

SUPPORTING INFORMATION

CONTENTS

Supplementary Materials and Methods

Table S1. Mass spectrometry data from iPOND experiments

Figure S1. Generation and validation of the *REV1*^{KO} 293 FT cell line

Figure S2. Schematic for generation of *REV1*^{KO} 293 FT cell lines with stable integration of genes encoding WT and mutant SFB-Rev1 under the control of a tetracycline-inducible expression system

Figure S3. Purification of recombinant DHX36 (a.a. 53-1008)

Figure S4. Purification of recombinant REV1 CTD (a.a. 1157-1251)

Figure S5. BG4 immunofluorescences results for 293 FT cells

Figure S6. Representative images for clonogenic survival assay of 293 FT cells in the presence of three different G4 stabilizers

Figure S7. Analysis of differential protein abundance for parental WT and *REV1*^{KO} 293 FT iPOND-MS experiments

Figure S8. Immunoblots for the input samples for iPOND with parental WT and *REV1*^{KO} 293 FT cells, as well as the Flp-In cells expressing SFB-tagged REV1 proteins

Figure S9. Immunoblots and quantification for the iPOND experiment repeated in HAP1 WT and *REV1*^{KO} cells

Figure S10. Validation of additional hits from the iPOND-MS experiments by immunoblotting

Figure S11. REV1-DHX36 PLA results from U2OS cells

Figure S12. Control experiments for streptavidin pull-downs

Figure S13. DHX36 sequence alignment

Figure S14. Position of putative RIR region in apo- and G4-bound structures of DHX36

Supplementary Materials and Methods

PCR Validation of *REV1*^{KO} Cells

As a validation of CRISPR-Cas9 genomic editing, we designed PCR reactions that would give distinct product signatures for edited and unedited cells. Briefly, genomic DNA was isolated using ThermoScientific GeneJET Genomic DNA Purification Kit (ThermoScientific, #K0721) according to manufacturer's instructions. Two PCR primers were designed to target different regions of the CRISPR-Cas9 target. Genomic DNA (100 ng) was combined with each primer (2.5 µM) and Phusion High-Fidelity PCR Master Mix with HF buffer (ThermoScientific, #F-531-L) in a total volume of 50 µL.

Primer sequences:

- Forward primer PCR1 (369 bp product): 5′- CGGCAGAACCTATAAAGCATC -3′ (in reverse complement strand)
- Forward primer PCR2 (489 bp product): 5′-GAGCAACAGATGGCAAAATCTG -3′
- Reverse primer: 5′- GGTTCATCTATATGTGGAGTCC -3′

The PCR protocol is as follows: initial denaturation for 1 minute at 98 °C, followed by 35 cycles of 30 seconds of denaturation at 98 °C, 1 minute annealing at 55 °C, and 5 minutes extension at 72 °C, followed by a final 10 minute extension at 72 °C, and a 4 °C hold. The PCR product was combined with 6X loading due and run on 1% (w/v) agarose gels for 1 hour at 100 V. Gels were imaged using a ChemiDoc Digital Imager (BioRad, #12003153).

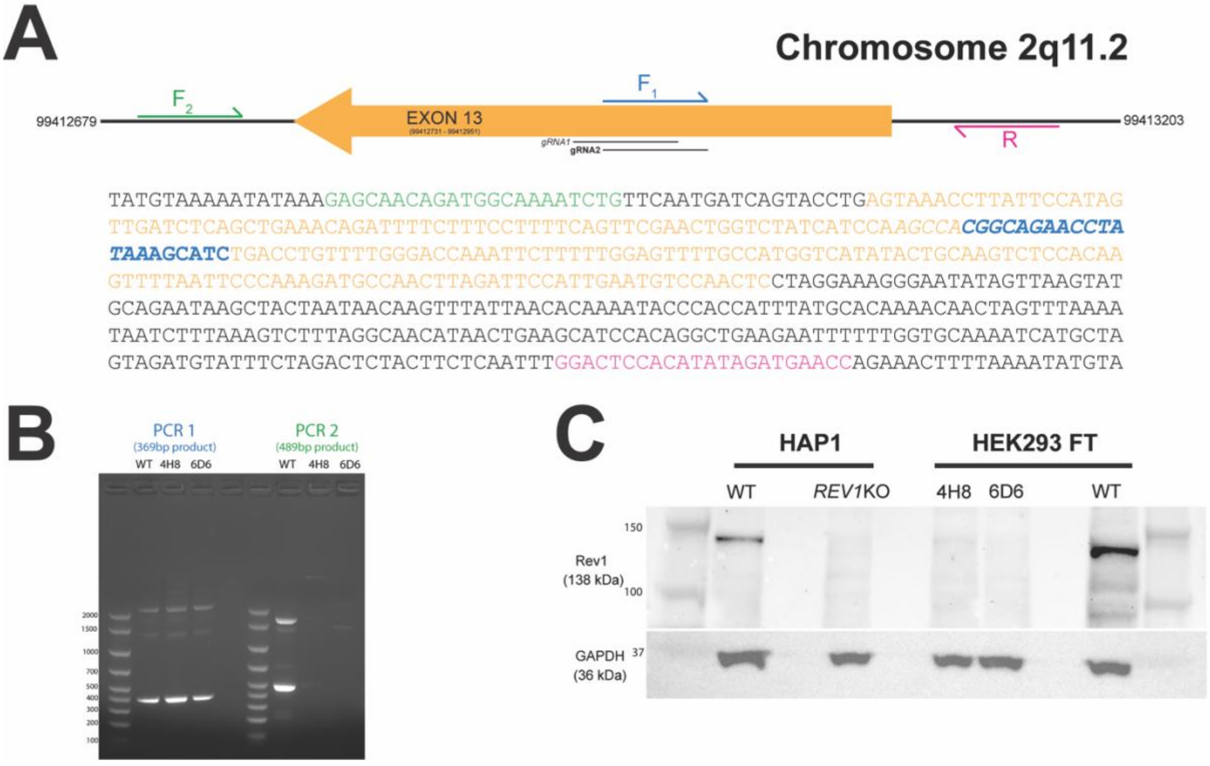

Figure S1. Construction and validation of the *REV1*^{KO} 293 FT cell line.

(**A**) Schematic illustration showing the DNA sequence of the genomic region targeting the *REV1* gene for editing by CRISPR-Cas9 technology. The ATUM server was used to obtain target sequences and design the single-guide RNA (sgRNA) specifying primers. The top two hits as predicted by the ATUM server were used, with the following sequences: gRNA1 – AGCCACGGCAGAACCTATAA; gRNA2 – CGGCAGAACCTATAAAGCATC. Positions of the sequence corresponding the two forward (F1 and F2) and one reverse (R) primers designed for PCR are shown in green and red respectively.

(**B**) Results of confirmation of gene knockout of *REV1* were obtained by PCR amplification of the region surrounding the sgRNA target site using genomic DNA extracted from each cell type, and two pairs of primers. PCR1 was performed using primers F1 and R, while PCR2 was performed with F2 and R. Amplicon sizes predicted were 369 bp and 489 bp respectively, for PCR1 and PCR2, in the control reactions (Parental). If the given clone was a true knockout, it would result in imperfect annealing of the primer F2, hence would result in poor amplicon product in PCR2. This is indeed seen for the two clones 4H8 and 6D6.

(**C**) Immunoblotting was also used to confirm depletion of the REV1 protein in the 'positive' clones of FT 293 cells. After extracting whole cell lysates from the Parental and KO-clones of 293 FT cells, protein estimation was done using BCS colorimetric assay. Equal amount of total protein was loaded for each sample (50 mg). Similar lysates obtained from the HAP1 Parental and *REV1*^{KO} cells (previously purchased from Horizon Discovery) were also loaded as controls. The blots were also probed for GAPDH as loading control.

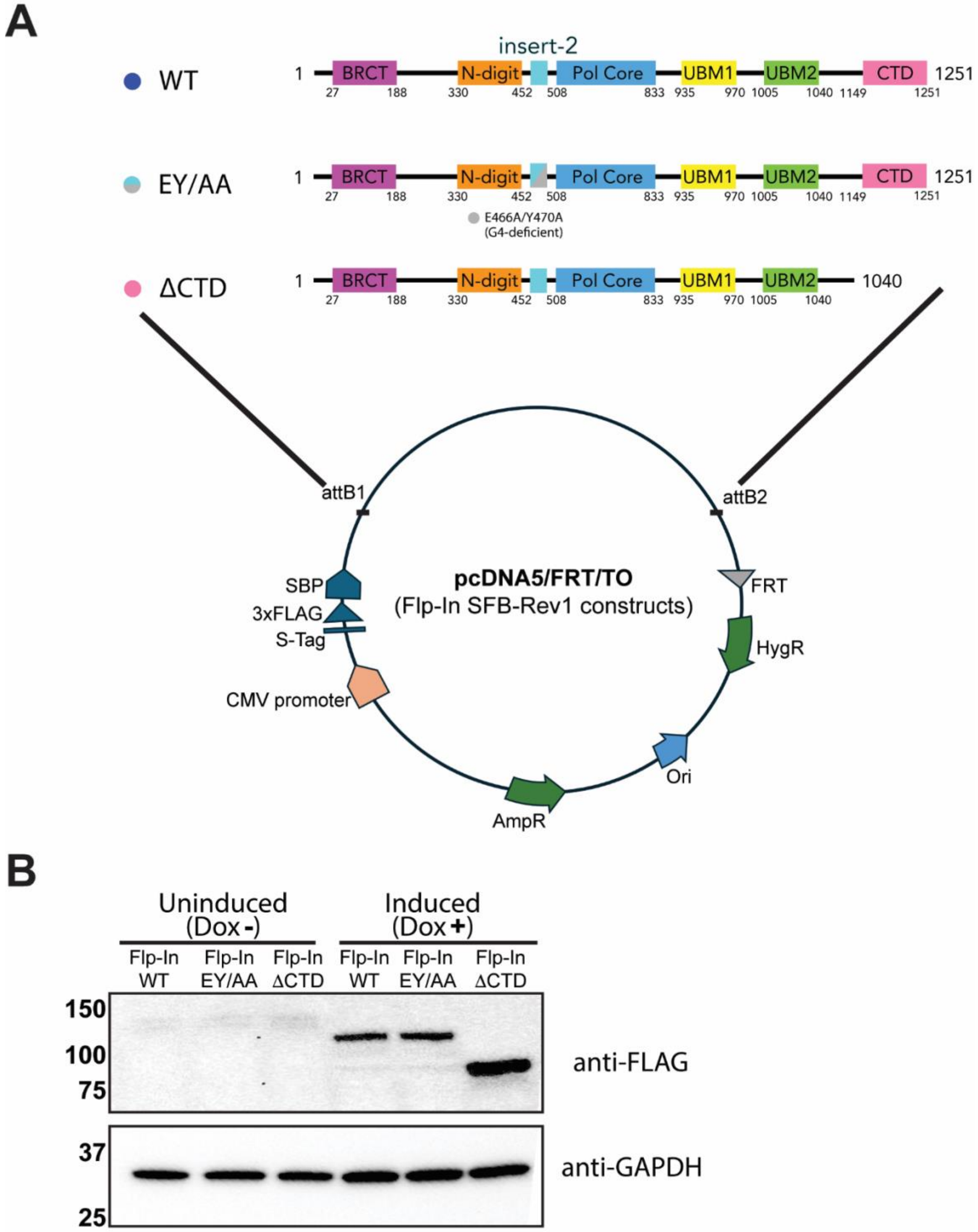

Figure S2. A. Generation of Rev1 mutant constructs and validation by immnuoblotting.

A) Cartoon schematic showing the domain organization and the region/residues mutated or deleted to generate Rev1 constructs. All SFB-Rev1 fusion constructs were cloned into the pcDNA5/FRT/TO plasmid and were used to generate stable inducible expressing 293 FT cells, in which the endogenous *REV1* gene was knocked out previously (clone 6D6; see above)

(**B**) Western blot images probed with the anti-FLAG mouse monoclonal antibody show a distinct band for each of the three REV1 fusion proteins. The CTD-deleted construct has a significantly lower molecular size as compared to the other two. The blot was also probed for GAPDH protein as a loading control.

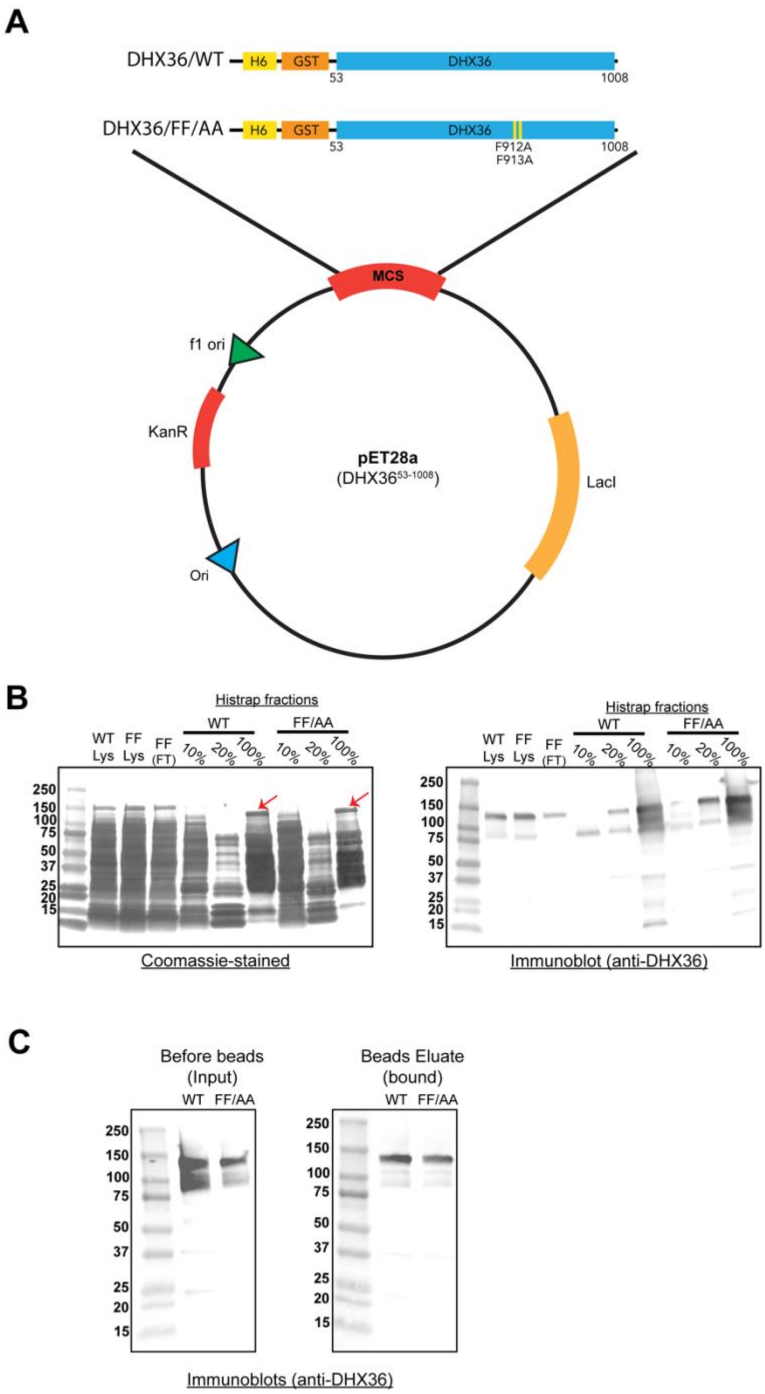

Figure S3. Design and expression of human DHX36$^{53\text{-}1008}$ WT and FF/AA constructs.

(A) The region of human wild-type (WT) DHX36 coding for amino acid residues 53 to 1008 was cloned into the multiple cloning site (MCS) of the pET28a expression vector with a N-terminal tandem hexahistidine-glutathione-S-transferase (H6-GST) affinity tag as shown. The two point mutations in residues F912 and F913 to alanines were made using the WT DHX36 plasmid as template and mutagenic primers-based PCR. The resulting mutant construct (DHX36 F912A/F913A, or FF/AA) had the mutations F912A and F913A in the putative RIR motif (described in main text).

(B) The two DHX36 constructs were overexpressed in *E. coli* BL21 (DE3) cells as described in Methods. The proteins were enriched through affinity chromatography by binding to a Ni-NTA (Histrap) column on a fast protein liquid chromatography (FPLC) system. Bound proteins were eluted using a three-step gradient of increasing imidazole concentration in the elution buffer (10%, 20% and 100% of 0.4 M). Aliquots from different fractions were checked for elution of DHX36 protein by resolving on SDS-PAGE. Samples were resolved in duplicate on two gels, one was stained using Coomassie staining solution (left panel) and the other was immunoblotted using an anti-DHX36 primary antibody (right panel). Lanes are labelled as: *WT Lys* – lysate (before enrichment) of DHX36/WT;

FF Lys – lysate (before enrichment) of DHX36/FF/AA; *FF/AA (FT)* – flowthrough (unbound fraction) of FF/AA during Histrap enrichment; 10%, 20%, 100% - fractions eluting under the respective step of imidazole gradient during elution for each DHX36 protein. Red arrows indicate the band corresponding to the DHX36 protein (expected size ~140 kDa). The immunoblot on the right was used to confirm the identity of the DHX36 band.

(C) Both the DHX36 proteins were immobilized by binding to glutathione-sepharose beads for the pull-down experiment (described in the main text). Immunoblot using anti-DHX36 antibody was used to confirm binding of the proteins to the beads. Blot on the left shows amount of each DHX36 protein in 50 μg of total protein (Histrap-enriched; before mixing with the beads) loaded. The blot on the right had aliquots of the beads after protein binding and washing, loaded on the gel by boiling the beads in 2X Laemmli buffer. Both WT and FF/AA DHX36 proteins were efficiently immobilized on the beads.

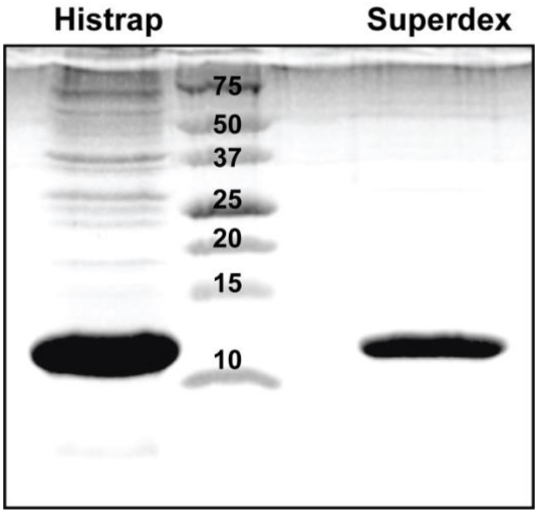

Figure S4. Expression and purification of Rev1 CTD.

The Rev1 C-terminal domain (CTD; residues 1157-1251) cloned into the pET28a expression vector was overexpressed and purified as described in Methods. The Coomassie-stained gel shows aliquots from the purification process (Histrap followed by size exclusion on a Superdex-75 column). The protein eluted after the size exclusion step was estimated to be ~95% pure by quantification using ImageJ, and was used for binding experiments using fluorescence anisotropy (described in Methods).

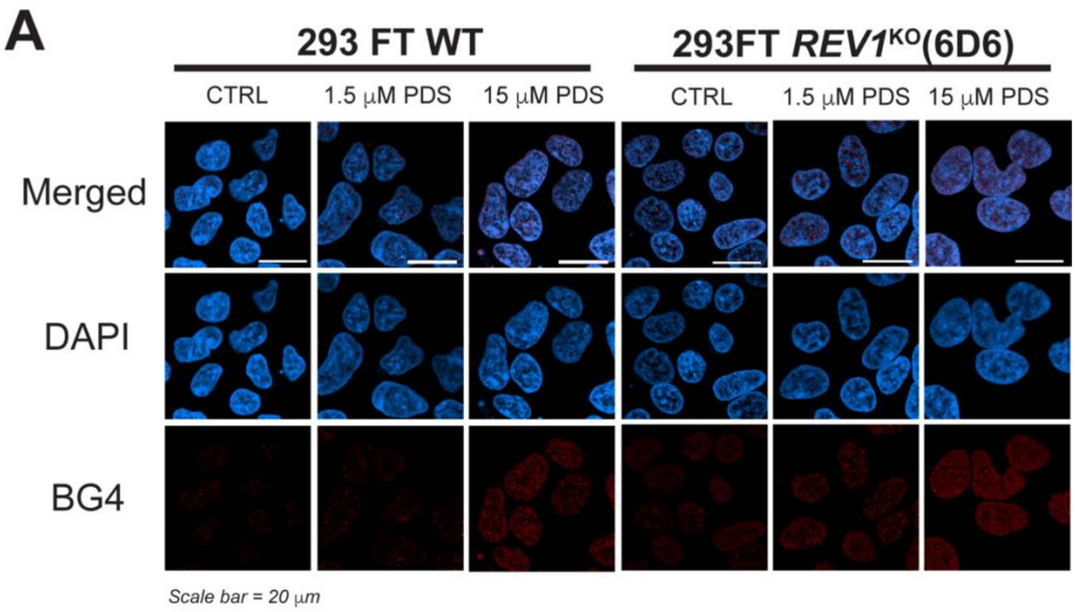

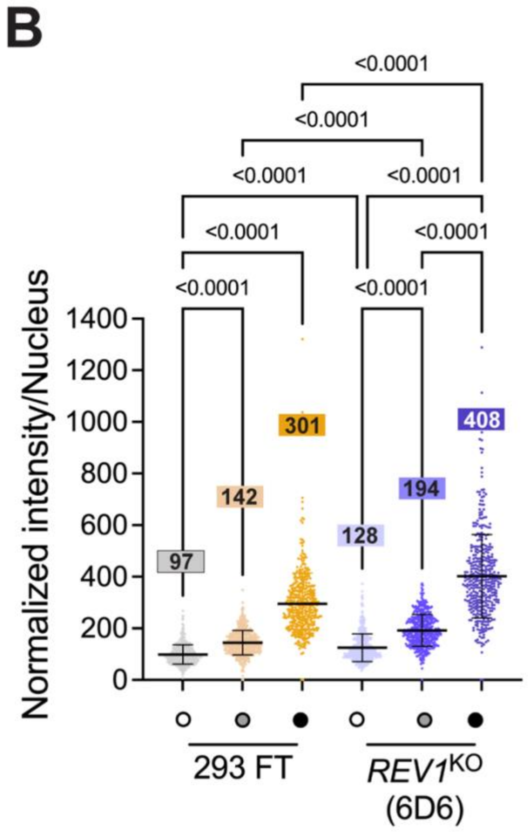

Figure S5. BG4 immunofluorescence results for 293 FT cells.

(**A**) Immunofluorescence microscopy of BG4 signal was used to monitor Rev1-dependent changes in genomic G4 content. Nuclear BG4 signal intensity was measured for untreated WT and *REV1*^{KO} (6D6) 293 FT cells and for cells treated overnight with PDS (1.5 and 15 μM). BG4 signal intensity was normalized against that observed for untreated WT 293 FT cells. Representative images are shown for the DAPI stained (blue), BG4 stained (red), and the 'merged' for both cell types under each of the three conditions. Three independent biological replicates were performed (>300 cells quantified per condition).

(**B**) Nuclear BG4 signal intensity was quantified for experiments described in panel A. Statistical significance was evaluated by performing a Kruskal-Wallis test followed by a Dunn's multiple comparisons post test. *P*-values < 0.05 are shown. Only *P*-values ≤0.05 are shown on the plot.

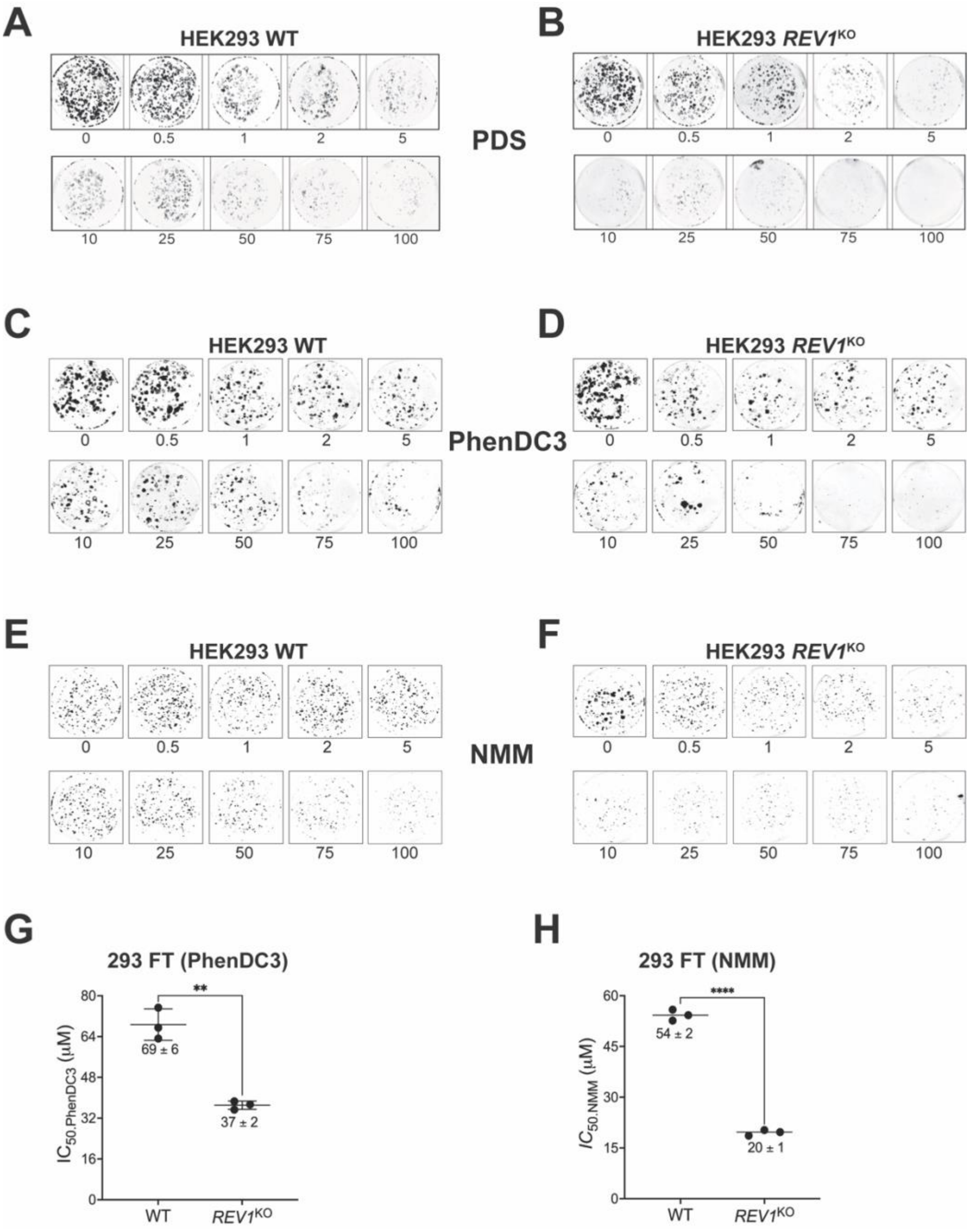

Figure S6. Representative images for clonogenic survival assay of 293 FT cells in the presence of G4 stabilizers.

Clonogenic survival assay was performed on the Parental and *REV1*^{KO} 293 FT cells to study the effect of G4 stabilizing agents PDS, PhenDC3 and NMM (as described in Methods). Representative images of colonies for both the Parental and *REV1*^{KO} HEK 293 FT cells are shown, with the concentration (0 to 100 mM) of each G4 stabilizer indicated in the center. **A** and **B** show images for cells treated with PDS, **C** and **D** for PhenDC3 and **E** and **F** for NMM respectively.

The colonies were stained with Coomassie Brilliant Blue, and images of the colonies were recorded on an Odyssey CLx imager using the Image Studio 5.2 software. Colonies were counted using the colony-counting tool in the Fiji software. Assays were performed in three biological replicates.

G and **H** show the histograms for the clonogenic assay with PhenDC3 and NMM respectively, with the mean (± std. dev.) IC_{50} values are shown for three independent biological replicates for parental WT and *REV1*^{KO} HAP1 cells treated with PDS. Statistical significance was assessed by performing an unpaired Student's t-test (***P*<0.01, *****P*<0.0001).

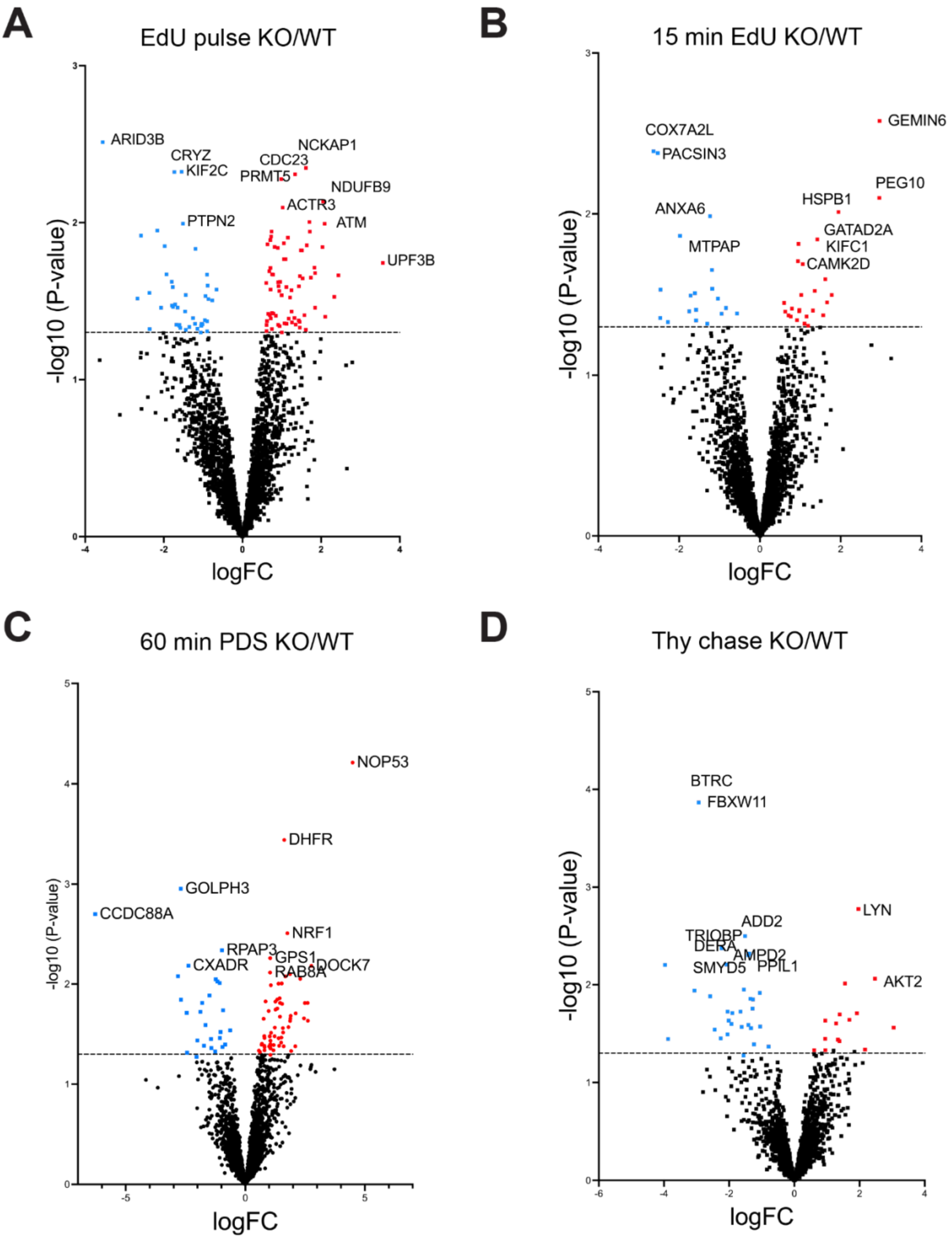

Figure S7. Analysis of differential protein abundance for parental WT and *REV1*^{KO} 293 FT iPOND-MS experiments.

Volcano plots are shown reporting on the differential protein abundance in *REV1*^{KO} and parental WT 293 FT cells. Results comparing abundance in *REV1*^{KO} divided by that observed for WT 293 FT cells are shown for the (**A**) EdU pulse (no PDS), (**B**) EdU pulse plus 15 minutes PDS, (**C**) EdU pulse plus 60 minutes of PDS, and (**D**) EdU pulse followed by thymidine chase conditions. The top 10 proteins are noted for each condition. Enriched proteins with an FDR-adjusted *P*-value ≤ 0.05 are depicted in *red*, while depleted proteins with an FDR-adjusted *P*-value ≤ 0.05 are depicted in *light blue* color.

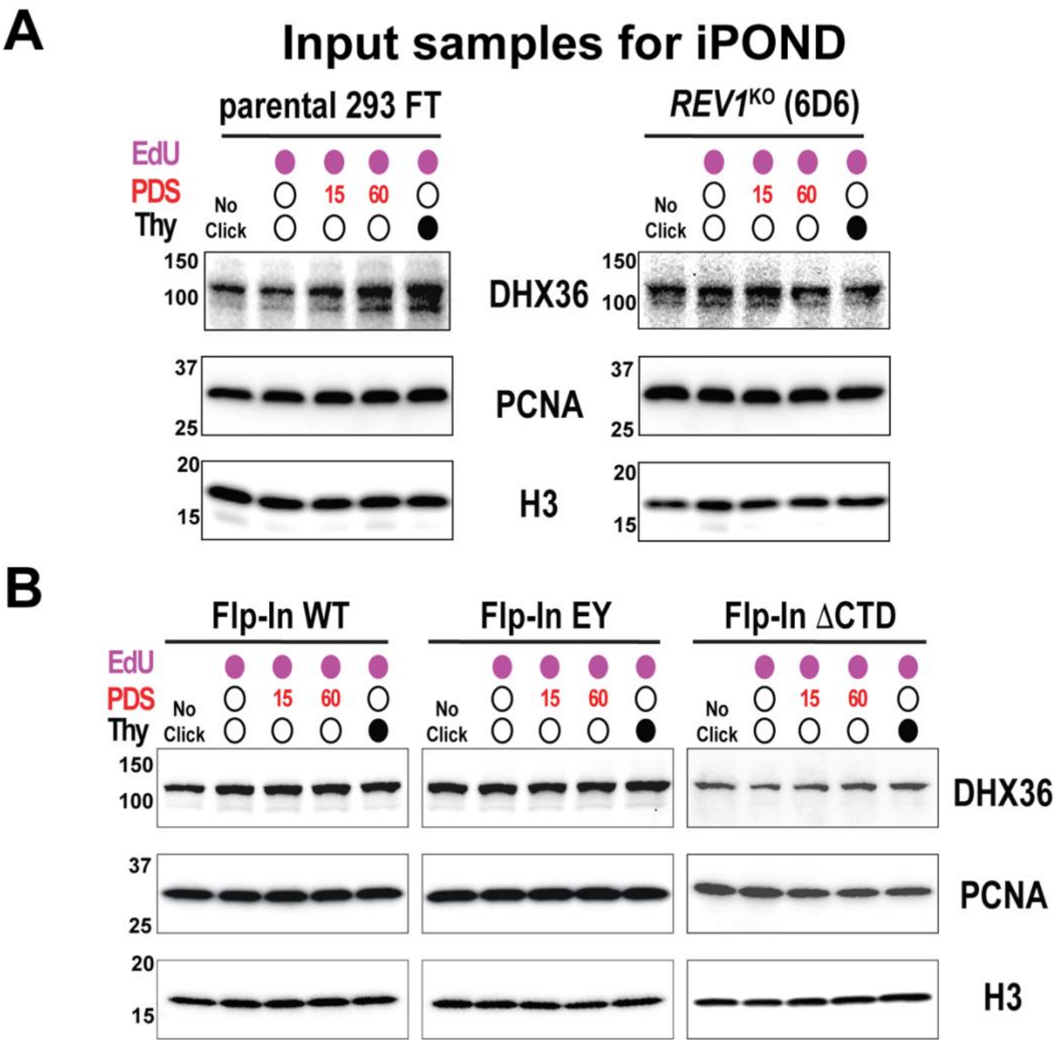

Figure S8. Immunoblots for the input samples for iPOND with parental WT and *REV1*^{KO} 293 FT cells, as well as the Flp-In cells expressing SFB-tagged REV1 proteins.

(**A**) Samples from the iPOND inputs (prior to streptavidin beads capture) for each condition were separated on SDS-PAGE gels and immunoblotted for the protein as indicated by labels for the panels. Band positions of molecular weight markers are indicated to the left of each blot. The experimental condition and cell line is indicated above each panel. The blots were probed for DHX36, PCNA and histone H3.

(**B**) Representative images for immunoblotting results for iPOND input samples for the 293 FT cell lines induced to re-express WT or mutant Rev1 (indicated above each panel) are shown. Experiments were performed in three biological replicates.

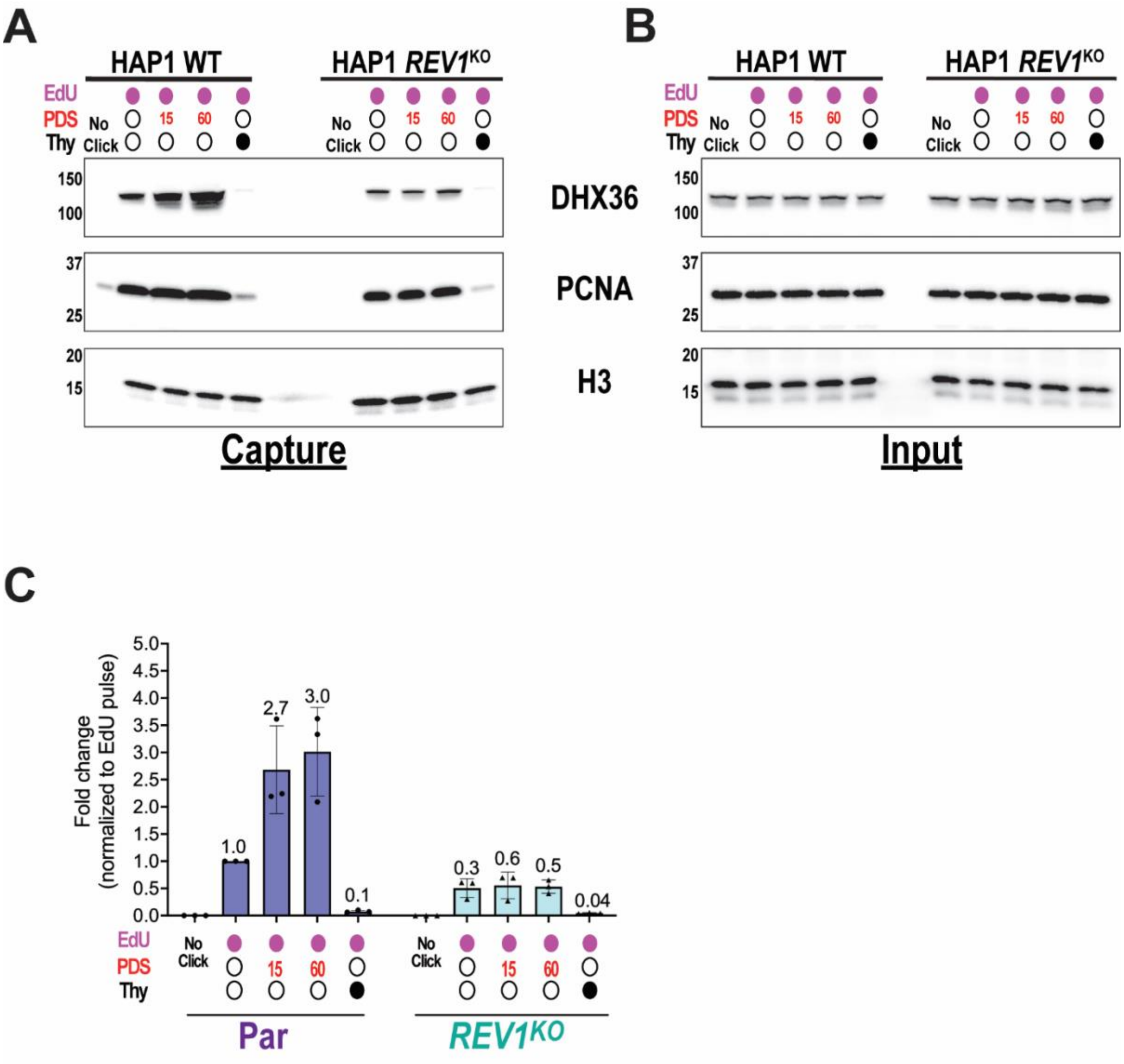

Figure S9. Immunoblots and quantification for the iPOND experiment repeated in HAP1 WT and *REV1*^{KO} cells.

The iPOND experiment (as described in Methods in 293 FT cells) was repeated using the HAP1 cells, either expressing endogeneous REV1 (Parental/WT) or the *REV1*^{KO} cells.

(**A**) DHX36 accumulation near sites of DNA synthesis ("capture" samples) was monitored in WT and *REV1*^{KO} cells by immunoblotting. Proteins captured on streptavidin beads for each condition were separated on SDS-PAGE gels and immunoblotted for the protein indicated. (**B**) Separate blots were run and probed for the indicated protein in the "input" samples from the iPOND experiment. Band positions of molecular weight markers are indicated to the left of each blot. Experiments were performed in three replicates. The experimental condition is indicated above each panel. Band intensity in each sample was measured using Fiji software.

(**C**) Quantification of DHX36 enrichment by immunoblotting for iPOND samples in WT and *REV1*^{KO} cells was performed as described in the Methods section. The DHX36 band intensity for the "no click" condition for each cell line was subtracted from the intensity values for all other conditions, following which the intensity for DHX36 in each sample was normalized to the EdU pulse sample for WT cells. Values for each condition were expressed as fold change in DHX36 compared to EdU sample. The mean (± std. dev.) is shown for three independent biological replicates. Mean values for fold-change are indicated above each condition.

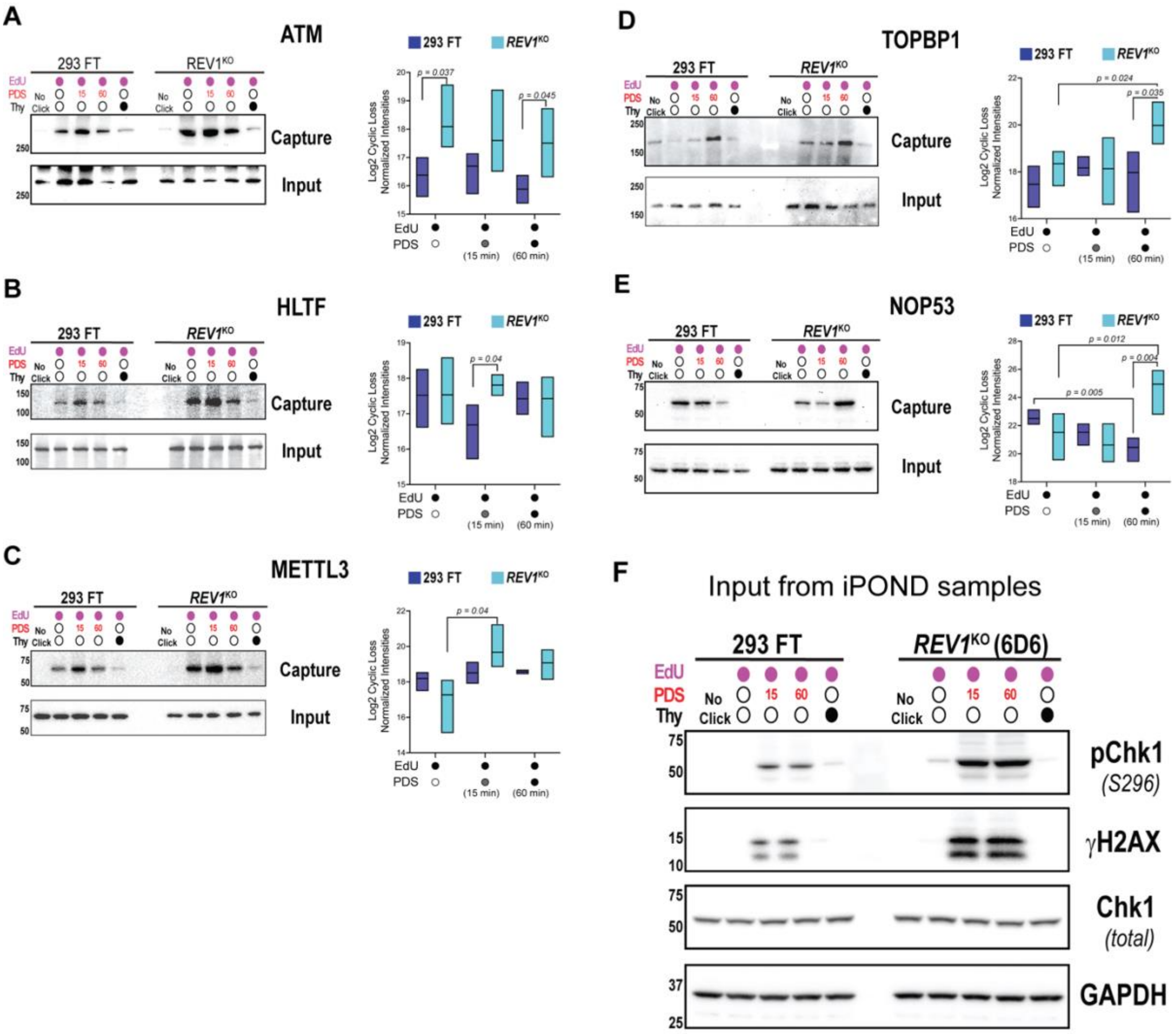

Figure S10. Validation of additional hits from the iPOND-MS experiments by immunoblotting.

The streptavidin-capture, as well as the input samples from the iPOND experiment performed in HEK 293FT and *REV1*^{KO} cells were separated on SDS-PAGE and immunoblotted as described in Methods, probing for additional targets that showed differential enrichment at the fork. Each panel (**A**-**E**) shows results from the immunoblot (left) for the capture and input samples probed for the indicated protein, and a histogram showing the normalized raw intensities (mean +/- std. dev.; n=4) from the MS data (right) for the PDS treated and control EdU pulse samples. Statistical significance between compared mean values are expressed as *P*-values, shown above each comparison. (**F**) Immunoblot results for input from iPOND samples, probing for markers of DNA damage and replication stress response activation.

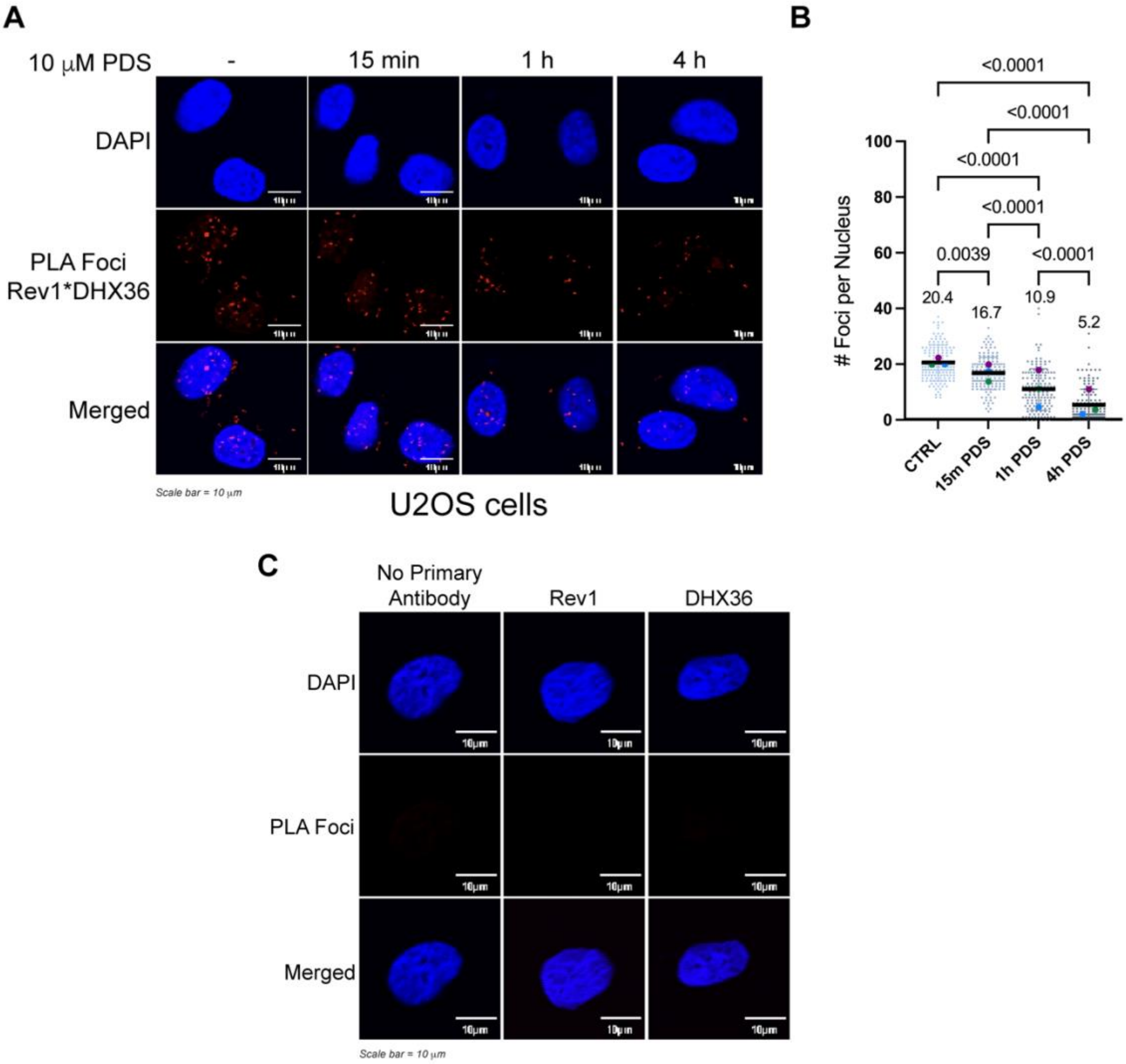

Figure S11. REV1-DHX36 PLA results from U2OS cells.

(**A**) Representative images for REV1-DHX36 PLAs conducted in U2OS cells. (**B**) The number of REV1-DHX36 foci per nucleus were quantified as described in the Methods section of the main text. Results are shown for three independent biological replicates with 50 nuclei scored per condition per replicate. The mean number of foci is shown above the data points. The mean value from each individual replicate is overlaid with individual nuclei scores and shown as a green, blue, and purple circle for each condition. Statistical significance was evaluated using a Kruskal-Wallis test followed with Dunn's multiple comparisons post test. (**C**) Control experiments were performed in the absence of any antibodies or with a single antibody added to ensure that PLA signal was specific.

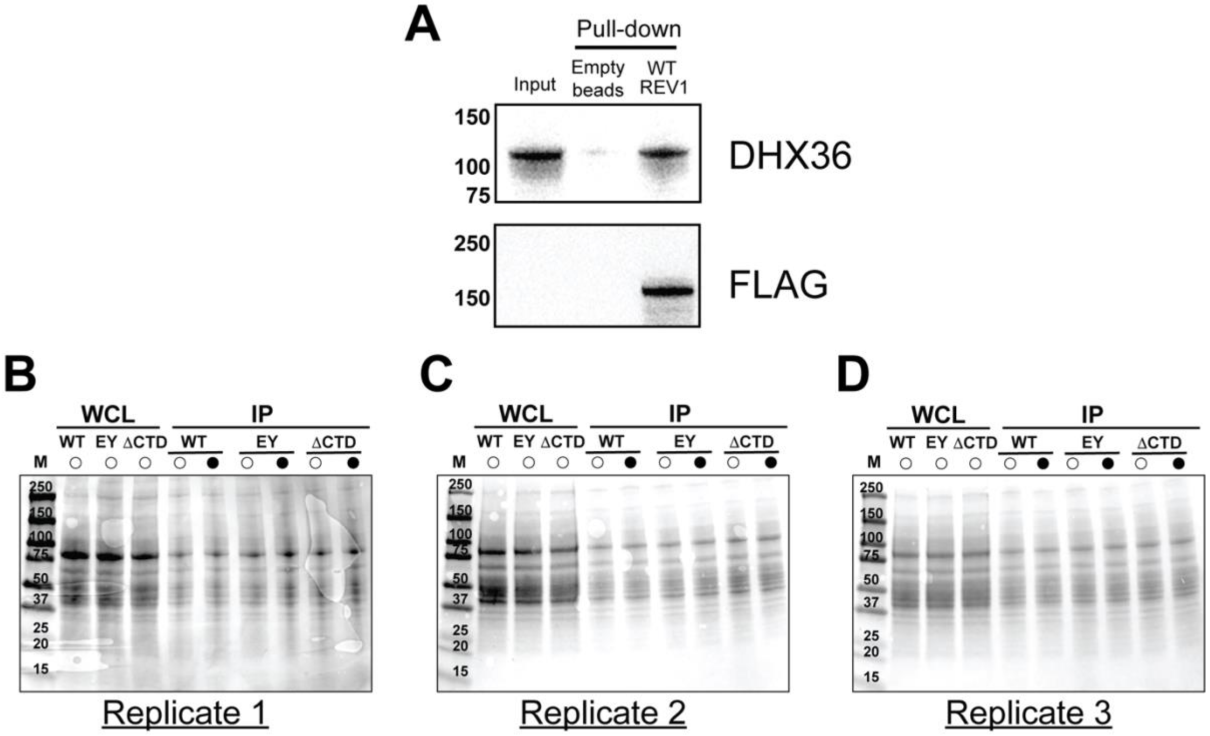

Figure S12. Control experiments for streptavidin pull-downs.

(**A**) To confirm that DHX36 did not bind to empty streptavidin (SA) beads, we incubated 200 μg of total protein from parental 293 FT cells with either empty SA beads or beads that had been pre-bound with SFB-tagged WT REV1. The pre-bound beads were incubated with 200 μg of lysate from dox-treated Flp-In 293 FT cells expressing SFB-tagged WT REV1 and washed prior to incubation with the lysates from parental 293 FT cells. The following day, the beads were pelleted, washed twice in 1X PBS, mixed with Laemmli buffer and heated to 95 °C for 10 minutes. We loaded 100 μg of protein from parental 293 FT whole cell lysates (input). Proteins were separated by SDS-PAGE and transferred to PVDF membranes. The blots were probed for DHX36 and FLAG. Almost no DHX36 signal was observed in the empty SA bead lane, while strong DHX36 eluted from beads bound with SFB-REV1 (WT).

(**B**) We used Ponceau S staining to ensure relatively equal loading of total protein in the SFB-REV1 pull-down experiments shown in Figure 8A-B of the main text. Lanes marked WCL were loaded with 50 μg of whole cell lysates for the indicated SFB-Rev1 Flp-In cells. The "IP" lanes were loaded with protein that eluted from streptavidin-coated agarose beads following incubation of lysates from cells expressing each of the three SFB-Rev1 proteins. Lanes labeled with open circles indicate control (no PDS) samples, while black circles indicate the PDS-treated samples. Lane marked **M** was loaded with the molecular weight markers (sizes labeled on each band). The blot was probed with primary antibodies against DHX36, Rev7 and PCNA (as described in Figure 4 of the main text). The blot was then stained with 0.1% (w/v) Ponceau S staining solution and was imaged to obtain bands for all lanes, in order to confirm equal loading across samples. Blots for replicate 2 (**C**) and replicate 3 (**D**) were run for samples from the other two replicates and stained similarly.

sp|Q9H2U1|DHX36_HUMAN	TKTDGLVAVHPKSVNVEQTDFHYNWLIYHLKMRTSSIYLYDCTEVSPYCLL**FF**GGDISIQ	920
tr|A0A5F8HEV5|A0A5F8HEV5_MONDO	TKTDGTVNIHPKSVNVEESEFHYNWLIYHLKMRTSSIYLYDCTEVSPYCLL**FF**GGDISIQ	921
sp|Q8VHK9|DHX36_MOUSE	TKSDGLVSIHPKSVNVEQTDFHYNWLIYHLKMRTSSIYLYDCTEVSPYCLL**FF**GGDISIQ	913
tr|F6Q4S1|F6Q4S1_HORSE	TKTDGLVALHPKSVNVEQTEFHYNWLIYHLKMRTSSIYLYDCTEVSPYCLL**FF**GGDISIQ	907
tr|H2QNM0|H2QNM0_PANTR	TKTDGLVAVHPKSVNVEQTDFHYNWLIYHLKMRTSSIYLYDCTEVSPYCLL**FF**GGDISIQ	920
tr|A0A337SLA0|A0A337SLA0_FELCA	TKTDGLVAIHPKSVNVEQTEFHYNWLIYHLKMRTSSIYLYDCTEVSPYCLL**FF**GGDISIQ	924
tr|A0A8I3PAP2|A0A8I3PAP2_CANLF	TKTDGLVAIHPKSVNVEQTEFHYNWLIYHLKMRTSSIYLYDCTEVSPYCLL**FF**GGDISIQ	903
sp|Q05B79|DHX36_BOVIN	TKTDGVVAIHPKSVNVEQTEFNYNWLIYHLKMRTSSIYLYDCTEVSPYCLL**FF**GGDISIQ	922
tr|A0A2U4BA38|A0A2U4BA38_TURTR	TKTDGLVAIHPKSVNVEQTEFHYNWLIYHLKMRTSSIYLYDCTEVSPYCLL**FF**GGDISIQ	924
tr|A0A8C6VF46|A0A8C6VF46_NAJNA	TKTDGTVNIHPKSVNVEETEFHYNWLVYHLKMRTSSIYLYDCTEVSPYCLL**FF**GGDISIQ	902
tr|A0A8C3I0Z8|A0A8C3I0Z8_CHRPI	TKTDGTVNIHPKSVNVEETEFHYNWLVYHLKMRTSSIYLYDCTEVSPYCLL**FF**GGDISIQ	888
tr|A0A151NB11|A0A151NB11_ALLMI	TKPDGTVNIHPKSVNVEETEFHYNWLVYHLKMRTSSIYLYDCTEVSPYCLL**FF**GGDISIQ	903
tr|A0A8C9N1H9|A0A8C9N1H9_SERCA	TKTDGSVNIHPKSVNVEETEFHYNWLVYHLKMRTSSIYLYDCTEVSPYCLL**FF**GGDISIQ	822
tr|U3IYV0|U3IYV0_ANAPP	TKTDGTVNIHPKSVNVEETEFHYNWLVYHLKMRTSSIYLYDCTEVSPYCLL**FF**GGDISIQ	901
tr|A0A1D5PHB6|A0A1D5PHB6_CHICK	TKTDGTVNIHPKSVNVEETEFHYNWLVYHLKMRTSSIYLYDCTEVSPYCLL**FF**GGDISIQ	899
tr|A0A182XL20|A0A182XL20_ANOQN	IEGLGRAEIHPGSVNGNRGVFHSNFVVYYDMQKINALTIFDTTVVNPFPLL**FF**GDCHVE-	971
tr|A0A8M1NHJ3|A0A8M1NHJ3_DANRE	TKADGKVCIHPKSVNAEETQFQYKWLVYHLKMKTTSIFLYDCTEVSPFSLL**FF**GGNISIQ	944
tr|Q8SWT2|Q8SWT2_DROME	TDDGRRVNFHPSSVNSGESGFDSAYFVYFQRQKSTDLFLLDSTMVFPMALIIFGDGVEAG	859
tr|A0A453DRY4|A0A453DRY4_AEGTS	TMEDGQVMLYSSSVNGKEAKIPFPWLVFNEKVKVNSVFLRDSTAISDSILLLFGGNIQQG	800

Figure S13. DHX36 sequence alignment.

The primary amino acid sequence of DHX36 from *Homo sapiens* (Human; UniProt ID: Q9H2U1), *Monodelphis domestica* (Gray short-tailed opossum; UniProt ID: A0A5F8HEV5), *Mus musculus* (Mouse; UniProt ID: Q8VHK9), *Equus caballus* (Horse; UniProt ID: F6Q4S1), *Pan troglodytes* (Chimpanzee; UniProt ID: H2QNM0), *Felis silvestris catus* (Cat; UniProt ID: A0A337SLA0), *Canus lupus familiaris* (Dog; UniProt ID: A0A8I3PAP2), *Bos taurus* (Bovine; UniProt ID: Q05B79), *Tursiops truncates* (Atlantic bottle-nosed dolphin; UniProt ID: A0A2U4BA38), *Naja naja* (Indian cobra; UniProt ID: A0A8C6VF46), *Chrysemys picta bellii* (Western painted turtle; UniProt ID: A0A8C3I0Z8), *Alligator mississippiensis* (American alligator; UniProt ID: A0A151NB11), *Serinus canaria* (Island canary; UniProt ID: A0A8C9N1H9), *Anas platyrhynchos platyrhynchos* (Northern mallard; UniProt ID: U3IYV0), *Gallus gallus* (Chickent; UniProt ID: A0A1D5PHB6), *Anopheles quadriannulatus* (Mosquito; UniProt ID: A0A182XL20), *Dario rerio* (Zebrafish; UniProt ID: A0A8M1NHJ3), *Drosophila melanogaster* (Fruit fly; UniProt ID: Q8SWT2), and *Aegilops tauschii* subsp. *strangulata* (Goatgrass; UniProt ID: A0A453DRY4) were aligned using the Clustal alignment tool. Only the part of the sequence alignment with the putative RIR motif is shown to illustrate that the double-phenylalanine is broadly conserved across metazoans, with some variation apparent in fruit flies and goatgrass.

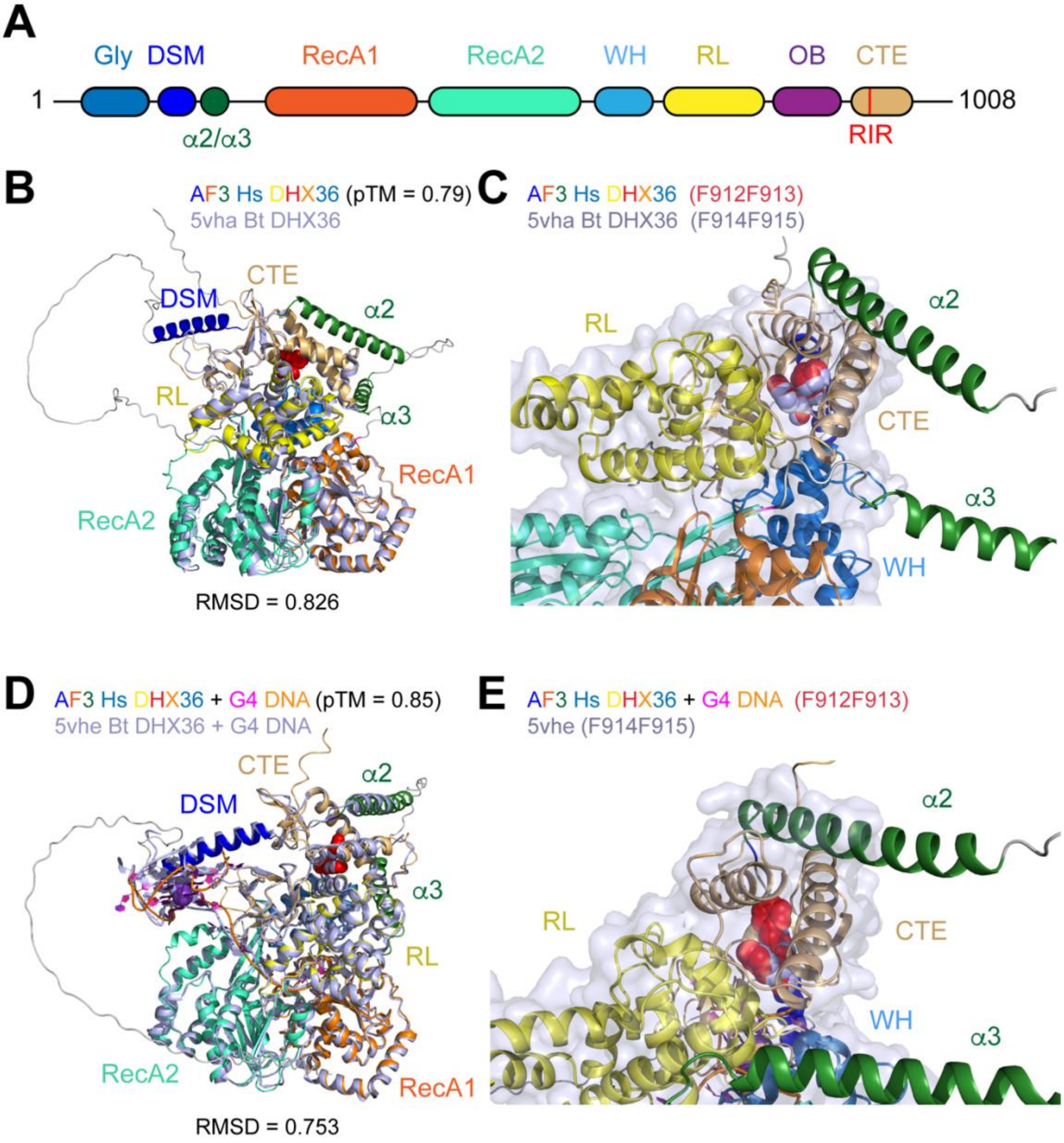

Figure S14. Position of putative RIR in apo- and G4-bound DHX36 structures.

(**A**) A schematic diagram of the DHX36 primary sequence with domains identified is shown.

(**B**) An AlphaFold3-generated model of human DHX36 was superimposed with the structure of bovine DHX36 (PDB ID: 5vhe) reported by Chen MC et al. in 2018 (PMCID: PMC6261253). The RMSD from the superimposition is noted on the panel. The structures are shown in cartoon form and the domain colors for the AF3 model match those used in panel A. Panel **C** provides a closer view of the region where the DHX36 RIR is located. The side-chains of the two central phenylalanine residues are shown as spheres (*light blue* for the 5vhe structure and *red* for the AF3 model). The main take away is that the DHX36 RIR is inaccessible to the REV1 CTD in the conformation shown here.

(**D**) Similar to panels B and C, an AlphaFold3-generated model of human DHX36 was superimposed with the structure of bovine DHX36 (PDB ID: 5vhe) reported by Chen MC et al. in 2018 (PMCID: PMC6261253). These structures both contain a Myc-derived ssDNA substrate that adopts a parallel-stranded G4 structure (5′-AGG GTG GGT AGG GTG GGT TTT TTT-3′). Like the apo structures, the central phenylalanine residues of the DHX36 RIR remain inaccessible. Panel **E** shows a closer view of the RIR. The interaction with the REV1 CTD we report in the main text would seem to require that DHX36 undergo a conformational change to make the RIR accessible.
